# Dehydrin Client Proteins Identified Using Phage Display Affinity Selected Libraries Processed With Paired-End Phage Sequencing

**DOI:** 10.1016/j.mcpro.2024.100867

**Published:** 2024-10-21

**Authors:** Sandra Helena Unêda-Trevisoli, Lynnette M.A. Dirk, Francisco Elder Carlos Bezerra Pereira, Manohar Chakrabarti, Guijie Hao, James M. Campbell, Sai Deepshikha Bassetti Nayakwadi, Ashley Morrison, Sanjay Joshi, Sharyn E. Perry, Vijyesh Sharma, Caleb Mensah, Barbara Willard, Laura de Lorenzo, Baseerat Afroza, Arthur G. Hunt, Tomokazu Kawashima, Lisa Vaillancourt, Daniel Guariz Pinheiro, A. Bruce Downie

**Affiliations:** 1 Department of Horticulture, Martin-Gatton College of Agriculture, Food and Environment, University of Kentucky, Lexington, Kentucky, USA; 2 Seed Biology Program, University of Kentucky, Lexington, Kentucky, USA; 3 Department of Crop Production, São Paulo State University (Unesp), School of Agricultural and Veterinarian Sciences, São Paulo, Brazil; 4 Pastotech Pasture Seeds, Campo Grande, Mato Grosso do Sul, Brazil; 5 School of Integrative Biological and Chemical Sciences, University of Texas Rio Grande Valley, Edinburg, Texas, USA; 6 Department of Plant and Soil Science, Martin-Gatton College of Agriculture, Food and Environment, University of Kentucky, Lexington, Kentucky, USA; 7 Catalent Pharma Solution, Baltimore, Maryland, USA; 8 University of Kentucky Agricultural and Medical Biotechnology Program, Lexington, Kentucky, USA; 9 Department of Toxicology and Cancer Biology, College of Medicine, University of Kentucky, Lexington, Kentucky, USA; 10 Kentucky Tobacco Research and Development Center, Lexington, Kentucky, USA; 11 Carter G. Woodson Academy, Fayette County Public Schools (FCPS), Lexington, Kentucky, USA; 12 Department of Biochemistry and Molecular Biology, University of New Mexico, School of Medicine, Albuquerque, New Mexico, USA; 13 Division of Vegetable Science, SKUAST- Kashmir, Srinagar, Kashmir, India; 14 Department of Plant Pathology, Martin-Gatton College of Agriculture, Food and Environment, University of Kentucky, Lexington, Kentucky, USA; 15 Department of Agricultural, Livestock and Environmental Biotechnology, São Paulo State University (UNESP), School of Agricultural and Veterinary Sciences, Jaboticabal, São Paulo, Brazil

**Keywords:** late embryogenesis abundant proteins, phage display, client proteins, paired-end sequencing, temperature related intensity change assay

## Abstract

The late embryogenesis abundant proteins (LEAPs) are a class of noncatalytic, intrinsically disordered proteins with a malleable structure. Some LEAPs exhibit a protein and/or membrane binding capacity and LEAP binding to various targets has been positively correlated with abiotic stress tolerance. Regarding the LEAPs’ presumptive role in protein protection, identifying client proteins (CtPs) to which LEAPs bind is one practicable means of revealing the mechanism by which they exert their function. To this end, we used phage display affinity selection to screen libraries derived from *Arabidopsis thaliana* seed mRNA with recombinant orthologous LEAPs from Arabidopsis and soybean (*Glycine max*). Subsequent high-throughput sequencing of DNA from affinity-purified phage was performed to characterize the entire subpopulation of phage retained by each LEAP ortholog. This entailed cataloging in-frame fusions, elimination of false positives, and aligning the hits on the CtP scaffold to reveal domains of respective CtPs that bound to orthologous LEAPs. This approach (paired-end phage sequencing) revealed a subpopulation of the proteome constituting the CtP repertoire in common between the two dehydrin orthologs (LEA14 and GmPm12) compared to bovine serum albumin (unrelated binding control). The veracity of LEAP:CtP binding for one of the CtPs (LEA14 and GmPM12 self-association) was independently assessed using temperature-related intensity change analysis. Moreover, LEAP:CtP interactions for four other CtPs were confirmed *in planta* using bimolecular fluorescence complementation assays. The results provide insights into the involvement of the dehydrin Y-segments and K-domains in protein binding.

## Highlights

• Paired-end phage sequencing filters Illumina tags to identify in-frame peptides.
• Orthologous dehydrins bind a common, discrete client protein repertoire.
• Temperature related intensity change quantifies dehydrin:client protein binding.
• Dehydrin:client protein interactions confirmed *in vivo*.

## Graphical Abstract

**Figure:**
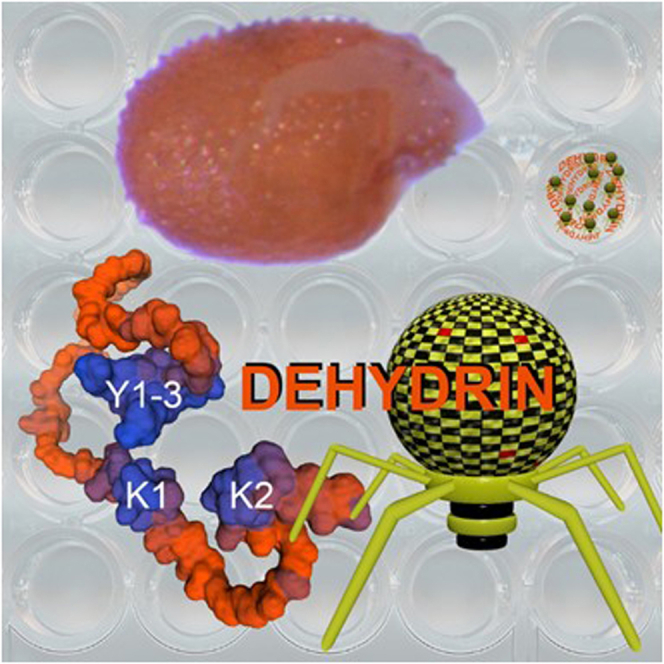

## In Brief

Intrinsically disordered dehydrin (DHN) proteins are hypothesized to bind to other (client) proteins. However, the scope and significance of the DHN-client association remains poorly understood. A novel combination of T7 phage display accompanied by high-throughput DNA sequencing was used to identify a repertoire of Arabidopsis protein clients of two DHN orthologs. A subset of clients demonstrated binding *in planta*. The results affirm the utility of high-throughput approaches and reveal novel DHN clients.

Anhydrobiosis is a fascinating attribute of select organisms associated with extreme longevity (1, 2) and the capacity to exist in extreme environments (3). This aptitude depends on the ability to dehydrate without loss of function, a complex and poorly understood phenomenon (4, 5). A family of intrinsically disordered proteins, the late embryogenesis abundant proteins ((6); also known as LEAPs), have a demonstrated competence to protect against desiccation across multiple kingdoms of the tree of life (7, 8, 9, 10). This protective functionality has been attributed to the capacity of some LEAPs to act as “molecular shields” seemingly preventing catastrophic aggregation of partially unfolded proteins without physically binding to them as water becomes scarce (11). There are other instances where a physical association of LEAPs with client proteins (CtPs) has been documented (*e.g.*, (12)). For many of these interactions the consequences of LEAP:CtP binding remain obscure (reviewed in (13)).

LEAPs do not usually have a defined shape in solution, and whether, and to which, proteins they physically bind is largely unknown. The mechanisms by which physical association with LEAPs may apply a protective influence over members of a stress-susceptible proteome are also poorly understood. As such the LEAPs constitute members of the “dark proteome” (14) one hallmark of which is a paucity of knowledge of their binding repertoire (15). Because LEAPs lack any known catalytic site and often exist in an intrinsically disordered state in an aqueous milieu, one means of acquiring information on their possible modes of action and their protein partner preferences, is to catalog the repertoire of their CtPs. Understanding how specific LEAPs bind to their CtPs lags considerably behind that of how other LEAPs bind to membranes (16, 17, 18).

In this report, we describe the adaptation of phage display for cataloging possible CtPs to two orthologous LEAPs. Phage display entails the presentation of nonviral proteins tethered to the surface of the bacteriophage in such a way as to make these proteins available for interactions with immobilized bait proteins. Typically, this is accomplished by creating a complementary DNA (cDNA) library in the context of a viral coat protein, such that a replication-competent virus deploys a surface protein chimera with some of the organism-of-interest’s proteome solvent exposed (19). Phage display is a very high throughput method (20) to not only identify CtPs but also determine regions of interaction between the target and the protein of interest by examining the diversity of poly-peptide fragments encoded by independent virus retrieved for any particular protein recovered in the screen (21). The resilience of phage to a variety of chemical insults and extreme environments, and a lack of an endogenous metabolism (22) allows protein–protein screens over a wide array of stressful conditions in solutions containing a variety of metabolites (23, 24).

Here we report the CtP repertoire of orthologous LEAPs of the dehydrin (DHN) family discovered by high-throughput sequencing an expansive cross-section of the DHN-selected, phage subpopulation. The Arabidopsis DHN and its soybean ortholog were separately tested using temperature-related intensity change (TRIC) for their capacity to auto-associate. Similarly, bimolecular fluorescence complementation (BiFC) assays (specifically, split YFP fusions) were used to demonstrate binding of 4 of 5 chosen LEA14:CtP pairs *in planta*. Using the paired-end sequences, the regions on CtPs to which the DHNs had affinity were determined.

## Experimental Procedures

### Cloning Soybean and Arabidopsis DHN Orthologs

**Fig. 1:**
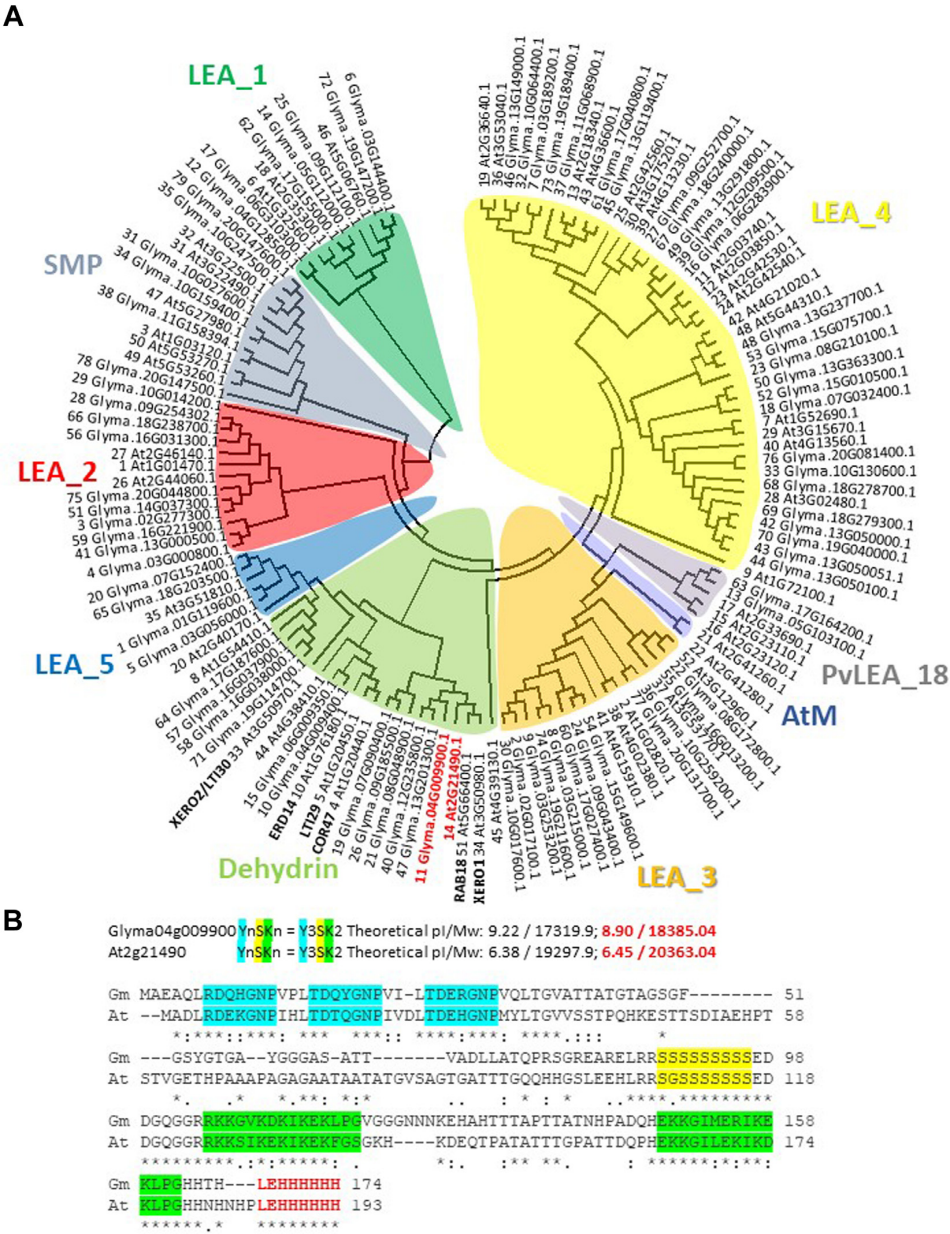
**Phylogenetic and sequence analysis of late embryogenesis abundant proteins.***A*, an alignment of the different LEAP families (6) from both soybean (79 proteins) and Arabidopsis (52 proteins). The supplemental text details the rationale for the specific sequences used. The soybean LEAPs are numbered by ascending chromosome and gene as done previously for Arabidopsis (6). *B*, the amino acid sequence of two orthologous LEAPs of the DHN family (AT2G21490 and Glyma.04G009900) of the type YnSKn, where n = 3 for the Y-segment and n = 2 for the K-segment (108). Both have a carboxy-terminal, hexahistidyl tag with a vector-derived “LE” amino acid linker to the ultimate carboxy-terminal amino acid in the native DHN sequence. These extraneous amino acids are in *red text*; as are the changes in the theoretical pI and molecular weight (Mw) that these additions made to the proteins. Clustal Omega (109) was used to generate the amino acid sequence alignment. DHN, dehydrin.

The DHN orthologs used in this study were identified from the consensus tree (Fig. 1*A*; Supplemental Text) and contain 3 Y-domains, 1 S-domain, and 2 K-domains in native proteins of 166 (soybean) and 185 (Arabidopsis) aa (Fig. 1*B*). RNA was extracted from imbibed soybean (*Glycine max*) seed (cv Williams) using the pine tree method (25), and treated with DNase (DNA-*free*; Thermo Fisher Scientific) prior to being reverse-transcribed (SuperScript III; Thermo Fisher Scientific). The resultant cDNA was used as template in a PCR with Easy-A high-fidelity PCR cloning enzyme (Agilent Technologies, Inc) and gene-specific primers to the coding region of DHN GmPM12 (Glyma.04G009900.1). NdeI and XhoI sites were incorporated into the forward and reverse primers, respectively, (Supplemental Table S1) and thymine (TA) cloning was used to introduce the amplicon using T4 DNA ligase (New England BioLabs) into a TA cloning vector (pNCO1T). The DHN sequence was excised from pNCO1T using NdeI and XhoI and subcloned into similarly digested pET23b (Novagen-Sigma Aldrich-EMD Group). Individual clones were isolated and sequenced and one housing the intact *DHN* sequence was selected for expression and protein purification.

A pUNI51 clone (U13725 (26); ABRC) for the Arabidopsis (*Arabidopsis thaliana*) DHN ortholog of GmPM12, LEA14 (*AT2G21490*) (6) was used as a PCR template with NdeI- and PspXI site–containing, gene-specific forward and reverse primers, respectively, as described for GmPM12 (Supplemental Table S1). The amplicon was cloned into, and sequenced from, pNCO1T prior to subcloning the LEA14 coding region using NdeI and PspX1 into NdeI and XhoI-digested pET23b (Novagen) as described for GmPM12. Sequencing confirmed that the vector-encoded carboxy-terminal hexahistidyl tag was in-frame in both DHN coding sequences.

### Protein Expression and Purification

The soybean DHN GmPM12 was expressed from pET23b in Rosetta (Novagen) cells. Five milliliters of an overnight LB culture (100 μg ml^−1^ ampicillin and 34 μg ml^−1^ chloramphenicol) was used to seed 1 L of LB (100 μg ml^−1^ ampicillin, 34 μg ml^−1^ chloramphenicol). Cells were grown for 12 h at 37 °C without induction, relying on leaky expression, at which time cells were harvested and processed for protein purification. The Arabidopsis DHN LEA14 was expressed from pET23b in BL21 Star (DE3) pLysS cells. Five milliliters of an overnight culture (in LB + 100 μg ml^−1^ ampicillin and 34 μg ml^−1^ chloramphenicol) was used to inoculate 1 L of LB (+100 μg ml^−1^ ampicillin, 34 μg ml^−1^ chloramphenicol) and the cultures were grown at 37 °C to an *A*_600_ of 2.0. At this point, IPTG was added to a final concentration of 0.4 mM, and cultures were grown for an additional hour before harvesting for protein purification.

Cells were harvested by centrifugation (10,000*g*, 10 min, 4 °C) and the spent media removed from the pellet. Cells were resuspended in 10 ml 10 mM Tris–HCl, pH 7.5, placed in a 25 ml polypropylene centrifuge bottle, and boiled for 20 min. Following boiling, the suspension was centrifuged at 12,000*g* for 10 min at 4 °C.

**Fig. 2:**
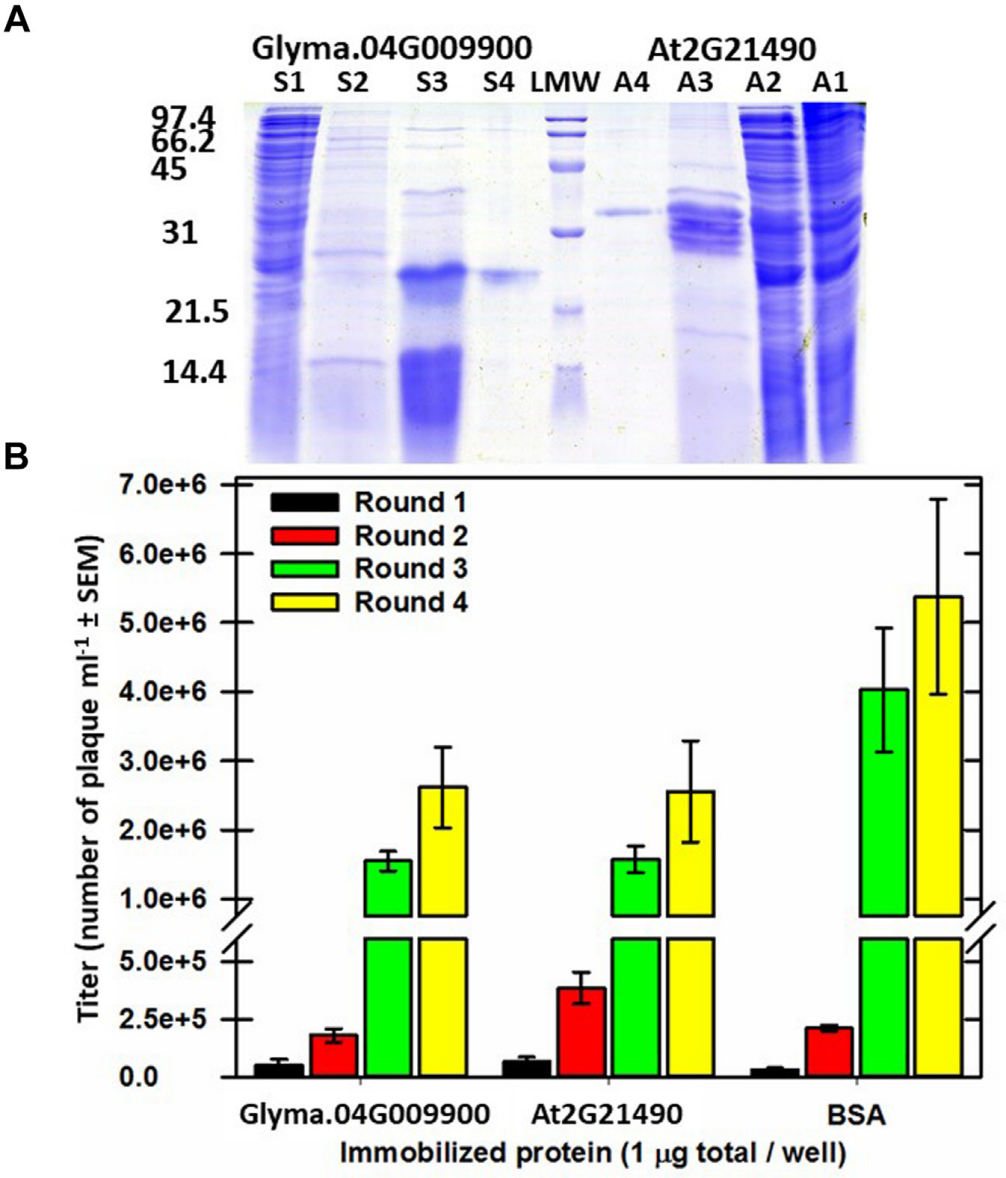
**Recombinant protein expression and purification for use in phage display affinity selection.***A*, the recombinant proteins were prepared as described in Methods and analyzed by SDS-PAGE (15% gel). In A, (S1: soybean, A1: Arabidopsis) uninduced soluble fraction; (S2, A2) IPTG (0.4 mM)-induced soluble fraction; (S3, A3) boiled samples; (S4, A4) Nickel column, affinity-purified hexahistidyl-tagged recombinant protein; low range molecular weight (LMW) markers (kDa). It was determined that IPTG induction was unnecessary for GmPM12 accumulation, and this step was subsequently dropped. *B*, titers (means with SEM) of libraries after each round of selection with a DHN or bovine serum albumin (BSA) using an Arabidopsis seed library in the T7Select10-3b vector. Plating was conducted in triplicate dilutions of the affinity-selected library recovered from 3 independent microtiter plate wells and plaques were counted from serial dilutions. DHN, dehydrin.

DHN-containing supernatants were passed through a 0.45 μm filter and loaded onto nickel-charged, prewashed, Hi-Trap chelating HP (GE Healthcare Life Sciences) columns. Each column was attached to an ÄKTA Avant fast performance liquid chromatography (GE Healthcare Bio-Sciences AB) and washed extensively with 10 mM Tris–HCl buffer, pH 7.5 until the *A*_280_ stabilized. Bound proteins were eluted at 1 ml • min^-1^ with a linear gradient of imidazole (0 to 1 M) in 10 mM Tris–HCl, pH 7.5 applied over 10 min to the column while 1 ml fractions were collected. Aliquots of these fractions were assessed using SDS-PAGE (15% total acrylamide) (Fig. 2*A*). Aliquots with pure recombinant protein were pooled and dialyzed extensively against 10 mM Tris–HCl, pH 7.5. Dialyzed aliquots were then quantified, dispensed into 0.5 ml microtubes, and snap-frozen in liquid nitrogen and stored at −80 °C until use.

### Affinity Selection of DHN CtPs from Phage Display Libraries

RNA was acquired using a hot borate technique (27) from mature, dehydrated, quiescent (0) or 12-, 24-, or 36-h-germinated (on water at 25 °C with constant light before completion of germination) *Arabidopsis thaliana* (Columbia) seeds. This RNA was then used to generate the phage display library from polyA + mRNA, gathered after two rounds of selection over Oligotex resin (QIAGEN Inc). For the library, 1 μg of PolyA + mRNA from each germination stage was combined for cDNA synthesis. One μg of random primers (Hind III RP) was used to prime first- and second-strand synthesis using a kit (OrientExpress random primer cDNA synthesis kit; Novagen). End modification using T4 DNA polymerase, linker ligation to the cDNAs, digestion with Eco RI/Hind III, and size fractionation were according to the instructions of the kit manufacturer (T7Select10-3 Cloning Kit, Novagen). The cDNAs were ligated into T7Select10-3b vector arms, the library packaged into bacteriophage T7 *in vitro*, and an aliquot used for serial dilutions to infect the *Escherichia coli* strain BLT5403 to determine the titer of the primary recombinants. The primary library was amplified using plate lysates and the phage extraction buffer (20 mM Tris, pH 8.0, 100 mM NaCl, 6 mM MgSO_4_) from the plates was combined, treated with chloroform, centrifuged (3000*g*, 5 min), and the supernatant recovered. The library was mixed with 0.1 volume 80% glycerol, and 1 ml aliquots were prepared and stored at −80 °C.

For each round of affinity selection, individual wells in 96-well microtiter plates (clear, flat-bottom, standard tissue culture surface; Corning Incorporated) were color coded with permanent ink on the underside of three wells per protein (9 wells total). The plate was washed extensively with water, after which 10 μg ml^−1^ of (a) recombinant GmPM12; (b) recombinant LEA14 or; (c) purchased bovine serum albumin (BSA; Sigma-Aldrich), all in 0.1 ml of 10 mM Tris, pH 7.5, were added to each of the three similarly marked wells for each protein (this yields three replications for each affinity selection). The dishes were covered with plastic film and left overnight at 4 °C. Unbound protein was removed by washing 10 times with 0.2 ml each time of Tris-buffered saline (TBS; 0.1 M Tris–HCl, 0.5 M sodium chloride, pH 7.5) before blocking with 0.2 ml 5% (w/v) blocking reagent (protein assay estimated 4 mg protein per well; EMD Chemicals Inc) in TBS for 1 h at room temperature, wrapped in plastic film. Excess blocking reagent was removed by washing the wells 10 times with 0.2 ml each time of TBS with 0.05% v/v Tween 20 (TBST, Sigma-Aldrich) before introducing the naïve phage library into each well. The plates were resealed with plastic film and placed at 25 °C for 1 h on a rotary platform at 50 RPM. After 1 h, wells were washed with 10 × 0.2 ml aliquots of 25 °C TBST removing the TBST each time by slamming the plate onto paper towel. After the tenth wash, 0.1 ml of BLT5403 cells were placed in the bottom of each well. The plates were wrapped in plastic film and incubated at 37 °C for 20 min to allow any retained phage to infect the bacteria.

At the end of 20 min, the plates were unwrapped. Three 10 μl aliquots (replicates) of bacteria from each well were placed in separate tubes of 0.99 ml LB media with 100 μg μl^−1^ ampicillin for titering. The remaining 70 μl of BLT5403 bacteria in each well was added to 50 ml BLT5403 cells that had been grown to 0.6 to 1.0 *A*_600_ in 0.5 l Erlenmeyer flasks. These mixtures were returned to 37 °C with agitation for phage amplification and lysate production. Upon lysis, the culture was brought to 0.5 M NaCl and centrifuged at 8000*g* for 10 min at 25 °C. The supernatant was transferred to clean, labeled tubes, a few drops of chloroform added, and the lysate was stored at 4 °C until the next round of affinity selection (28).

### Titering

Serial dilutions of the cells from each replicate were made and 0.1 ml of these dilutions were added to 0.25 ml of BLT5404 cells. To these cells was added 3 ml of top agarose which was then quickly spread over 100 μg ml^−1^ ampicillin-containing LB plates. Dishes were incubated at 23 °C overnight and plaques counted the following day. The plaque-forming units·ml^−1^ were calculated from the dilution and titer was ascertained (Fig. 2*B*).

### Paired-end phage sequencing

It was determined that PEG 8000 precipitation of phage prior to Illumina-adaptor-tagged library construction provided a greater size range of amplicons and a greater amplicon abundance than unprecipitated phage lysate (Supplemental Fig. S1). Therefore, PEG purification was used throughout by adding one-fifth volume of 50% PEG 8000 to each aliquot before mixing and keeping on ice overnight at 4 °C, followed by centrifugation at 7000*g* 10 min at 4 °C. The supernatant was removed by aspiration and the pellet left to drain by inverting the microtube for 20 min. The pellet was then resuspended in 0.2 ml water and used as a template for PCR.

**Fig. 3:**
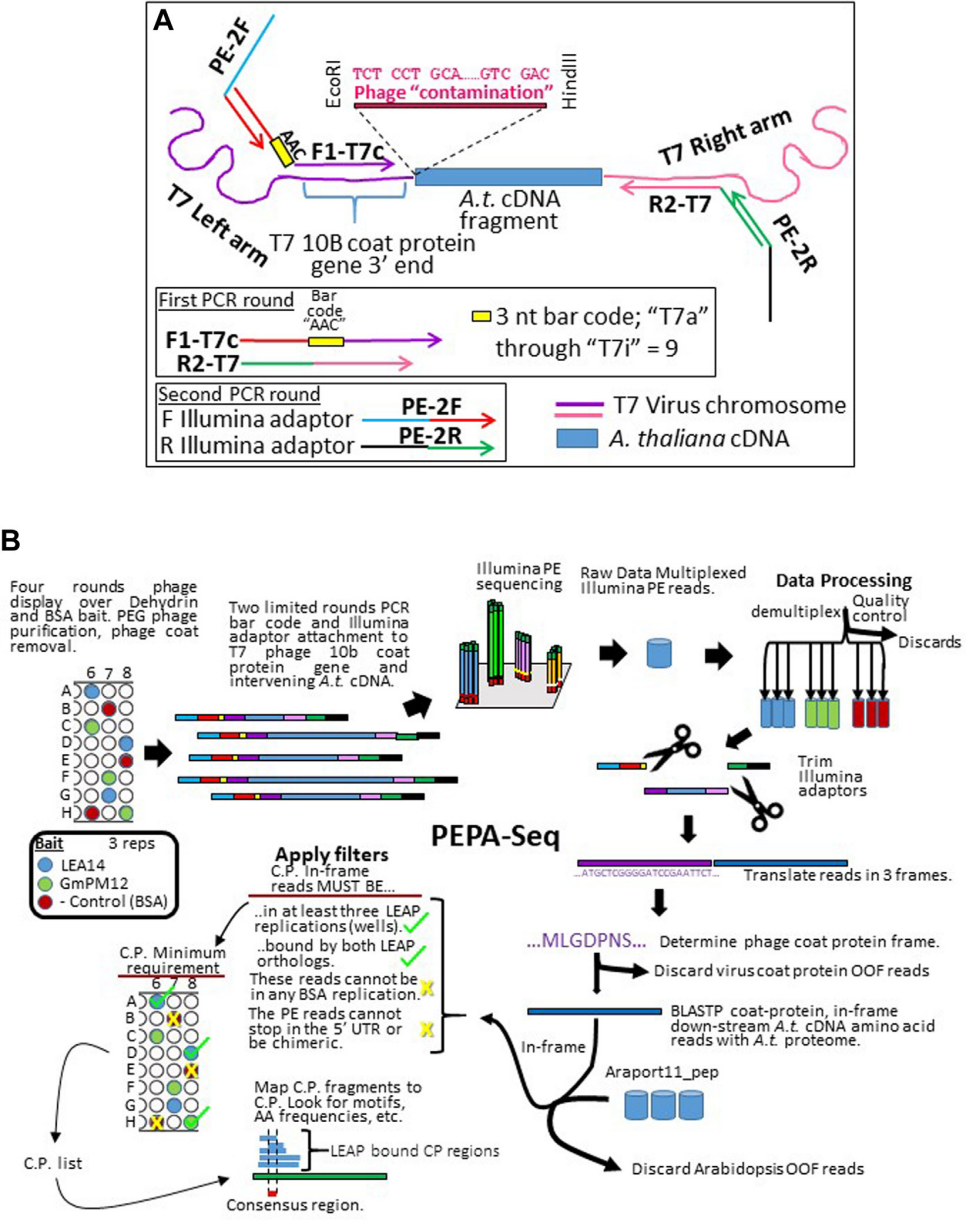
**PCR steps involved to place a three-nucleotide bar code and Illumina adaptors on either end of cDNA inserted into the T7 phage chromosome in preparation for paired-end phage sequencing.***A*, primers F1-T7- (*red, yellow box, purple arrow*) corresponding to the T7 10B coat protein 3′ end, 5′ to the cloning site (including the microtiter plate well–specific bar code and half of the forward Illumina adaptor; F Illumina adaptor; *red*) and R2-T7 (the reverse primer to the T7 10B coat protein 3′ end, 3′ to the cloning site) are used in a limited round PCR reaction. Template is the amalgam of T7 phage DNA extracted from the collection of clones from the terminal round of affinity selection over a specific LEAP and a specific microtiter plate well (replication). Following agarose gel electrophoresis (Supplemental Fig. S1), excision of the smear of amplicons above the primer dimers (if any), and DNA recovery, a second limited round PCR is performed. The second round uses primers PE-2F (*light blue, red arrow*) with PE-2R (*black*, *green arrow*) to complete the forward and reverse Illumina adaptors. *B*, all steps in the paired-end phage sequencing (PEPA-Seq) workflow. The width of the arrows in each step depicts the volume of reads passed from one step to the next. OOF, out of frame; LEAP, late embryogenesis abundant protein.

Aliquots of the PEG-purified phage were subjected to a two-step, reduced-cycle PCR protocol to introduce Illumina adaptor sequences onto Arabidopsis cDNA fragments present in the recombinant phage genome. Primers (T7SelectUp (F1-T7c in Fig. 3*A*) and T7SelectDOWN (R2-T7 in Fig. 3*A*); Supplemental Table S1) were designed to flank the 3′ region of the coat protein into which Arabidopsis seed cDNAs had been directionally cloned (Chen, Nayak *et al*. 2010). Phage sequences were flanked with Illumina-specific adapters (Supplemental Table S1; F1-T7-); in addition, three nucleotide bar codes were built into the T7SelectUP primer (yellow highlight in Supplemental Table S1; yellow box in Fig. 3*A*). Bar codes were unique to the phage recovered from each microtiter plate well (replication and bait protein) and were used with a universal reverse primer, R2-T7 (Fig. 3*A* and Supplemental Table S1). Following electrophoresis and gel purification ((29); Qiagen Inc) of the smear produced from this 12 cycle amplification (Supplemental Fig. S1), an aliquot was used as template for a second round of limited-cycle PCR that added the rest of the Illumina adaptors to the bar-coded cDNAs (Fig. 3*A* and Supplemental Table S1; primers PE-PCR1 and PE-PCR2). Following purification with AMPure beads (Beckmann Coulter), the tags were quantified (Qubit, Thermo Fisher Scientific), equimolar amounts were combined, and the mixture was submitted for paired-end MiSeq runs (2 × 250) at the University of Kentucky Health Care Genomics Core Laboratory (Fig. 3*B*).

### Translation of the R1 Reads and Selection of Those In-Frame with the Viral Coat Protein

The raw, bar-coded MiSeq reads were processed and further analyzed using the Qiagen CLC Genomics Workbench (CLC version 22.0; Qiagen). The three-nucleotide bar code on the forward read was used to demultiplex samples, yielding sets of paired-end reads for each phage preparation (Supplemental Table S2). R1 sequences were translated into the three forward frames using the portion of the T7 virus 10B coat protein situated 5′ of the insertion site of the Arabidopsis cDNA as a guide to define the reading frame (Supplemental Table S1 and Fig. 3, *A* and *B*). These translation products were scanned for the sequence “MLGDPN” that is part of the T7 10B coat protein and only protein sequences with this peptide were retained (Fig. 3*B*).

**Fig. 4:**
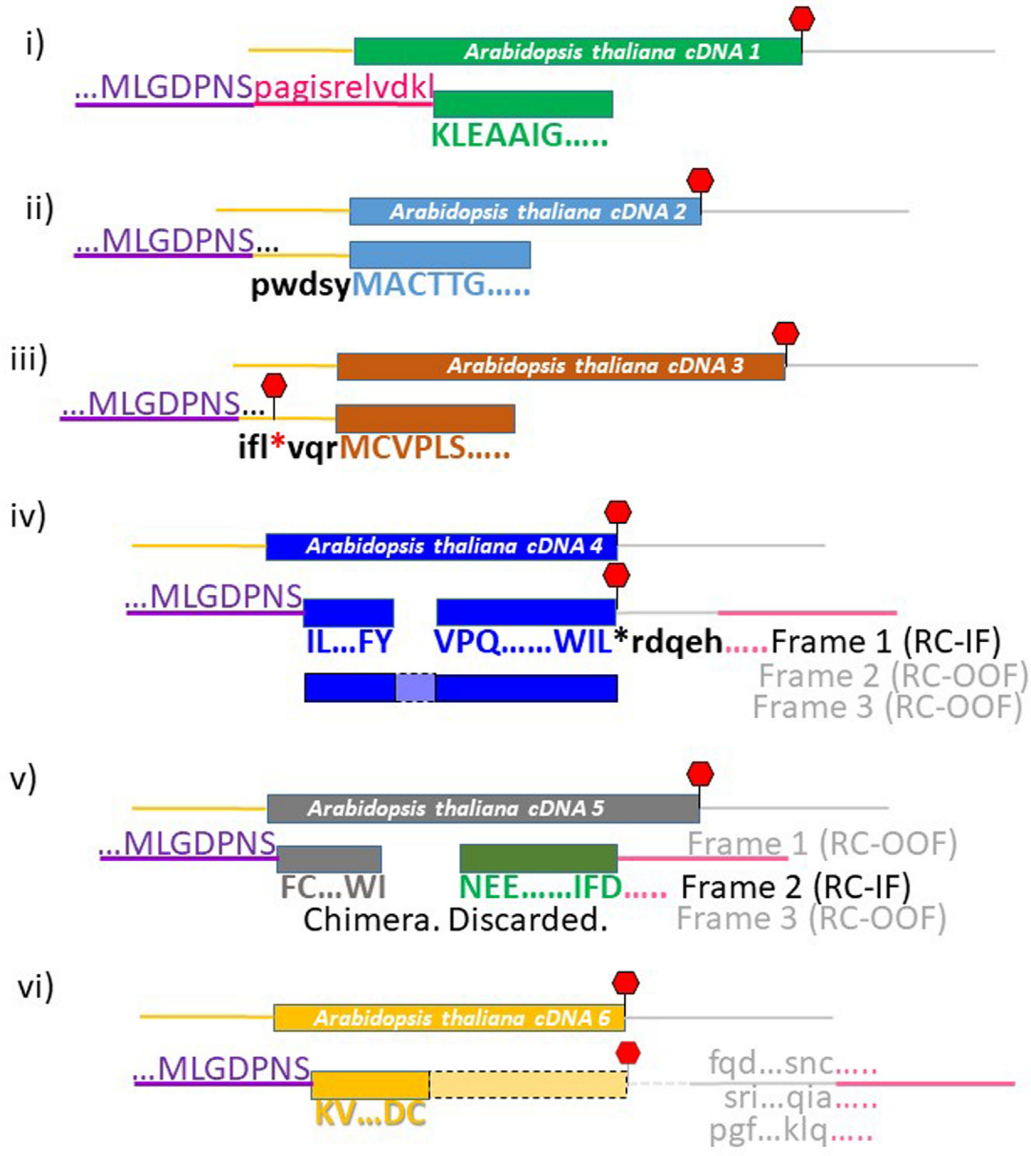
**Phage sequencing scenarios.***i*, occasionally, cDNAs could clone into the phage genome without using the adaptors, resulting in phage “contaminating” amino acid sequence (*red*). If this extra phage “leader” maintained the frame between the coat protein and the plant cDNA, the clone was retained as a legitimate, in-frame CtP. *ii* and *iii*, occasionally, the recovered clone commenced in the 5′ UTR (*bold black*) of the Arabidopsis cDNA (*yellow line*). These were examined in CLC Genomics Workbench to ascertain whether there was a stop codon in-frame between the end of the phage coat protein and the commencement of the Arabidopsis clone. If a stop was in-frame, these clones were discarded. *iv*-*vi*, occasionally, the reverse primer commences in the 3′ UTR of the Arabidopsis clone. *iv*, PEPA-Seq allowed clones longer than the two Illumina reads (*blue rectangles*) to be assembled based on the forward clone, in-frame with an Arabidopsis protein being identical with the protein identified from the reverse read, and the intervening protein sequence (*light blue rectangle*) was hence inferred. *v*, some clones identified one protein from the forward read and a second, unrelated protein from the reverse read (a long clone is depicted). These chimeric proteins were cloning anomalies that occurred during library construction. Which protein that was bound by the DHN was ambiguous and so these clones were discarded. *vi*, there were instances where the reverse read commenced in the 3′ UTR and terminated prior to reaching the coding region, and these were retained. CtP, client protein; DHN, dehydrin.

The frame encoding the virus coat protein dictates the reading frame of the protein displayed by the phage to which the Arabidopsis or soybean orthologous DHN or BSA had affinity. There were several cloning anomalies that could influence the legitimacy of Arabidopsis proteins fused to the coat protein (Fig. 4). A proportion of the Arabidopsis cDNAs inadvertently cloned into the viral genome without the aid of the adaptor that permits directional cloning, resulting in six possible frames downstream of the viral coat protein. Another instance of a cloning anomaly occurred when the Arabidopsis clone ligated into the HindIII site of the phage multicloning site, resulting in phage “contamination” of the resulting fusion protein (Fig. 4i). If the Arabidopsis sequence, fused downstream of the coat protein gene (with or without the aid of an adaptor), commenced in the 5′ UTR of a cDNA (Fig. 4, *ii* and *iii*) it is possible that a stop codon occurred prior to the commencement of the actual Arabidopsis protein. These hits were examined individually using the “Extract sequences to a new sequence list” function of CLC Genomics Workbench (CLC version 22.0; Qiagen) and translated in 3 forward frames. This established the virus coat protein frame and allowed determination of stop codons when the 5′ UTR was translated (Fig. 4*Aii*, *iii* and Supplemental Fig. S2, *A* and *D*). Sequences with stop codons in the 5′ UTR were discarded (Figs. 3*B* and 4*Aiii*).

At this stage, viral coat protein sequences were removed from the retained sequences and the remaining amino acid sequences compared with the Arabidopsis proteome using BLASTP, with the Arabidopsis proteome as the query and the translation products as the library (Fig. 3*B*). The Arabidopsis proteome was retrieved from Phytozome 12.1 (Araport11; (30)). Only paired-end reads in which both reads derived from the same gene were retained as legitimate CtPs (Fig. 4v and Supplemental Figs. S2–S4).

For those CtP hits where read1 (R1s) displayed a legitimate Arabidopsis protein fused to the virus coat protein, but were too long to result in a contiguous assembly with their R2 paired read, there were four possible scenarios. The first possibility is that the two translations could be anchored on the same CtP and the intervening sequence, encoding the amino acids between the reads, filled in (legitimate CtP; Fig. 4*iv* and Supplemental Fig. S4). Second, the R2 translation was to a different protein than the R1 read, in which case it was assumed that the hit resulted from a cloning chimera and was discarded (Fig. 4v). Third, the R2 translation was unrecognizable in the Arabidopsis proteome because it was only the 3′ UTR but of the same CtP identified by the forward read (Fig. 4*vi*); in which case it was retained. Last, the R2 translation was unrecognizable in the Arabidopsis proteome because all or part of it was 3′ UTR but to a different cDNA than the R1 read, resulting from a cloning chimera, and so was discarded (not shown).

When both reads possessed reading frames whose translations could be matched to the same protein, the entire protein sequence was used for subsequent analysis (Fig. 4*iv*). In cases where the R2 sequence seemed to be derived from the respective 3′-UTR, that portion of the protein sequence defined by the R1 read and the associated translation termination codon (inferred from the Arabidopsis annotation) was used for analysis (Fig. 4vi and Supplemental Fig. S4).

### Criteria Used to Screen for Legitimate DHN CtPs Using BSA as a Negative Control

The in-frame reads identifying Arabidopsis proteins from DHN and BSA affinity selection were compared and only those reads that were absent in the BSA wells were retained. A DHN CtP was considered legitimate if it was present in at least one well in both orthologous DHN groups, Arabidopsis LEA14 and GmPM12, and was present in at least three of the 6 target group (DHN) replications (wells; Fig. 3*B*). Following filtering, the resulting DHN CtP list was annotated using the Araport11 annotations ((Cheng, Krishnakumar *et al*. 2017); Table 1).

**Table 1:** Client proteins bound by both DHN proteins AT2G21490 and Glyma: 04G009900.1 (GmPM12)

GID	*At* Wells	*Gm* Wells	All *At*	All *Gm*	Ind. *At*	Ind. *Gm*	Function, if known
AT1G10320	2/3	2/3	16	55	2	2	ZINC FINGER C-X8-C-X5-C-X3-H TYPE family protein mRNA splicing, *via* spliceosome.
AT1G18540	2/3	1/3	5	2	2	1	RIBOSOMAL PROTEIN EL6Z, L6 family protein.
AT1G48130	2/3	1/3	33	4	2	1	1-CYSTEINE PEROXIREDOXIN 1 (ATPER1, PER1).
AT1G56070	3/3	1/3	32	12	3	2	LOW EXPRESSION OF OSMOTICALLY RESPONSIVE GENES 1 (LOS1). RIBOSOMAL PROTEIN S5/ELONGATION FACTOR G/III/V family protein.
AT1G63770	2/3	2/3	24	149	3	3	PUROMYCIN-SENSITIVE AMINOPEPTIDASE. PEPTIDASE M1 family protein. Natural antisense transcript overlaps with AT1G63770.
AT1G70320	3/3	2/3	32	25	4	3	UBIQUITIN-PROTEIN LIGASE 2 (UPL2). Ubiquitin-protein ligase-like protein containing a HECT domain.
AT1G80270	2/3	2/3	156	17	2	2	PENTATRICOPEPTIDE REPEAT 596 (PPR596). Mitochondrial and plastidal location.
AT2G21490	3/3	3/3	132	61	10	6	LEA14∗, DHN group LEAP. Self-binding. This was the DHN (and its soybean ortholog, GmPM12) over which the phage display library was affinity selected.
AT2G21870	2/3	3/3	4	56	2	**3**	MALE GAMETOPHYTE DEFECTIVE 1 (MGP1). PHOSPHITE-INSENSITIVE 1 (PHI1). Encodes the Fad subunit of mitochondrial F1F0-ATP synthase. Essential for pollen formation. Copper ion binding. Mitochondrial location?
AT2G41060	2/3	1/3	4	3	2	1	UBP1-ASSOCIATED PROTEIN 2B (UBA2B). RNA-binding (RRM/RBD/RNP motifs) family proteins. Regulation of programmed cell death.
AT2G42000	3/3	1/3	56	2	3	1	*Arabidopsis thaliana* METALLOTHIONEIN 4A (ATMT4A). Plant EC metallothionein-like protein, family 15. Involved in the accumulation of metal ions especially Zn in the seeds.
AT2G42650	2/3	1/3	19	2	2	1	RIBOSOMAL PROTEIN L1P/L10E family.
AT3G04120	2/3	1/3	5	2	2	1	GLYCERALDEHYDE-3-PHOSPHATE DEHYDROGENASE C SUBUNIT 1 (GAPC1). The mRNA is cell-to-cell mobile.
AT3G13290	1/3	2/3	2	203	1	**2**	VARICOSE-RELATED (VCR). Varicose-like protein.
AT3G16780	2/3	2/3	100	11	2	2	RIBOSOMAL PROTEIN–LIKE 19B (RPL19B). Ribosomal protein EL19Z (EL19Z).
AT3G19920	3/3	1/3	95	101	4	2	BROAD-COMPLEX, TRAMTRACK AND VIRUS AND ZINC FINGER (BTB/POZ) DOMAIN PROTEIN.
AT3G21140	3/3	1/3	76	3	4	1	ONEIRIC1 (ONE1). PYRIDOXAMINE 5-PHOSPHATE OXIDASE family protein.
AT3G23940	2/3	2/3	5	192	4	2	DIHYDROXYACID DEHYDRATASE (DHAD). A family of proteins that encode enzymes involved in branched chain amino acid biosynthesis.
AT3G24080	3/3	3/3	26	59	6	**8**	KRR1 family protein, ribosome assembly factor.
AT3G24650	1/3	3/3	2	35	1	**5**	ABSCISIC ACID INSENSITIVE 3 (ABI3). Homologous to the maize transcription factor viviparous 1 (VP1).
AT3G30775	2/3	3/3	5	203	2	**3**	PROLINE DEHYDROGENASE 1 (PDH1). EARLY RESPONSIVE TO DEHYDRATION 5 (ERD5).
AT3G48430	3/3	2/3	273	8	5	2	ETHYLENE INSENSITIVE 6 (EIN6). RELATIVE OF EARLY FLOWERING 6 (REF6). The mRNA is cell-to-cell mobile.
AT3G50240	2/3	1/3	75	2	2	1	KINESIN-RELATED PROTEIN, ATP-binding microtubule motor family protein, (KICP-02).
AT4G16210	2/3	1/3	8	5	2	1	ENOYL-COA HYDRATASE/ISOMERASE A (ECHIA). ENOYL-COA HYDRATASE2 (E-COAH-2).
AT4G19710	1/3	2/3	1	41	1	**2**	ASPARTATE KINASE-HOMOSERINE DEHYDROGENASE II (AK-HSDH II). plastidial location?
AT4G20850	1/3	2/3	20	4	1	**2**	TRIPEPTIDYL PEPTIDASE II (TPP2).
AT4G25630	3/3	2/3	16	4	3	2	FIBRILLARIN 2 (ATFIB2). The mRNA is cell-to-cell mobile.
AT4G27170	2/3	2/3	18	4	2	2	SEED STORAGE ALBUMIN 4 (SESA4).
AT4G32010	1/3	2/3	12	66	1	**2**	VIVIPAROUS1/ABI3-LIKE 2 (VAL2). I2-L1, HSI2-LIKE 1, HSL1, and HSI2-like 1. Transcriptional repressor involved in the recruitment of PRC2 for genome-wide polycomb silencing.
AT5G13870	2/3	1/3	152	1	2	1	XYLOGLUCAN ENDOTRANSGLUCOSYLASE (EXGT-A4, XTH5).
AT5G21970	2/3	1/3	5	8	2	1	UBIQUITIN CARBOXYL-TERMINAL HYDROLASE family protein.
AT5G39740	2/3	2/3	43	49	6	3	RIBOSOMAL PROTEIN L5 B (RPL5B).
AT5G46840	2/3	1/3	36	21	2	1	RNA-BINDING PROTEIN (RRM/RBD/RNP motifs) family protein.
AT5G56710	2/3	1/3	6	2	2	1	RIBOSOMAL PROTEIN L31E (EL31×) family protein.
AT5G62700	2/3	2/3	6	202	2	**3**	TUBULIN BETA CHAIN 3 (TUB3). The mRNA is cell-to-cell mobile.

In-frame tags identified thirty-five CtPs recovered from no fewer than 3 of the possible six wells containing the two orthologous LEAPs (*At* and *Gm* Wells). Additionally, no occurrence of these in-frame tags was recovered from any of the three BSA control wells. All *At*: The sum of all tags retrieved from the three Arabidopsis LEA14 wells. All *Gm*: The sum of all tags retrieved from the three soybean GmPM12 wells. Ind. *At*: Demonstrably independent clones retrieved from the three Arabidopsis LEA14 wells. Ind. *Gm*: Demonstrably independent clones retrieved from the three soybean GmPM12 wells. Instances where more independent clones to the CtP were obtained from soybean than Arabidopsis are provided as bold, underlined numbers. CtPs with a mitochondrial and/or plastidial localization are indicated. ∗The LEAP numbering/naming system is that of Hundertmark and Hincha (6).

BSA, bovine serum albumin; CtP, client protein; DHN, dehydrin; LEAP, late embryogenesis abundant protein.

### Mapping and Analysis of CtPs and LEA14 and GmPM12 bound Regions

The position of each of the tags was mapped on the full-length CtP sequence (Fig. 3*B*) and the entire Arabidopsis CtP was assessed for hydrophilicity and recognizable amino acid motifs (Supplemental Figs. S5–S39). Amino acid frequencies were acquired for the entire CtPs, the DHN-bound regions of the CtPs, and consensus fragments where the DHN-bound regions overlapped. These were compared with published amino acid frequencies for the entire Arabidopsis proteome from a variety of sources (31, 32, 33, 34, 35) (see Statistical Analysis section).

### DHN:CtP Interaction Assessment Using TRIC

LEA14 (AT2G21490) was one of the 35 phage-displayed CtPs retained after all filters had been applied (Table 1). Recombinant LEA14 and GmPM12 were buffer exchanged for a carbonate buffer (Zeta column, Thermo Fisher Scientific) and 0.5 ml of the proteins (10 μM) were separately fluorescently labeled using N-hydroxysuccinimide chemistry (Red-N-hydroxysuccinimide second Generation; Amine Reactive; NanoTemper Technologies). After removal of unreacted dye and quantification of the labeled proteins, the degree of labeling was ascertained using A_280_, A_650_, the DHN extinction coefficients, and formula provided in the labeling kit (Nanotemper Technologies). Unlabeled LEA14 or GmPM12 ligands, over a range of concentrations, were separately assessed for binding to labeled LEA14 or GmPM12 using TRIC assays with a Dianthus Pico instrument and DI.Control software (https://shop.nanotempertech.com/en/dianthus-software-package-1-license-91; NanoTemper Technologies). Each TRIC assay was repeated in triplicate and at least twice using independently produced recombinant protein. Resulting TRIC traces were assessed using DI.Screening Analysis 1.1.3 software to calculate the dissociation constant. Values from multiple, independent assays were exported to Excel, the change in Fnorm calculated, normalized among independent experiments, and used to generate representative figures for fraction bound ligand (SigmaPlot).

### Experimental Design and Statistical Rationale

The experimental design is outlined for the phage display and Paired-End PhAge Sequencing (PEPA-Seq) in Figure 3*B*.

### LEAP Phylogenetic Comparison/Statistics

All available LEAP protein sequences were retrieved from The Arabidopsis Information Resource (TAIR) or SoyBase databases (See the Supplemental Text for the finer details for the sequences included and excluded). These were first aligned in Clustal Omega (Sievers, Wilm *et al*. 2011) of the EMBL European Bioinformatics Institute (36). Sequences were uploaded to MEGA11 (37), and the evolutionary relationships were inferred using the maximum parsimony method (Fig. 1*A*). The maximum parsimony tree was obtained using the Subtree-Pruning-Regrafting algorithm (pg. 126, (38)) with search level 3 in which the initial trees were obtained by the random addition of sequences (10 replicates). There was a total of 903 positions in the final dataset of 131 amino acid sequences.

### CtP Bioinformatic Analysis/Statistics

The final list of CtPs was analyzed using The Gene Ontology Consortiums PANTHER16.0 (39) to establish significantly over/underexpressed gene ontologies that may be a common thread among the CtPs. The compositions of the entire CtP, the DHN-bound regions of the CtPs, and consensus DHN-bound CtP regions (Fig. 3*B*) were analyzed for deviations from published amino acid frequencies from multiple estimations published on the Arabidopsis proteome (35); Bastien, Lespinats *et al*. 2004, (31, 32, 33). The Statistical Analysis System was used to calculate the Chi Square at alpha = 0.05 with 19 degrees of freedom (20 aa – 1; (40)) which was compared to the corresponding critical point (30.1; (41)). The number of positively charged, negatively charged, and all charged amino acids wer also compared among published frequencies and those of the CtP, the DHN-bound regions, and the consensus of these.

### BiFC Assays of Protein–Protein Interactions *In Planta*

Five putative CtPs (LEA14, SEED STORAGE ALBUMIN 4 [SESA4], PENTATRICOPEPTIDE REPEAT PROTEIN 596 [PPR596], RPL5A, and PAP12) were chosen to investigate protein–protein interactions *in planta*. The coding region of each CtP was amplified with primers eliminating the stop codon and adding BsaI sites to create unique, four base overhangs at each end (Supplemental Table S1) and GreenGate assembled into the pGGB000 Entry vector (42) using thermal optimal cycles for BsaI and T4 DNA ligase in NEBridge ligase master mix. Prospective positive clones were identified from BsaI digests of plasmid DNA that produced inserts of the anticipated size on 1% (w/v) agarose gels; these were confirmed using whole vector sequencing (Plasmidsaurus Inc). There were two modified pGGC000 vectors for fusing a portion of YFP to either the LEA14 DHN or the CtP. pGGC001 had 173 amino acids of the N-terminal YFP Venus (43) translationally fused to, and 3′ of, a 78 bp linker that was in frame with, and located 3′ to, the *CtP* sequence. The pGGC002 vector had 84 amino acids of the C-terminal *YFP Venus* (43) similarly located. In separate reactions, *LEA14 DHN* or the *CtP* coding sequences were fused to the linker followed by the N-terminal portion of YFP with assembly into the pGGM000 intermediate vector with an additional overnight ligation step after BsaI heat inactivation (42). Components were fused to yield the following order: the *pUBIQUITIN10* promoter (*pUBQ10*; pGGA006); either *LEA14 DHN* or the *CtP* (in their respective pGGB000 vectors); the linker-YFP-N- (pGGC001); followed by a linker sequence (Dummy D; pGGD002; (42)); the *UBQ10* transcriptional termination sequence and FH adapter (pGGE009 and pGGG001, respectively; (42)). Additionally, intermediate vectors were constructed in pGGN000 assembled in the following order with an additional overnight ligation after BsaI heat inactivation: H-A adaptor (pGGG002; (42)); *pCAULIFLOWER MOSAIC VIRUS 35S* (*pCaMV35S*; pGGA004); either *LEA14* or *CtP* (in their respective pGGB003 vectors); linker-*YFP*-C-portion of *YFP* (pGGC002); the linker sequence (Dummy D; pGGD002); *RIBULOSE-1,5-BISPHOSPHATE CARBOXYLASE SMALL SUBUNIT* (Rubisco; RBCS) transcriptional termination sequence (pGGE001); and the BASTA resistance marker (pGGF008). Both intermediate vectors (after sequencing; Plasmidsaurus Inc) were combined into pGGZ003 using the same GreenGate assembly method of thermal cycling. Potential positive clones were identified by fragment patterns following digestion (ScaI or HindIII depending on the insert) and were subsequently sequenced (Plasmidosarus Inc). Each binary vector containing Y- and -FP fusions with LEA14 and a CtP (or CtP and LEA14) was transformed together with the helper plasmid, pSOUP (44) into *Agrobacterium* strain GV3850 using freeze-thaw. Single colonies from the YEP plate containing 5 μg/ml tetracycline, 100 μg/ml spectinomycin, and 100 μg/ml rifamycin were grown overnight at 28 °C and 150 rpm. After centrifuging (6000*g*, 3 min, room temperature) and resuspension (in 0.9× per ml culture 10 mM MES, 10 mM magnesium sulfate, pH 5.5), Agrobacteria were infiltrated into several leaves of well hydrated, 1 h dark adapted, *Nicotiana benthamiana* from which leaf disks were recovered each day for up to 5 days and examined under a fluorescence microscope (Leica DM2500 LED) for YFP signal. A FV1200 laser scanning confocal microscope system (Olympus), equipped with a GaAsP detection filter, was utilized to detect Lifeact–Venus signal (excitation 515 nm). Images were acquired with a wet objective lens (Olympus 60×/1.20 W; UPlanSApo) with Z-planes (25–35 μm total, 1–4 μm each slice) using FV10-ASW v.4.2 software (https://www.olympus-lifescience.com/en/downloads/detail-iframe/?0%5bdownloads%5d%5bid%5d=847249651; Laser power, 3–4%; HV, 500–550; Gain, 1.25, and Kalman filter, 2). The *LEA14y*:*LEA14fp* vector was also stably transformed into *Arabidopsis thaliana* plants using floral dip (45) and examined for YFP signal. For CtPs predicted to have a subcellular localization in the nucleus, leaf disks (transient expression) or tissues (stable expression) were infiltrated 3 times, 5 min each time, under vacuum with 50 μM Hoechst’s stain 33342 (Thermo Fisher Scientific Inc.). In one instance where the CtP residence included chloroplasts, a laser setting of 488 nm was used to acquire chlorophyll autofluorescence by moving the emission bandpass window to 600 through 700 nm. Captured images were imported into ImageJ (Fiji; https://fiji.sc/) and scale bars added.

## Results

### Purification of DHNs from *E. coli*

The goal of this study was to assess the utility of high-throughput phage display for the identification of proteins that bind LEAPs. Two orthologous DHN proteins from Arabidopsis and soybean possessing conserved amino acid sequences YnSKn = Y3SK2 (Fig. 1*B*; (46, 47)) were chosen as baits, the rationale being that CtPs that bind both orthologs are likely to be authentic *in vivo* interaction partners of these proteins. Standard purifications typically used for other LEAPs (*e.g.*, purified from *E. coli* proteins using 20 min boiling) did not yield high-quality preparations (Fig. 2*A*). Accordingly, extracts from boiled *E. coli* cells were further fractionated using immobilized metal affinity chromatography utilizing carboxy-terminal hexahistidyl tags added in the course of cloning. Following elution and dialysis, these proteins had much improved purity (Fig. 2*A*) and were used for affinity selection. The apparent molecular mass on SDS-PAGE for both hexahistidyl-tagged DHNs (Fig. 2*A*) is considerably above that predicted using their amino acid sequence (Fig. 1*B*), a frequently observed attribute of LEAPs (48).

### Affinity Selection of Phage Libraries Using Recombinant DHNs or BSA as Baits

A custom-made phage display library in which Arabidopsis-derived polypeptides are displayed on the surface of T7 phage (23) was used to identify polypeptides that bind to immobilized DHNs. The titers of the libraries increased in successive rounds of affinity selection regardless of the protein used as bait (Fig. 2*B*). At the end of four rounds of selection, aliquots for each of the three replications per bait protein (Supplemental Fig. S1) were processed for further DNA isolation and DNA sequencing using PEPA-Seq (Fig. 3, *A* and *B*). This processing included an initial PEG purification of phage, because this improved overall PCR performance prior to sequencing (Supplemental Fig. S1).

The Illumina MiSeq paired-end reads were binned according to bar code, translated, the virus coat protein frame identified, and reads with the intact coat-protein retained. These retained reads were mapped to the 70,380 proteins referenced in the Arabidopsis proteome (Supplemental Table S2). A minimum of 14.7% (GmPM12 well 2) of the reads from a specific well (replication) and a maximum of 44.4% (GmPM12 well 1) mapped to a legitimate Arabidopsis protein (Supplemental Table S2). Most of these mapped reads retained both paired-end reads (Supplemental Table S2). There were 135 Arabidopsis CtPs identified downstream of the T7 10B coat protein by both paired end R1 and R2 tags. All tags from 37 CtPs that also occurred in a BSA well were discarded. There were 63 of the remaining CtPs that failed to occur in at least 1 well of one DHN ortholog and 2 wells of the other ortholog that were also eliminated from further consideration. Once these filters were applied (see Materials and Methods; Fig. 3*B*), the final list of CtPs for both orthologous DHNs (AT2G21490 and GmPM12) consisted of 35 proteins (Table 1). The stringency of the filters makes this list a conservative, but robust, estimate of the DHN’s protein binding repertoire.

### DHN:CtP Binding Validation Using TRIC Assays

**Fig. 5:**
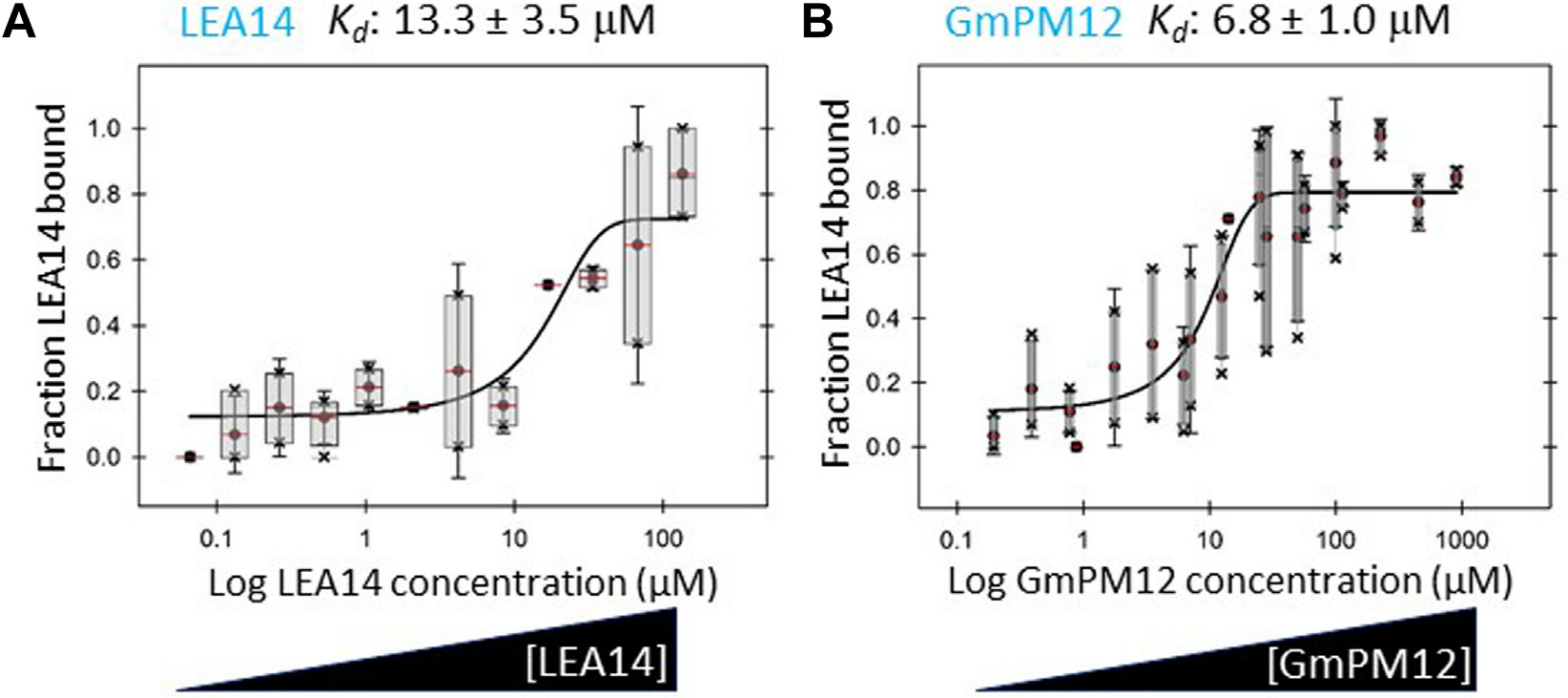
**TRIC assay of labeled LEA14 or GmPM12 binding to LEA14 or GmPM12, respectively.** The concentration of NHS-labeled DHN (either (*A*) LEA14 or (*B*) GmPM12) was kept constant while the concentration of the non-labeled binding partner (*A*) LEA14 or (*B*) GmPM12) was varied, decreasing incrementally by 2-fold. Immediately following centrifugation, the plates containing samples were loaded into a Dianthus Pico (NanoTemper Technologies) and temperature-related intensity change (TRIC) measurements performed using 3% power. Experiments were repeated at least 3 times and the average K_*d*_ ± standared error of the mean (SE) is reported. The normalized fluorescence curves from a typical experiment were converted to fraction-bound curves for presentation. DHN, dehydrin; NHS, N-hydroxysuccinimide.

Both LEA14 and GmPM12 were found to bind to the phage displayed LEA14 (At2G21490; Table 1). Using a Dianthus Pico, Cy5-labeled LEA14 or GmPM12 were used as targets with multiple 2-fold dilutions of unlabeled LEA14 or GmPM12, respectively. Both orthologous DHNs were found to self-associate verifying the association discovered by phage display (Fig. 5, *A* and *B*).

**Fig. 6:**
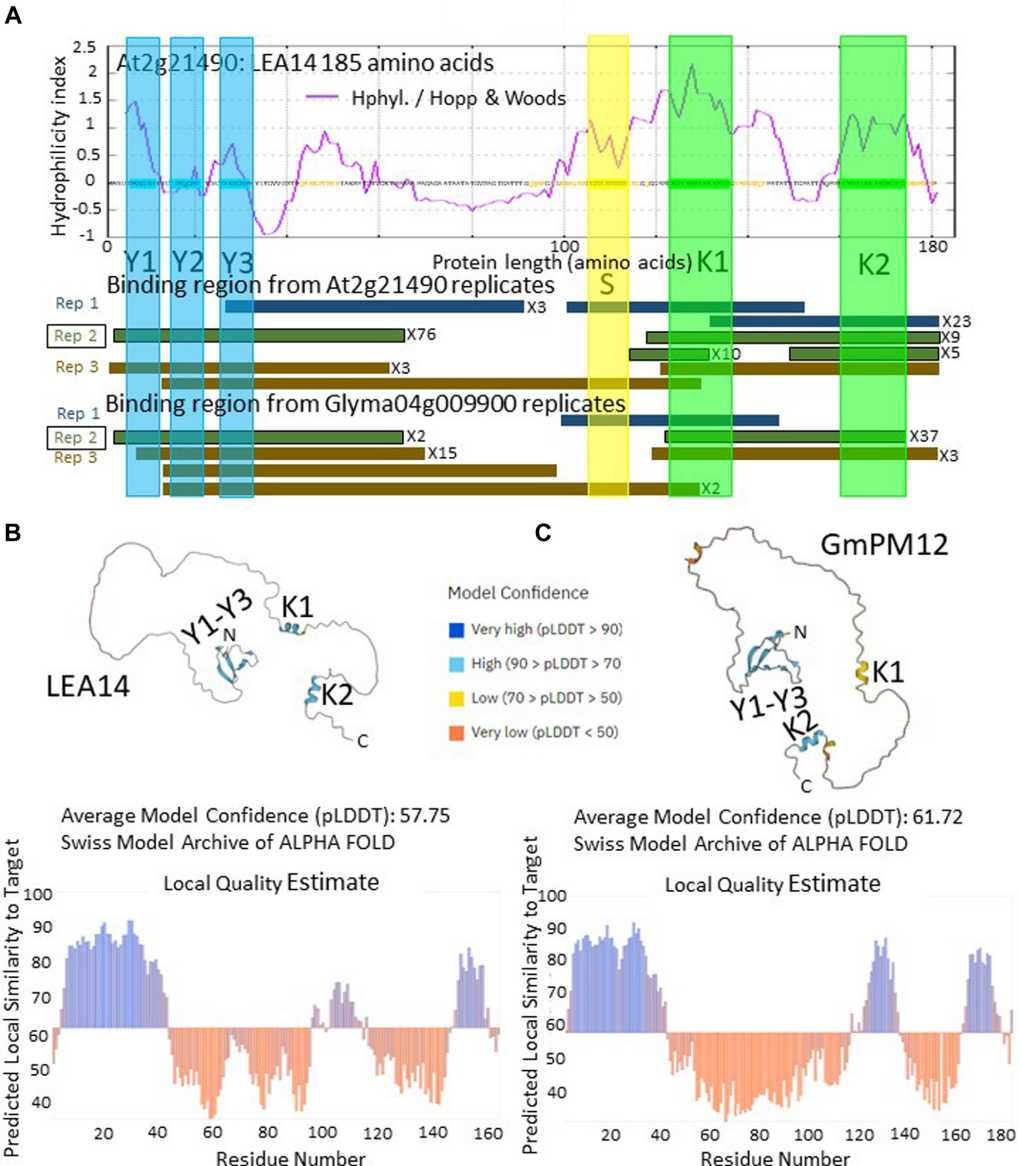
**The LEA14 DHN can self-associate and also bind with its soybean orthologous DHN.***A*, LEA14 phage-displayed fragments that bound either GmPM12 or LEA4 are depicted as *colored rectangles* on the stylized protein portrayal and project below the figure to the rectangular depictions of the independent phage displayed CtP fragments. The bound, phage-displayed regions recovered in each of the three independent wells (replications) for LEA14 or GmPM12 are presented separately below the overlaid Hopp-Woods hydrophilicity plot for each CtP. All LEA14 fragments, whether recovered from either LEA14 or GmPM12, contained at least one partial Y-domain or one partial K-domain. *B*, predicted LEA14 protein topology (AlphaFold) resolved only three regions where localized amino acid alignments, in relation to nearest neighbors, could be predicted with confidence of 79% or greater (*blue* demarcated sequences in the model labeled Y1-Y3, K1, and K2). These corresponded to an N-terminal region encompassing the three Y domains (*boxed regions highlighted in blue*) and the two K-segments toward the carboxy terminus (*regions highlighted in green*). *C*, this was also the case for Y1-Y3, and the K2 domain of GmPM12 while the K1 domain was predicted with only a 55 to 61% confidence. CtP, client protein; DHN, dehydrin.

The capacity of LEA14 and GmPM12 to bind LEA14 fragments during phage display demonstrated a tendency to bind to the DHN amino terminus containing the 3 Y segments (a conserved motif usually containing a tyrosine) and the carboxy terminus containing the 2 K segments (another DHN motif named due to a high lysine content), with a decline in tags situated in the center of LEA14, including the S segment (a serine rich segment; Fig. 6*A*; (49)). AlphaFold predictions indicated that the Y and K segments were the only regions of either LEA14 or GmPM12 likely to acquire structure in solution with high probability (Fig. 6, *B* and *C* (50, 51, 52);.

### CtP Characterization

The 35 CtPs were submitted to The Gene Ontology Consortiums PANTHER16.0 (https://pantherdb.org/) (39) to assess if there were commonalities among the CtPs for various biological process, molecular function, or cellular components relative to their overrepresented or underrepresented categories. The DHN’s CtPs were statistically significantly overrepresented in molecular function and cellular components. Regarding the molecular function of the CtPs, the category overrepresented was “binding” (27/35) primarily to “organic cyclic compounds” (20/35) which were chiefly nucleic acids (17/35), mostly RNA (14/35) and predominantly mRNA ((10/35); Supplemental Table S3). There were three statistically significantly overrepresented categories of cellular components. The first was protein-containing complexes (14/35), many of which were ribonucleoproteins (9/35) associated with preribosomes (3/35), predominately those of the 90S preribosome (2/35; Supplemental Table S4). The second was nonmembrane bounded organelles (16/35) and the cytosol (13/35) wherein (6/35) were members of the ribosome, mainly the large subunit (5/35; Supplemental Table S4). The last cellular component category overrepresented in the CtPs included those associated with a membrane-enclosed lumen (8/35) predominantly the nuclear lumen (8/35), specifically the nucleolus (7/35; Supplemental Table S4).

### Regions of the CtPs to Which the DHNs Bound

There were 28 CtPs where the DHNs bound to at least one identifiable domain or motif. The remaining seven CtPs possessed motifs/domains but the DHNs did not bind to the region of the CtP containing the motif, binding elsewhere in the protein instead. For example, while the B3 domain of ABSCISIC ACID INSENSITIVE 3 (ABI3) was bound (Supplemental Fig. S24), the interaction of the related protein VIVIPAROUS1/ABI3-like 2 (VAL2; Supplemental Fig. S33) involved a part of VAL2 that did not include the B3 domain despite both transcription factors belonging to the LEC2/ABI3/VP1/ABI3-like (LAV) group of B3-domain superfamilies (53). There were five genes encoding multiple possible protein isoforms only some of which possessed DHN-binding domains, as indicated in the phage display results (AT1G63770 [PEPTIDASE M1 family protein], AT3G19920 [BTB/POZ domain protein], AT4G19710 [ASPARTATE KINASE-HOMOSERINE DEHYDROGENASE II], AT5G46840 [RNA-BINDING (RRM/RBD/RNP motifs) family protein], and AT5G56710 [RIBOSOMAL PROTEIN L31E family protein]; Supplemental Figs. S9, S20, S28, S27 and S38; Supplemental Information). The best example is PEPTIDASE M1 family protein where, of the five independently captured PEPTIDASE M1 family protein tags, only 1 is present in all 7 variants. The 4 other tags identified were to the carboxy terminus; variants 3 through 7 are identical for the region identified as bound by the DHN homologs. Variant 2 would have only 15 aa different in the 322 aa domain; but variant 1 would be expected to have an entirely different amino acid composition at that carboxyl portion of the domain. Intriguingly, though still a part of the same domain, the soybean DHN did not bind to the more N-terminal portion of the same domain in all variants, unlike the Arabidopsis DHN.

The amino acid sequence of each of the 35 CtPs was retrieved from (TAIR (Phoenix Bioinformatics) and biochemical attributes obtained from submissions to Pfam (54) and Expasy (55, 56), including a Hopp-Woods hydrophilicity plot (57). Overlaying the DHN-binding sites on the CtPs, no consistent protein attribute provoking DHN binding was discernable (Supplemental Tables S3 and S4 and Supplemental Figs. S5–S39).

**Fig. 7:**
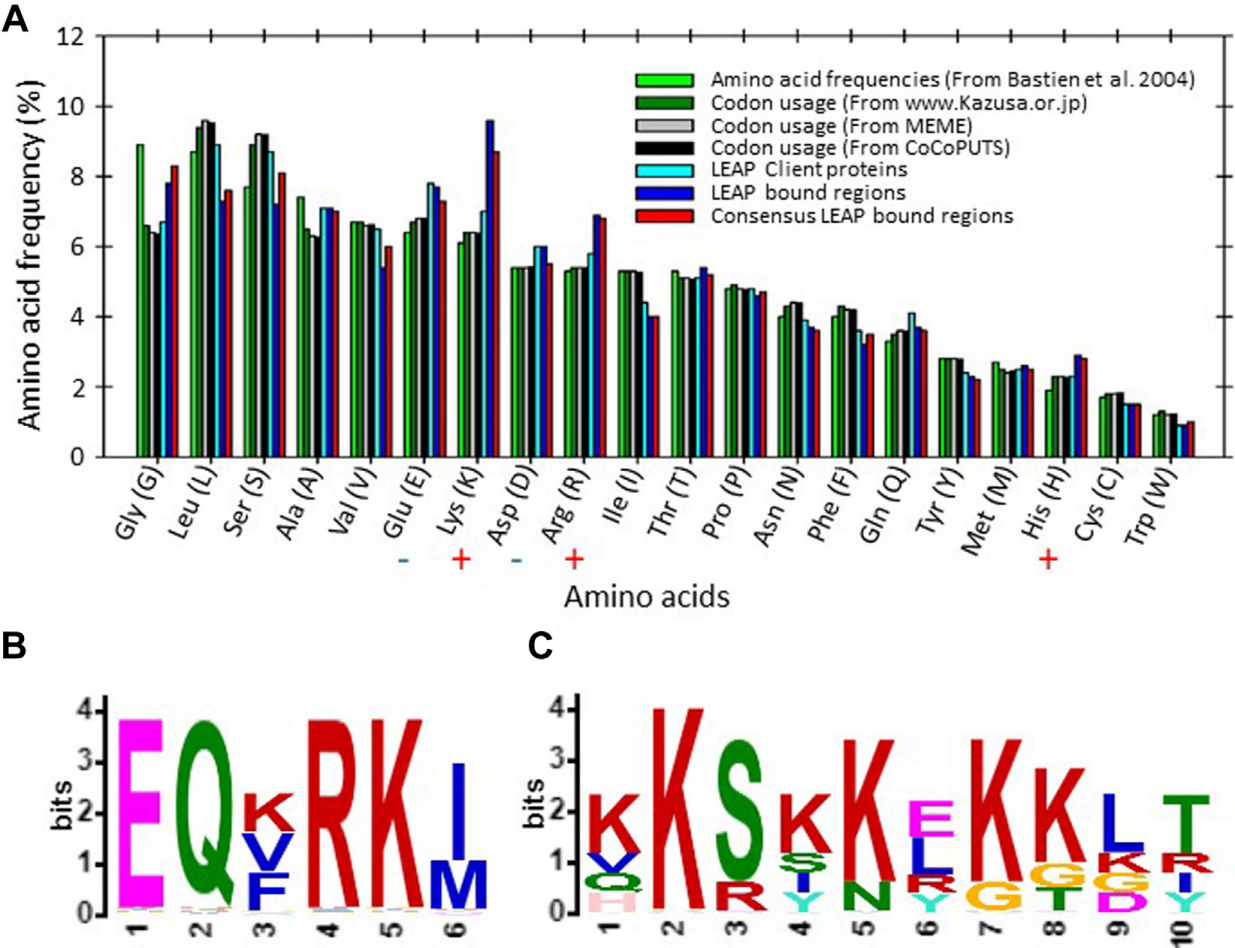
**Estimates of the amino acid frequencies in *Arabidopsis thaliana* proteins were acquired and compared to the amino acid frequencies present in the DHN CtPs, in the phage-displayed fragments of these CtPs, and the consensus sequences from the CtPs (**Fig. 3*B***).***A*, although the phage-displayed fragments captured by the orthologous DHNs tended to have a greater frequency of positively charged amino acids (110) than the CtPs themselves or the proteome generally, chi-square tests (critical chi-square value for 20 amino acids, 19 df: 30.1 = χ^2^ at *p* = 0.05) did not demonstrate that this was statistically significant. *B* and *C*, CtP sequences were used to acquire motifs present among them using XTREAM and the resulting WEBLOGOs (Crooks, Hon *et al*. 2004) were converted to peptide patterns and the Arabidopsis proteome scanned for proteins possessing the motif. There were five proteins discovered possessing each of the motifs and there were (*B*) three and (*C*) four of these captured in the phage display. CtP, client protein; DHN, dehydrin.

To further determine CtP characteristics in the regions bound by the DHNs, the CtP regions identified by the tags were concatenated (each tag was used once regardless of the number of times it was acquired), as was the entire set of CtP sequences. The amino acid frequencies for these concatenated CtP- and DHN-bound CtP regions were acquired using ProtParam (56) and compared to the amino acid frequencies in Arabidopsis (34), published codon usage among coding sequences (Kazusa), frequencies acquired from MEME (33), and those using Codon and Codon-Pair Usage Tables (CoCoPUTS; (Athey, Alexaki *et al*. 2017, Alexaki, Kames *et al*. 2019)) using Chi-Square analysis. There were no significant deviations in amino acid frequencies between the proteome and the CtPs. The trend for DHN bound CtP regions (whether restricted to consensus regions (Fig. 3*B*) or not) to contain positively (Lys, Arg, His), negatively (Glu, Asn), or any charged amino acids was not statistically significant (Fig. 7*A*).

To assess amino acid frequencies differently, the two estimates of proteome-wide amino acid frequencies were treated as replications as were amino acid frequencies for each of the 35 CtPs and the DHN-bound CtP regions. The General Linear Model (GLM) was used to test for significant differences in amino acid frequencies among the average for the proteome, the CtPs, and the DHN-bound CtP regions. There were no significant differences in amino acid frequency. Additionally, the CtPs were divided into various subgroups pertaining to function (*e.g.*, ribosomal proteins; hydrolases/hydratase) and the amino acid frequencies reassessed using the GLM. Again, no significant differences were observed.

The possibility that there were motifs of identical or similar amino acids in the CtPs, or DHN-bound CtP regions was examined using Clustal Omega (Sievers, Wilm *et al*. 2011 (58)) alignments and also the ProScan program (59), but these did not unveil any such motifs. Next, the DHN-bound CtP fragments were submitted either as concatenated- or single CtP sequences to the motif discovery tool, XSTREME (version 5.5.4), at the MEME Suite 5.5.4 site. Fragments were analyzed for motifs anywhere in the sequence using either the PROSITE fixed-length motifs (PROSITE 2021_04) or the Eukaryotic Linear Motif (ELM 2018) options for inputs of known motifs. In all four searches, there were two enriched motifs (Fig. 7, *B* and *C*) provided from the STREME subprocess with “rank 1” according to the simple enrichment analysis motif enrichment analysis program and with an E-value of 1.25e^-002^.

Given the DHN-bound fragments were encoded in a phage library from a normalized input of cDNA from Arabidopsis quiescent and germinating seeds, the logos (Crooks, Hon *et al*. 2004) (60) returned from STREME were converted to peptide patterns using the syntax provided by PatMatch (version 1.1; (61)) to search TAIR 10 and Araport 11 for proteins with these sequences. For the first motif (EQ[KVF]RK[IM]), the results from both databases were collapsed to 5 proteins by excluding isoforms. Three of these proteins were amongst those that were bound by the DHNs in this study. Those were AT3G24080 [KRR1 family protein], AT4G16210 [ENOYL-COA HYDRATASE 2], and AT4G27170 [SESA 4] (Supplemental Figs. S23, S28, and S32, respectively). The other two proteins containing this motif were AT1G16800 [P-LOOP CONTAINING NUCLEOSIDE TRIPHOSPHATE HYDROLASES SUPERFAMILY PROTEIN] and AT5G24314 [PLASTID-ENCODED RNA POLYMERASE-ASSOCIATED PROTEIN 12].

For the second motif ([KVQH]K[SR][KSIY][KN][ELRY][KG][KGT][LKGD][TRIY]), the PatMatch results with both databases returned 5 proteins (again collapsed by removing isoforms). Four of those five were captured by the DHNs from the phage library, namely AT2G42650 [RIBOSOMAL PROTEIN LARGE SUBUNIT 1P/L10E FAMILY], AT4G20850 [TRIPEPTIDYL PEPTIDASE II], AT5G39740 [RIBOSOMAL PROTEIN LARGE SUBUNIT 5B], and AT5G46840 [RNA-BINDING (RRM/RBD/RNP MOTIFS) family protein] (Supplemental Figs. S16, S30, S36, and S37, respectively). The fifth sequence encoded by AT3G25520 [RPL5A] represented a protein which differs in sequence from RPL5B by less than 2%.

### *In Planta* BiFC Assays for LEA14-CtP Association

**Fig. 8:**
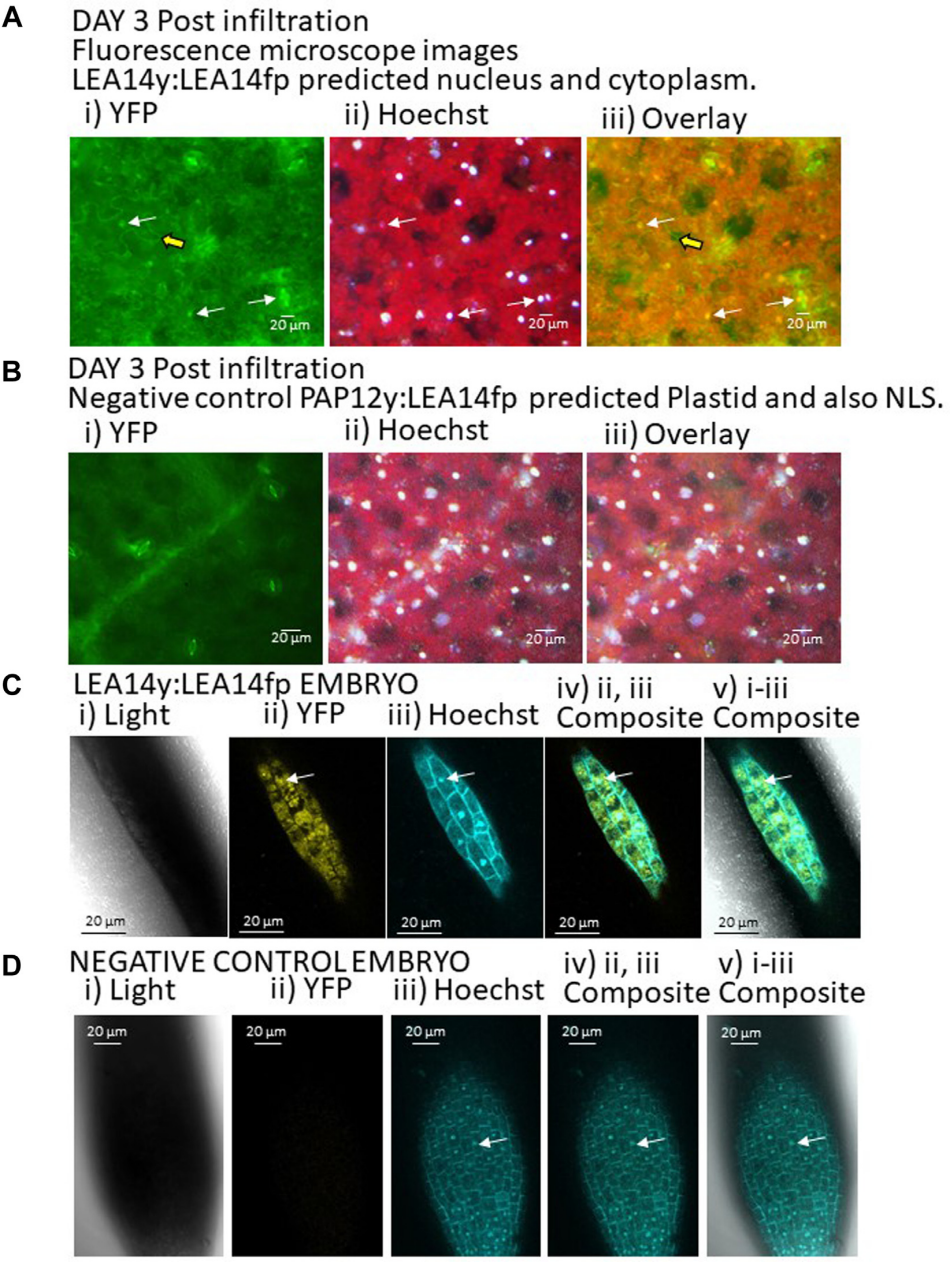
**The BiFC assay, using both transient expression and stable transformation, with split YFP was used to assess LEA14–CtP interactions for five CtPs.***A*, transiently expressed *LEA14y*:*LEA14fp* (LATE EMBRYOGENESIS ABUNDANT PROTEIN 14, dehydrin, At2G21490) constructs resulted in a cytoplasmic- and nuclear-localized YFP signal in *Nicotiana benthamiana* leaves (results from 3 days following infiltration shown). Such a signal was not observed in *N*. *benthamiana* leaves (depicted are *leaf disks* 3 days following infiltration) with, (*B*) *PAP12y*:*LEA14fp* or *LEA14y*:*PAP12fp* (not shown) constructs. Embryos of *Arabidopsis thaliana* which were stably expressing *LEA14y*:*LEA14fp*, (*C*) also demonstrated a cytoplasmic- and nuclear-localized YFP signal whereas those from untransformed plants (*D*) did not. For both tobacco leaves and Arabidopsis embryos, tissues were vacuum infiltrated 3 times for 5 min each time with 50 μM Hoechst’s stain prior to visualization. The scale bar represents 20 μm. In *A* and *B*; *i)* signal from reconstituted split yellow fluorescence protein (YFP). *ii*, Hoechst stain signal in the nucleus. iii) overlaid image. In *C* and *D*; *i*, light that is bright field images. *ii*, reconstituted YFP signal. *iii*, Hoechst stain signal. *iv*, overlaid signal from YFP and Hoechst stain. *v*, overlaid signal from light, YFP, and Hoechst stain. *White arrow*: nucleus. *Yellow broad arrow*: cytoplasm. BiFC, bimolecular fluorescence complementation.

**Fig. 9:**
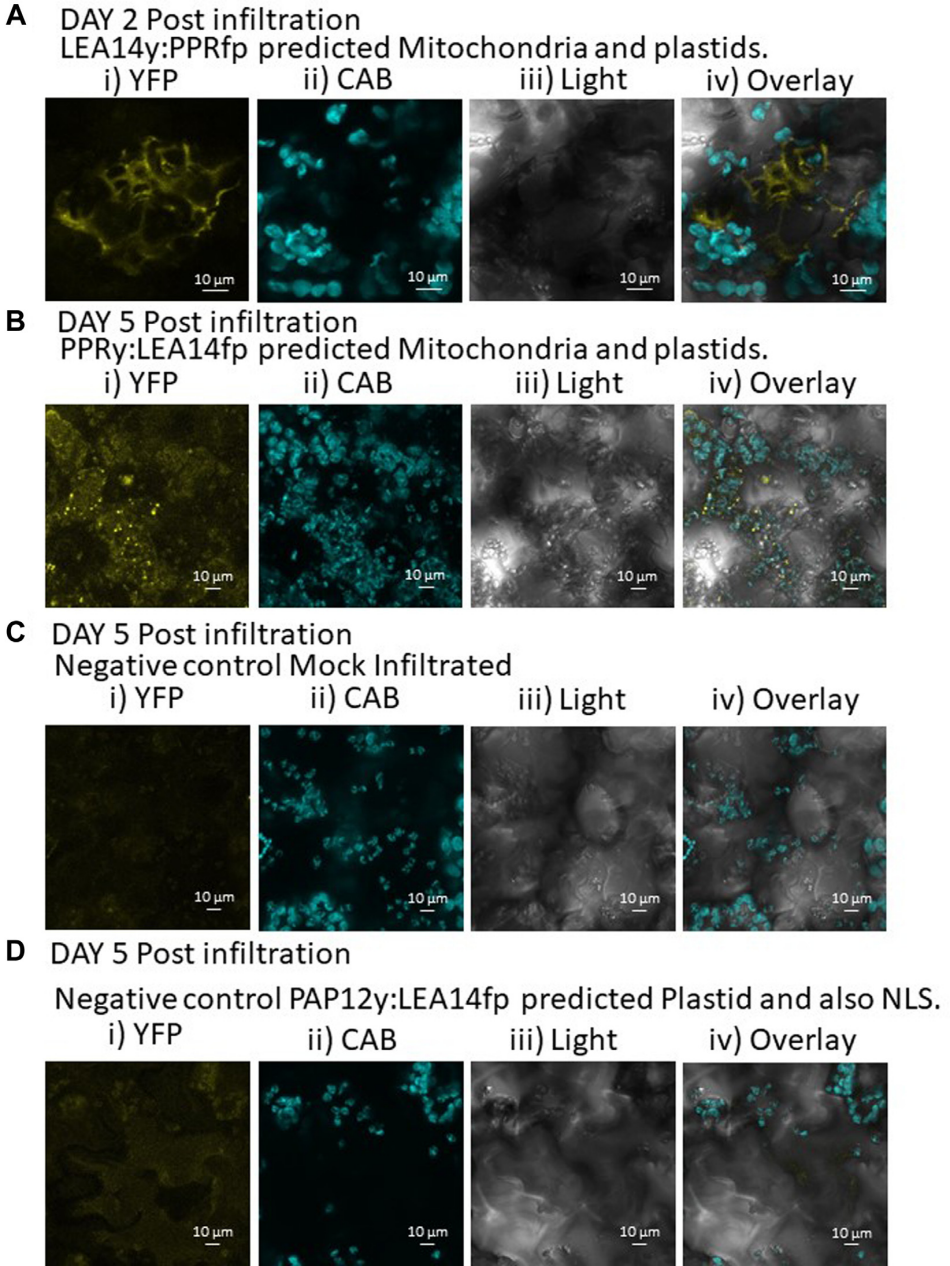
**Split YFP assay for PPRy:LEA14fp interactions.***A*, *LEA14y*:*PPRfp* (PENTATRICOPEPTIDE REPEAT 596, AT1G80270), resulted in YFP signal in *Nicotiana benthamiana* leaves (*leaf disks* 2 days following infiltration depicted) outside, sometimes proximal to, the chloroplasts. *B*, similarly, *PPRy*:*LEA14fp* constructs (*leaf disks* 5 days post infiltration presented) occurred in puncta that were beside but not in, chloroplasts. The YFP signal present in (*A* and *B*) was not observed in (*C*) mock-infiltrated or (*D*) leaves infiltrated with a negative control *PAP12y*:*LEA14fp* (leaf disks 5 days following infiltration depicted). *i*, YFP signal; ii, chlorophyll A and B autofluorescence (CAB; *cyan false color*); iii) light is brightfield micrographs; and iv) overlay of all three light signals. The scale bar represents 10 μm.

**Fig. 10:**
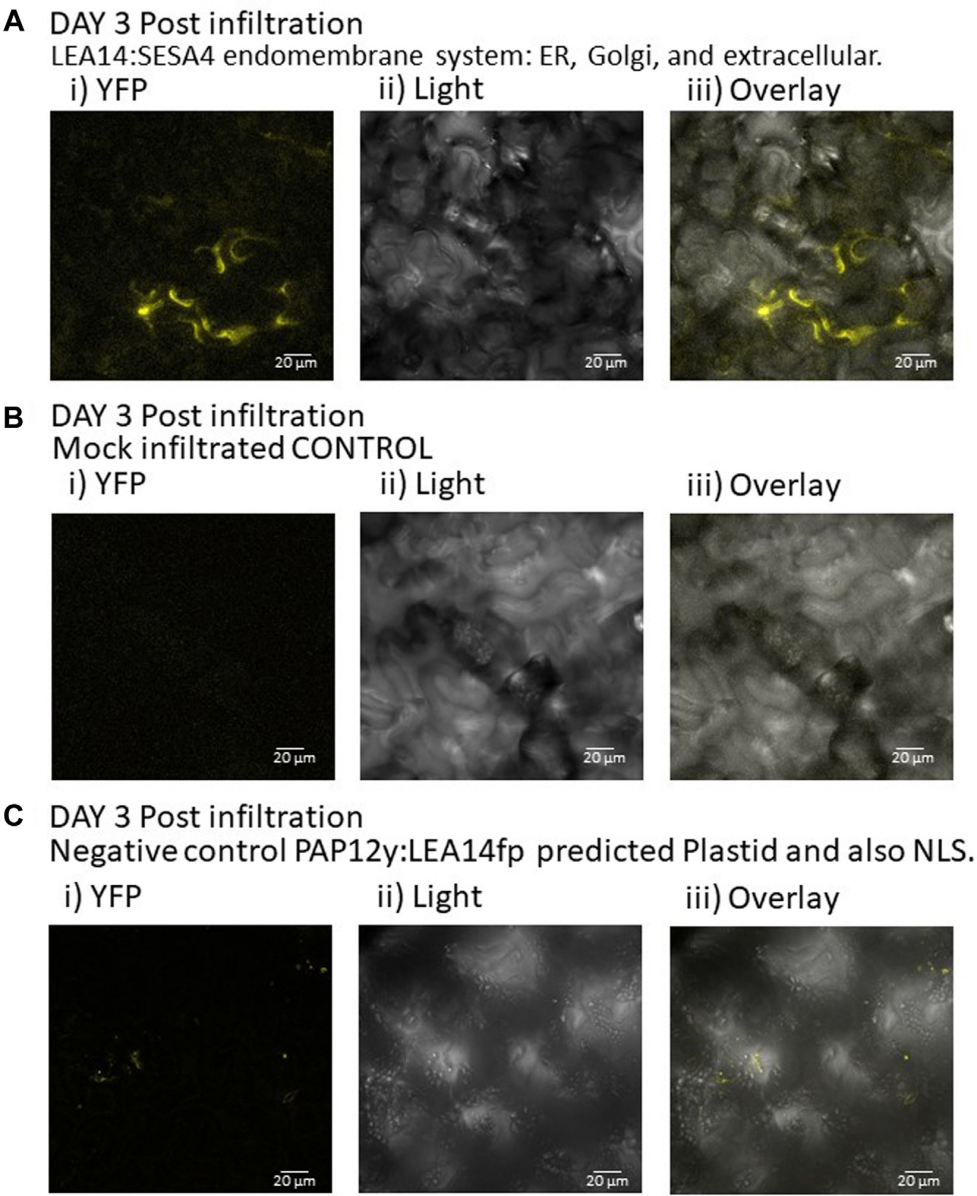
**Split YFP assay for LEA14:SES4A (AT4G27170: seed storage albumin 4) interactions.***A*, (*LEA14y*:*SESA4fp* (AT4G27170: SEED STORAGE ALBUMIN 4)) provided signal (3 days after infiltration shown) that is consistent with a localization in the endomembrane system, the published location of SESA4. i) YFP; ii) light; and iii) overlay. This signal was not evident in either, (*B*) mock-infiltrated or, (*C*) leaves infiltrated with the construct *PAP12y*:*LEA14fp* (images provided are 3 days after infiltration). The scale bar represents 20 μm. *i*, signal from reconstituted split yellow fluorescence protein (YFP). *ii*, bright field image (*light*). *iii*, overlaid image.

**Fig. 11:**
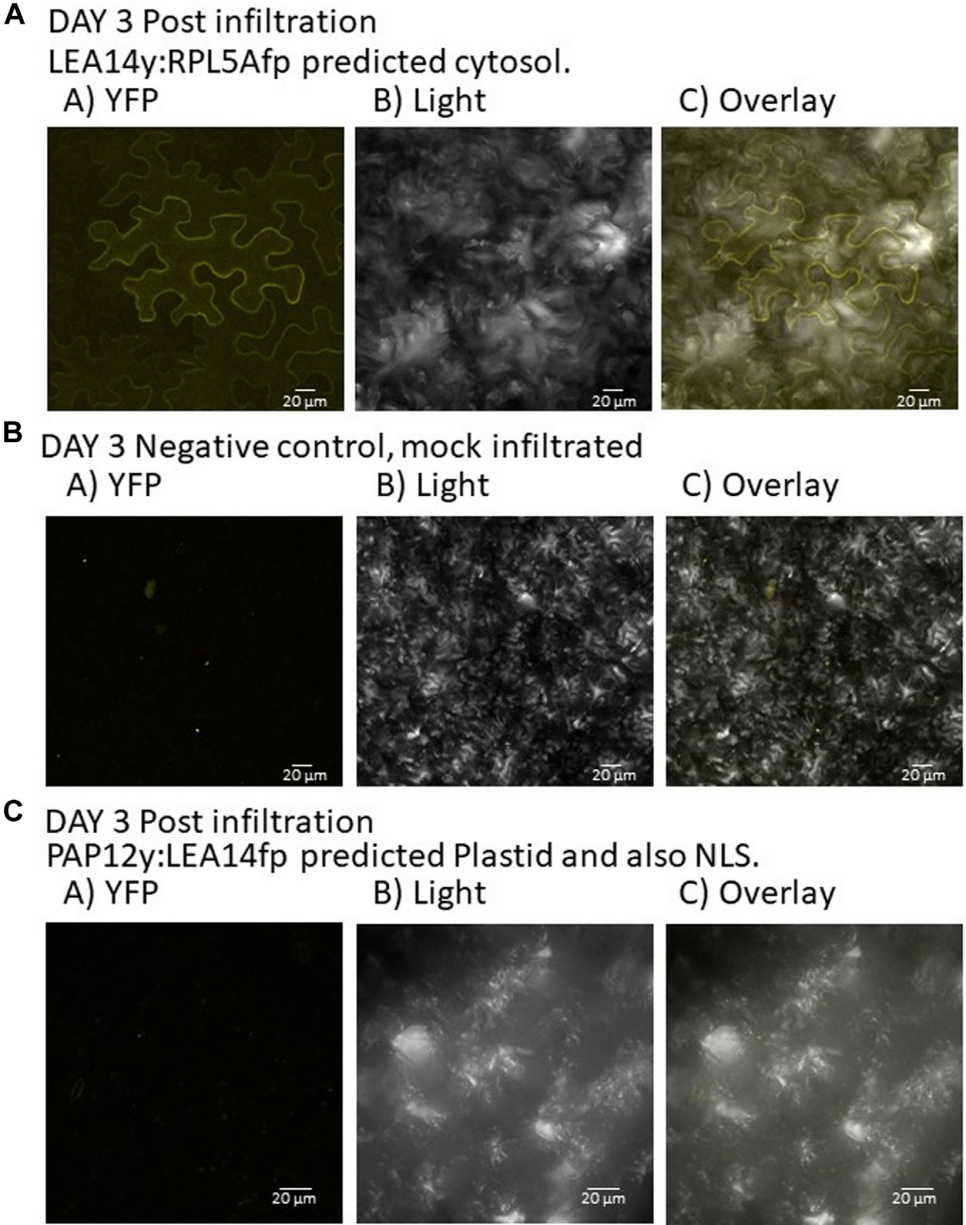
**Split YFP assay testing a CtP LEA14y:RPL5Afp (AT5G39740:RIBOSOMAL PROTEIN LARGE SUBUNIT 5A) that was not captured using phage display but shares an amino acid motif predicted from four other CtPs.***A*, YFP signal using *LEA14y*:*RPL5Afp* 3 days after infiltration that was present in the cytoplasm, a predicted subcellular localization for RPL5A. This signal was not evident in either, (*B*) mock-infiltrated or, (*C*) leaves infiltrated with the construct *PAP12y*:*LEA14fp*. The scale bar represents 20 μm. CtP, client protein.

A split YFP assay was used to test the interactions of five CtPs with LEA14. YFP fluorescence, as a result of BiFC from the split YFP fusions, from four of the five combinations of LEA14 and a CtP were visualized in (or around) a subcellular compartment consistent with the published localization of the CtPs (Fig. 8, Fig. 9, Fig. 10, Fig. 11). Of the five CtPs tested, the combination of LEA14:PAP12 (regardless of which YFP moiety was attached to the C terminus of the two proteins) was the single combination failing to result in observable YFP signal in tobacco leaf disks. This combination was subsequently used as a negative control for the other four positive interactions. LEA14y:LEA14fp signal was found in the cytoplasm and nucleus using transient leaf expression (Fig. 8*A*) and in the embryo using stable expression (Fig. 8*C*). LEA14 has previously been observed to exist in these locations (62). This signal was not observed in *LEA14*:*PAP12* infiltrated leaves (Fig. 8*B*) or in untransformed embryos (Fig. 8*D*). The PPR596 has been detected in mitochondria, plastids, and the plasma membrane (63, 64, 65). YFP signal from association of PPR596 with LEA14 was localized in cells, sometimes around, but not in, chloroplasts (Fig. 9*A*). Occasionally, the association providing YFP signal occurred in punctate loci (Fig. 9*B*) when both proteins were transiently overexpressed in tobacco leaf cells (Fig. 9, *A* and *B*). These puncta were not observed in mock infiltrated leaf disks (Fig. 9*C*) or in those from *LEA14*:*PAP12* split YFP infiltrations (Fig. 9*D*). The SESA4 is hypothesized to be localized to the endomembrane system in addition to being excreted to the apoplast (64). Signal from split YFP experiments with LEA14 and SESA4 was observed in amorphous sheets (Fig. 10*A*) similar in appearance to the tonoplast (66) from which storage protein vacuoles in dicot seeds are derived (67). These were not observed in mock infiltrated leaves or those infiltrated with LEA14:PAP12 split YFP constructs (Fig. 10, *B* and *C*, respectively). The predicted LEA14 CtP RPL5A, containing a motif discovered in four CtPs identified in this study, including its close paralog, RPL5B, was observed interacting with the DHN in the cytoplasm (Fig. 11*A*). No YFP signal was seen in mock infiltrated leaves (Fig. 11*B*) or in those infiltrated with *PAP12y*:*LEA14fp* (Fig. 11*C*).

## Discussion

Most phage display deep sequencing efforts have sought to identify unique peptides that robustly bind select entities in a variety of experimental contexts (68, 69, 70, 71).Libraries of displayed peptides (7–21 amino acids) may not be optimal for many studies, such as the identification of LEAP CtPs whose binding properties potentially involve larger domains. Displayed cDNA fragments, in contrast, are well suited for the identification of relevant interacting domains. The computational pipeline described in this report is sculpted by the features of displayed cDNA fragments. Thus, there is only one biologically relevant frame which is further limited to a single organism (Arabidopsis). Hence, the criteria of what constitutes a legitimate CtP for each LEAP is quite stringent and requires a hypothetical coding region to be in-frame with the upstream phage coat protein. This is not trivial, since many cDNA fragments may commence in the 5′ UTR. Our intent was to map the entirety of the CtP fragments recovered by each LEAP during affinity selection. Paired-end sequencing allowed both the amino- and carboxy-terminus of each tag to be effectively mapped on the relevant CtP. This provided: (1) a better representation of possible motifs and domains provoking LEAP binding and; (2) indications of either what CtP domains require protection, or which are targeted for interference, a recently unmasked consequence of some LEAP:CtP binding events (reviewed in (13)).

The list of proteins that were considered true CtPs was further curtailed by imposition of additional criteria. Thus, an in-frame fragment of each CtP must be bound by both orthologous DHNs (LEA14 and GmPM12), and each CtP must be found in at least three of the six target group replications, Arabidopsis LEA14 and GmPM12. In addition, tags were ruled out if the identical tag was found in any of the three wells constituting the BSA replications. This latter criterion may result in elimination of authentic CtPs, as it is conceivable that many DHN CtPs will have relaxed interaction properties. However, inclusion of this filter maximizes the probability that CtPs are authentic partners of Arabidopsis LEA14 and GmPM12.

A binding molecular function (organic cyclic compound binding) was significantly over-represented among CtPs based on gene ontologies. This functionality reflects the observation that several CtPs were part of a ribosomal complex. Protein protective LEAPs are an intrinsic element of the cellular natural protection and repair mechanism (72, 73, 74). Therefore, LEAP association with various proteins comprising the translational apparatus would be expected for reasons outlined previously (Job’s rule; (13, 23, 75, 76)).

The subcellular locations of the CtPs (or at least the cellular component with which the CtP is associated; Supplemental Table S4) were generally consistent with the published cytosolic and nuclear location of LEA14 (62). Even those CtPs associated with an organelle, when queried using The Cell eFP viewer of the Bio-Analytic Resource for Plant Biology ((BAR; (64)), have some probability of coexisting spatially with the reported nuclear/cytoplasmic residence of the DHN (62). There were three exceptions to this: specifically the CtPs AT1G80270 PPR596, AT3G30775 [PROLINE OXIDASE], and AT4G19710 [ASPARTATE KINASE-HOMOSERINE DEHYDROGENASE II] were listed as strictly localized to the chloroplast and/or mitochondrion (Table 1). PPR596 is also predicted to exist in/on the plasma membrane (64). However, PPR596 has been reported to interact with AT3G27960 [KINESIN LIGHT CHAIN-RELATED 2], AT5G03240 [POLYUBIQUITIN 3], and AT5G26210 [ALFIN-LIKE 4] all of which have cytosolic and nuclear (among other) localizations (77, 78, 79). PROLINE OXIDASE has been experimentally demonstrated to interact with at least one cytoplasmic- and nuclear-localized protein, AT5G65430 [14-3-3 PROTEIN G-BOX FACTOR14 KAPPA] (80), while ASPARTATE KINASE-HOMOSERINE DEHYDROGENASE II was proposed by the Cell eFP (BAR) to interact with AT5G40760 [GLUCOSE-6-PHOSPHATE DEHYDROGENASE 6] in the cytoplasm (64). A YFP signal was observed when LEA14 and PPR596 were ectopically, and highly, expressed in tobacco leaves which was localized in cells, sometimes around chloroplasts, a location not previously documented for LEA14 (62) but these results were obtained from experiments where PPR596 was not also overexpressed.

The STRINGS (81) and GeneMania (82) protein lists were manually inspected for “experimental determined” and “physical interaction” with LEA14, respectively. The STRINGS network lists 54 interacting proteins with LEA14, 47 of these were identified through text mining, 33 of which were coexpressed and 2 of these were listed as experimentally determined interactors (AT1G09770; CELL DIVISION CYCLE 5 and AT5G65490; PROTEIN ECDYSONELESS HOMOLOG, SUPPRESSOR-LIKE PROTEIN). GeneMania lists 312 coexpressed and 6 colocalized LEA14 proteins. Five proteins were predicted interactors with LEA14, while 15 had shared protein domains (Supplemental Table S5). Though both of these lists had generated a multitude of LEA14 interactions *via* coexpression analysis and text mining (Supplemental Table S5), none were in common with the 35 experimentally determined LEA14 CtPs from this work. Nor was there a predicted self-association for this particular DHN. Thus, there was no corroborating evidence at the protein level from these prediction/coexpression networks for the phage-display-generated list of LEA14 interactions.

The identification of two discriminating amino acid motifs present in some of the CtPs was intriguing. Each of these motifs exists in five Arabidopsis proteins. For the first motif (EQ[KVF]RK[IM]), three of the five proteins were captured in the PEPA-Seq. According to the absolute values in the eFP Browser in the seed portion of the Klepikova Arabidopsis Atlas ((55, 83); quiescent seed or 1 day after imbibition only) or in the seed EFP Browser (84, 85); excluding the seedling portions), the expression from AT1G16800 (P-LOOP CONTAINING NUCLEOSIDE TRIPHOSPHATE HYDROLASES SUPERFAMILY PROTEIN) and AT5G24314 (PAP12) is low in seeds. Hence, the expression from the genes encoding the two proteins exhibiting this motif, but not present in the PEPA-Seq, was probably too low (as compared to the three that were bound by the DHNs) to be represented in the phage library, considering the time points used for RNA isolation for preparation of the phage library (23). However, when PAP12 (AT5G24314) was used in the split YFP BiFC assays with LEA14, no YFP signal was detected in leaves using transient expression.

The second motif, ([KVQH]K[SR][KSIY][KN][ELRY][KG][KGT][LKGD][TRIY]), was also present in only 5 proteins and four of the five were present in the PEPA-Seq data. Two of the proteins with this motif are paralogs (RPL5A and RPL5B). RPL5B was present in the PEPA-Seq data but RPL5A was not, yet both paralogs are apparently expressed in seeds at the stages used to create the library. The information for the eFP Browsers for the imbibed and quiescent seed stages come from microarray data (86, 87). *RPL5A* and *RPL5B* differ by less than 2%, raising the possibility that the microarrays may not adequately discriminate between these two transcripts and both are present in seeds. Alternatively, RPL5A is not expressed sufficiently in quiescent or germinating seeds to be captured using the phage display technique described here. Nevertheless, RPL5A did generate a cytoplasmically localized signal in split YFP BiFC assays with LEA14.

Both orthologous DHNs, LEA14 and GmPM12, recovered LEA14 fragments in the phage display screen and were confirmed to autoassociate in TRIC assays with similar dissociation constants (Fig. 5, *A* and *B*). There are several reports of DHN homo- and hetero-dimerization (88), the consequences of which can include restriction of homodimer subcellular localization (89) or the activation of stress protective capacities (90). LEA14 homodimers were observed *in planta* in both transiently and stably expressed BiFC assays but there was no alteration in the subcellular localization determined using split YFP relative to that observed for the single, YFP-labeled LEA14 (62).

Questions arise concerning LEAP self-association which would likely decrease their effective size (*i.e.*, the sum of their hydration radii as monomers would be greater than as dimers), limiting their capacity to act as molecular shields. Also, self-association is likely to prevent the LEAP interaction with CtPs. However, one mechanism to control with which CtPs LEAPs may bind, and under what cellular stresses this would occur, would be to have them, in non-stressful situations, self-associate at a kD above that of only their most vulnerable CtPs. This would ensure that only these CtPs are bound by the LEAP while preventing the LEAP binding to other proteins at kDs above that for LEAP self-association. This is, essentially a “safe-fail” system that, under non-stressful situations; (1) prevents LEAP promiscuous binding to many proteins based on the lower LEAP:LEAP kD, effectively rendering the LEAPs innocuous while at the same time; (2) decreasing their overall effective hydration radius, reducing their molecular shield capacity. Stressful alterations in the cellular milieu could quickly decrease the kD of LEAP/CtP pairs (*e.g.* destabilized CtP structure induces a fit of the LEAP) and/or increase the kD for homodimerization (theoretically releasing LEAPs from homodimers), enhancing molecular shield functionalities and/or boosting CtP binding capacities immediately.

The LEA14 fragments that were bound by the orthologous DHNs always possessed either a Y or a K segment (47, 91), strongly implicating these motifs in the capacity of the DHNs to dimerize, and perhaps to bind other CtPs. Some interacting LEA14 and GmPM12 segments possess only Y-segments (Fig. 6*A*), which suggests that the Y-segment is capable of protein interaction. While the K segment has been previously identified as essential for enzyme protection, this protection is thought to occur without direct binding (92, 93). Nevertheless, in the current instance, the K segment, in a variety of phage-displayed oligopeptide contexts, and in some instances without any other motif, is clearly retained by both orthologous DHNs, implicating the K segment (90) in direct protein binding to form DHN homo- or hetero-dimers (Fig. 6*A*). In a recent review, three Arabidopsis DHNs capable of auto association possessed SK, but not Y, domains (AT1G20440, AT1G20450, and AT1G54410), further implicating the K segment in DHN autoassociation (94). Notable by their absence in the current CtP list are any other Arabidopsis, seed-expressed, DHN fragments.

These data have determined what LEA14 fragments are binding to LEA14/GmPM12. What cannot be ascertained from the current data is to what the K- or Y- segments are binding. For both LEA14/GmPM12 K- and Y-segments, it will be interesting to ascertain if K-segments associate with K-segments, Y- with Y-segments, the opposite, or if either or both segments bind some other portion of LEA14/GmPM12, although this last scenario is improbable based on the phage-displayed fragments (Fig. 6*A*).

There are a number of DHN CtPs that have been identified by others that have increased our understanding of how these LEAPs exert influence over their CtPs, and through this interaction, how they influence stress resilience. In addition to protection, an Arabidopsis DHN, EARLY RESPONSE TO DEHYDRATION 14, has been demonstrated to bind to and upregulate the activity of, a GLUTATHIONE TRANSFERASE (PHI9) whenever cells experience oxidative stress (12). As previously mentioned, DHN homo- or hetero-dimerization influences DHN subcellular localization and (perhaps as a result) protective influence (88, 89, 90). DHNs that associate with kinase CtPs can be posttranslationally modified to change their membrane affinities, influencing membrane properties (16, 95). Others situate on the plasma membrane due to their affinity for the cytoplasmic portion of intrinsic membrane proteins, such as the AQUAPORIN PLASMA MEMBRANE INTRINSIC PROTEIN 2B which the DHNs are hypothesized to protect from denaturation when PLASMA MEMBRANE INTRINSIC PROTEIN 2B is exposed to sub-optimal temperatures (96). ACTIN is another CtP with which DHNs (At1g20440, COR47; At1g20450, ERD10) interact and ERD10, at least, stabilizes ACTIN polymers from chemical perturbations (97). In *Medicago tuncatula*, the ERD10 ortholog, MtCAS31 (Medtr6g084640), binds LEGHEMOGLOBIN and protects it from denaturation (98). This same DHN binds INDUCER OF CBF EXPRESSION 1 (ICE1, also known as SCREAM1) during periods of low water availability, interfering with ICE1 function, thereby reducing stomatal density in developing leaves under water stress (99). Additionally, MtCAS31 acts as an adaptor protein physically linking its cargo protein, the AQUAPORIN PLASMA MEMBRANE INTRINSIC PROTEIN, MtPIP2;7 with AUTOPHAGY-RELATED GENE 8 PROTEIN in phagophores, which leads to the destruction of the aquaporins (and MtCAS31) through selective autophagy. This, in turn, influences membrane water permeability and, through this, drought tolerance (100). The current study has added to the burgeoning list of CtPs to which DHNs are known to bind. Many of the CtPs are components of the translational machinery the protection of which is of particular import (75). Clarification of the physiological consequence of LEA14 binding to some of these CtPs is ongoing.

## Data Availability

The alignments provided in Supplemental Information to describe the splice variants which could be discriminated by the DHN-bound fragments were performed using Clustal Omega (83)). The biochemical attributes provided in Supplemental Figures were obtained from submissions to Pfam (39) and Expasy (56), including a Hopp-Woods hydrophilicity plot (57). In the Supplemental Figures, the descriptions of the complexity of determining what constitutes valid bound fragments used screenshots from the Qiagen CLC Genomic Workbench (CLC version 22.0; Qiagen). The phage display library is available through the Arabidopsis Biological Resource, The Ohio State University, (Accession CD4-85). MiSeq reads are deposited in the NCBI Sequence Read Archive under the BioProject Accession ID: PRJNA1073232. Websites referred to in the text are as follows: Alpha Fold: https://alphafold.ebi.ac.uk; Araport 11: Download function at TAIR [https://www.arabidopsis.org/download/] has the Araport 11 protein lists from 2022; Cell eFP viewer: https://bar.utoronto.ca/cell_efp/cgi-bin/cell_efp.cgi; Clustal Omega: https://www.ebi.ac.uk/Tools/msa/clustalo/; Expasy ProtParam Tool: https://web.expasy.org/protparam/; Expasy ProtScale: https://web.expasy.org/protscale/; Expasy ProScan: https://prosite.expasy.org/; Genemania: https://genemania.org/search/arabidopsis-thaliana/At2G21490; GeneOntology Network: pantherdb.org, PANTHER18.0: https://pantherdb.org; HELIQUEST: https://heliquest.ipmc.cnrs.fr/cgi-bin/ComputParams.py; InterProScan: https://www.ebi.ac.uk/interpro/about/interproscan/; InterPro: https://www.ebi.ac.uk/interpro/; MEGA11: https://megasoftware.net; PD Arabidopsis codon usage: https://www.kazusa.or.jp/codon/cgi-bin/showcodon. Cgi?species=3702, Phytozome 13: https://phytozome-nex.jgi.doe.gov/info/Athaliana_Araport11; SoyBase: https://www.soybase.org/dlpages; STRINGS: https://string-db.org/network/3702.AT2G21490; SWISS-MODEL Repository: https://swissmodel.expasy.org/repository; TAIR: https://www.arabidopsis.org; and XSTREME: https://meme-suite.org/meme/tools/xstreme.

## Supplemental data

This article contains supplemental data (6, 30, 37, 58, 101, 102, 103, 104, 105, 106, 107).

## Conflict of interest

The authors declare no competing interests.

## Supplementary Data

Supplemental Figures

Supplemental Tables1_4

Supplemental Table 5

Supplemental_TEXT_MASTER
