# Supplementary material for: Dehydrin Client Proteins Identified Using Phage Display Affinity Selected Libraries Processed With Paired-End Phage Sequencing
Source: Mol Cell Proteomics. 2024 Oct 21;23(12):100867. doi: 10.1016/j.mcpro.2024.100867 (PMC11612773; doi:10.1016/j.mcpro.2024.100867)
Supplement: Supplemental Figure Legends [file mmc1.docx]

**Supplemental Figures**

**Supplemental Figure 1:** PEP-Seq, step one, the first, limited-round PCR reaction result using T7Select10-3b vector-specific and unique “bar-code tagged” primers (F1-T7_; where _ is primer “a” through “i”; Supplemental Table 1) and the common reverse primer (R2-T7) for each replicate amplifying an aliquot of phage lysate as template. The template is a mixture of various length cDNAs from different LEA-retained phage. The primers contain half of the Illumina adaptor specific for each end of the tag (Supplemental Table 1; Fig. 2A). **A)** The PCR reaction was performed for each of three independent microtiter plate wells (replications), following 4 rounds of selection and amplification, containing the soybean dehydrin protein (*Glyma04g009900*), its Arabidopsis orthologue (*At2g21490.1*), or Bovine Serum Albumin (BSA) a random, unrelated control protein used in the phage selection process. **B)** Same as **A** above but each aliquot of phage lysate was PEG purified before amplification. MWM: Molecular Weight Marker (1 kb DNA Ladder).

PEP-Seq, step two, a second, limited-round PCR reaction result using aliquots of gel-purified amplicons from step one and common primers (PCR-1 and PCR-2) which places the second half of the Illumina adaptor sequence on the tags. **C)** DNA template from step one amplicons from phage DNA without PEG purification. **D)** DNA template from step one amplicons from phage DNA with PEG purification. MWM: Molecular Weight Marker (1 kb DNA Ladder).

**Supplemental Figure 2:** A clone aligning to a potential CP (At1g70600) but, **A)** originating in the 5’UTR, the consensus sequence terminates in, or just prior to, the At1g70600 start codon. This sequence was: **B)** extracted to ascertain if it is in frame with the virus coat protein. The relevant tag frame (in frame with the virus coat protein) is boxed horizontally in green. There is no stop codon between the virus coat protein and the At1g70600 start codon (vertically boxed in green). **C)** A clone aligning to a potential CP (At3g04920) but, **D)** originating in the 5’UTR, the consensus sequence (in frame with the virus coat protein; horizontally boxed in green) terminates at a stop codon situated in the 5’ UTR (boxed in red) before the CP start codon (vertically boxed in green).

**Supplemental Figure 3:** An example of mapping paired end reads (R1 & R2) sufficiently short that the limited read length (150 bp each way) permits overlap. The light yellow sequence is the viral vector of the 3’ end of the T7 viral coat protein 10B. The EcoRI site is boxed in black and the viral coat protein reading frame codons are boxed in purple with the encoded amino acids, in purple, above each depicting the carboxy-terminus of the viral coat protein. The transition to the Arabidopsis, LEAP-acquired protein (At2g43680), is in-frame with the virus coat. The identity of the protein encoded by the reverse read matches that of the forward read with all but one of the reads transitioning back to the virus genome at the HindIII site (black boxed sequence).

**Supplemental Figure 4:** An example of mapping paired end reads (R1 & R2) over a gap due to the limited read length (150 bp each way; At5G62690 shown; see Figure 4Biv). The light yellow sequence is the viral vector of the 3’ end of the T7 viral coat protein 10B. The EcoRI site is boxed in black and the viral coat protein reading frame codons are boxed in purple with the encoded amino acids, in purple, above each depicting the viral coat protein. The dark pink dashes indicate uncovered regions separating the forward and reverse reads. Note that the reverse read commences in the 3’UTR for all six reads and includes the stop codon (boxed in red). All of the reverse reads are to the same protein as the forward reads and so are retained. In this case the first (uppermost) frame is legitimate and the gap between the forward and reverse frames is filled in from the mapped read.

**Supplemental Figures 5-39: Biochemical attributes of the CP regions bound by the LEAPs.** All Client protein amino acid sequences were retrieved from the Arabidopsis Information Resource (TAIR), and protein biochemical properties analyzed and amino acid motifs sought using algorithms available in ProtScale (Expasy) and Pfam (EMBL). Hopp-Woods hydrophilicity plots are overlaid on the client proteins and site where tags, pertaining to the CPs, were bound by the LEAPs are identified. The number of times a particular tag (**_x** **[**within square brackets**]**) was recovered in the PEP-Seq and the amino acid sequence the tag represents, is provided for each LEAP (At: *Arabidopsis* *thaliana* LEA14; Gm: *Glycine max* GmPM12) replicate (R1-R3; microtiter plate well). If multiple protein isoforms are possible due to alternative mRNA processing, a description of the alteration is presented at the bottom of the figure in grey text (e.g. “9 aa deletion due to alternative splicing” in this figure).
