# Supplementary material for: Dehydrin Client Proteins Identified Using Phage Display Affinity Selected Libraries Processed With Paired-End Phage Sequencing
Source: Mol Cell Proteomics. 2024 Oct 21;23(12):100867. doi: 10.1016/j.mcpro.2024.100867 (PMC11612773; doi:10.1016/j.mcpro.2024.100867)
Supplement: Supplemental Figures [file mmc2.pptx]

## Slide 1
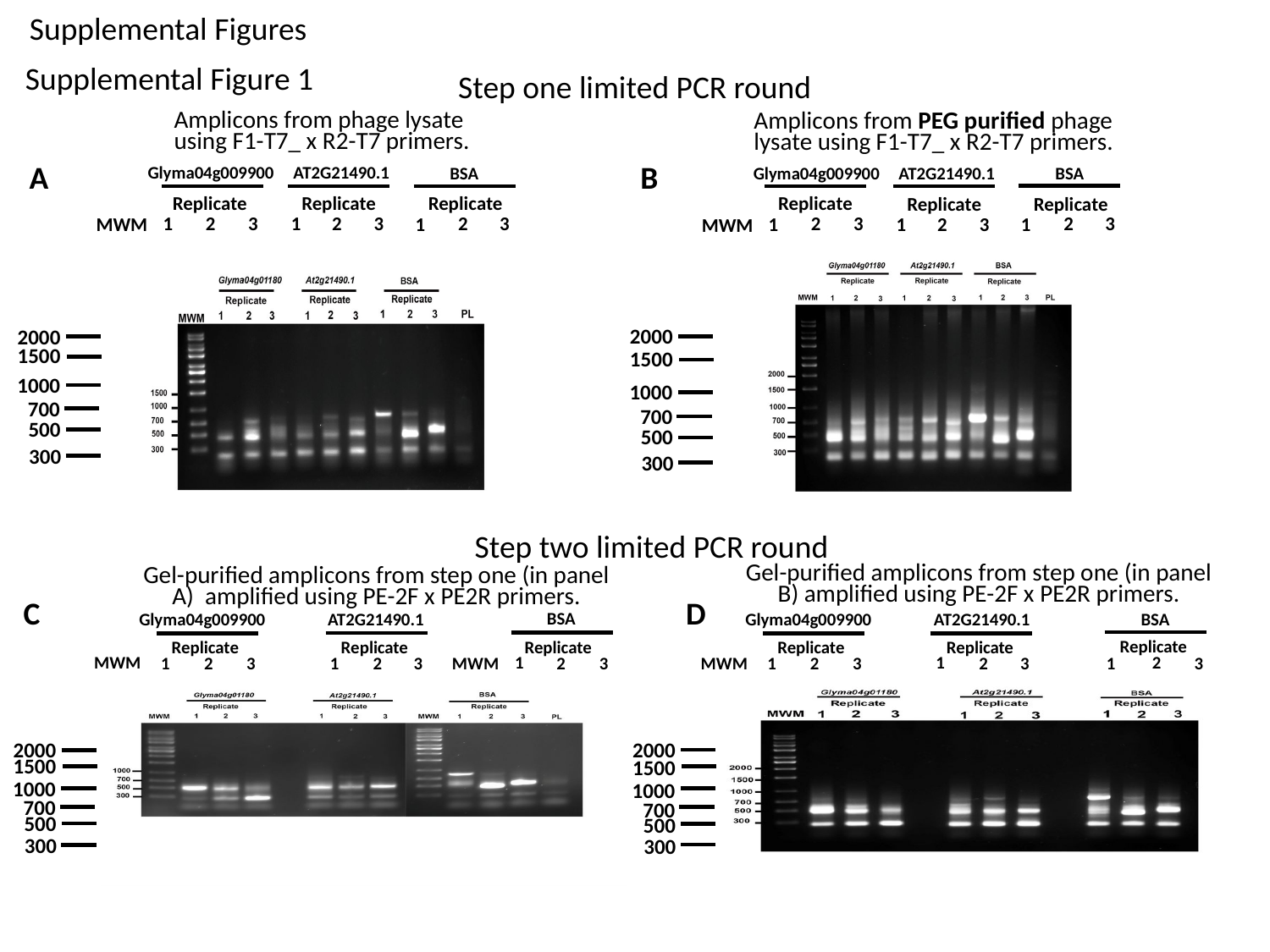

Supplemental Figures
Supplemental Figure 1
Step one limited PCR round
Amplicons from phage lysate using F1-T7_ x R2-T7 primers.
A
Glyma04g009900
AT2G21490.1
BSA
Replicate
Replicate
Replicate
2
3
2
3
1
2
3
1
1
MWM
2000
1500
1000
700
500
300
Amplicons from PEG purified phage lysate using F1-T7_ x R2-T7 primers.
B
2000
1500
1000
700
500
300
Glyma04g009900
AT2G21490.1
BSA
Replicate
Replicate
Replicate
2
3
2
3
1
2
3
1
1
MWM
Step two limited PCR round
Gel-purified amplicons from step one (in panel B) amplified using PE-2F x PE2R primers.
D
Glyma04g009900
Replicate
1
2
3
MWM
AT2G21490.1
Replicate
1
3
2
BSA
Replicate
2
3
1
2000
1500
1000
700
500
300
Gel-purified amplicons from step one (in panel A) amplified using PE-2F x PE2R primers.
C
BSA
Replicate
1
2
3
MWM
AT2G21490.1
Replicate
1
3
2
Glyma04g009900
Replicate
2
3
1
MWM
2000
1500
1000
700
500
300

## Slide 2
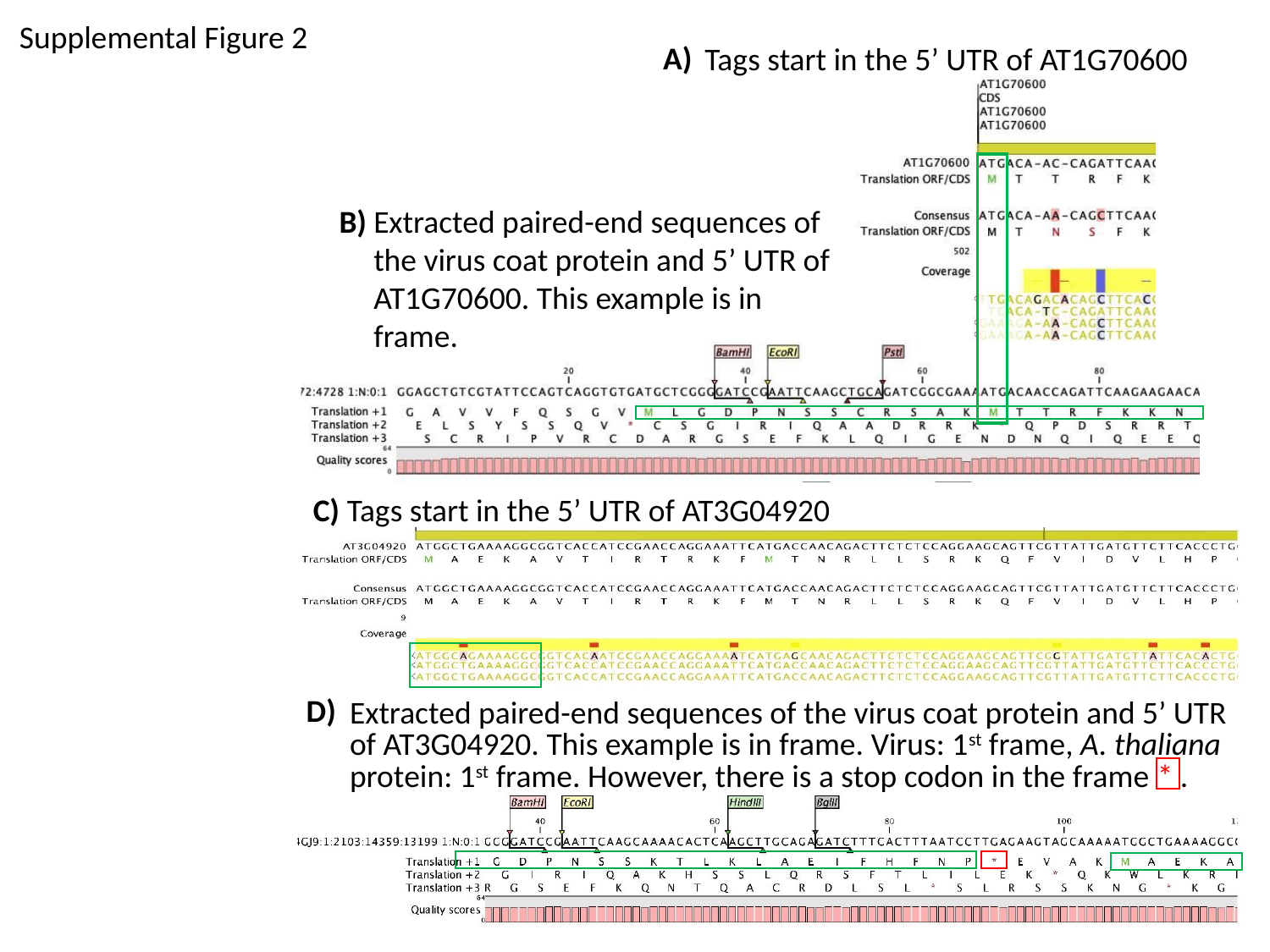

Supplemental Figure 2
A)
B)
Tags start in the 5’ UTR of AT1G70600
Extracted paired-end sequences of the virus coat protein and 5’ UTR of AT1G70600. This example is in frame.
C) Tags start in the 5’ UTR of AT3G04920
Extracted paired-end sequences of the virus coat protein and 5’ UTR of AT3G04920. This example is in frame. Virus: 1st frame, A. thaliana protein: 1st frame. However, there is a stop codon in the frame * .
D)

## Slide 3
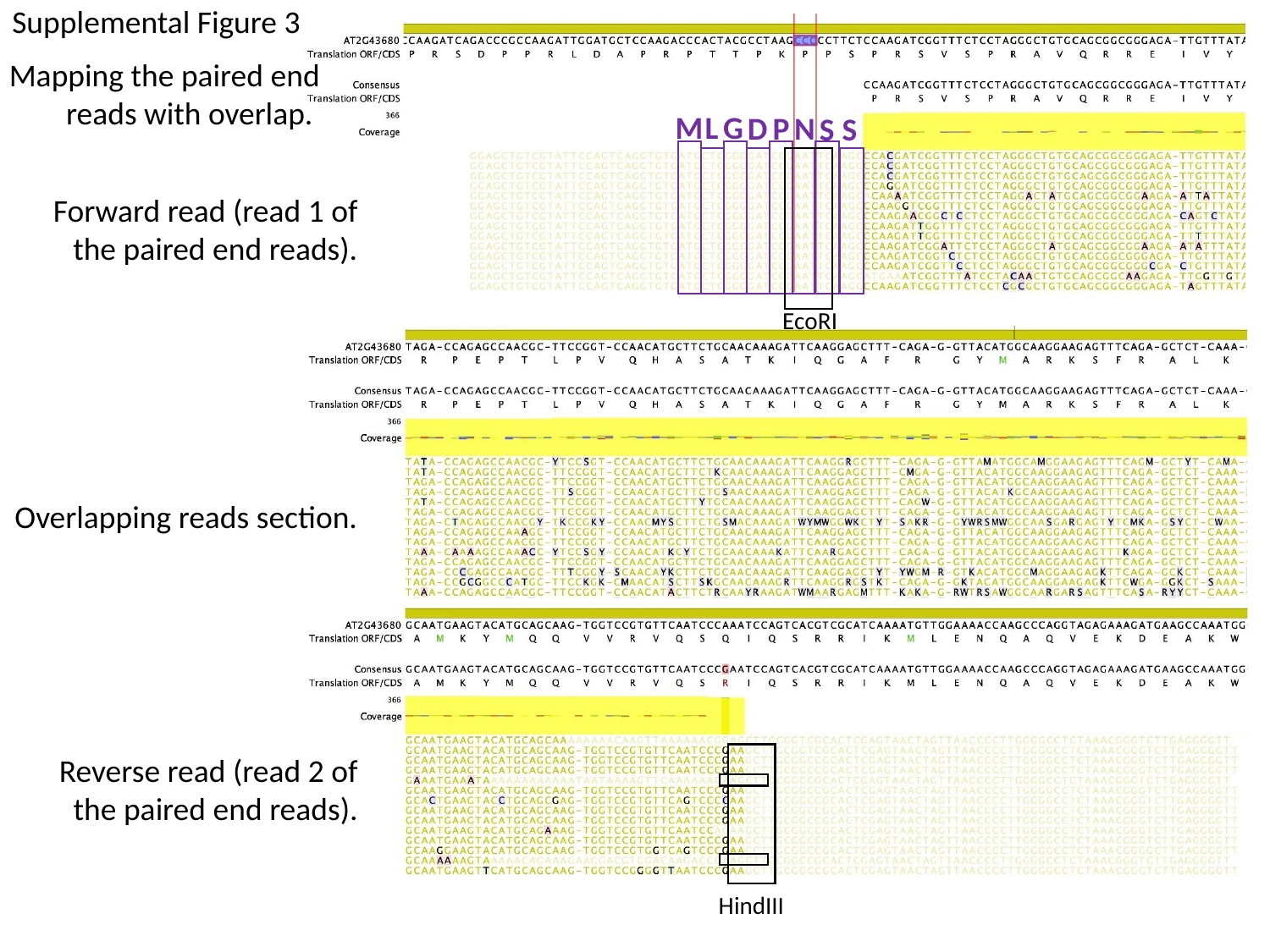

Supplemental Figure 3
M
L
G
N
D
P
S
S
EcoRI
Mapping the paired end reads with overlap.
Forward read (read 1 of the paired end reads).
Overlapping reads section.
HindIII
Reverse read (read 2 of the paired end reads).

## Slide 4
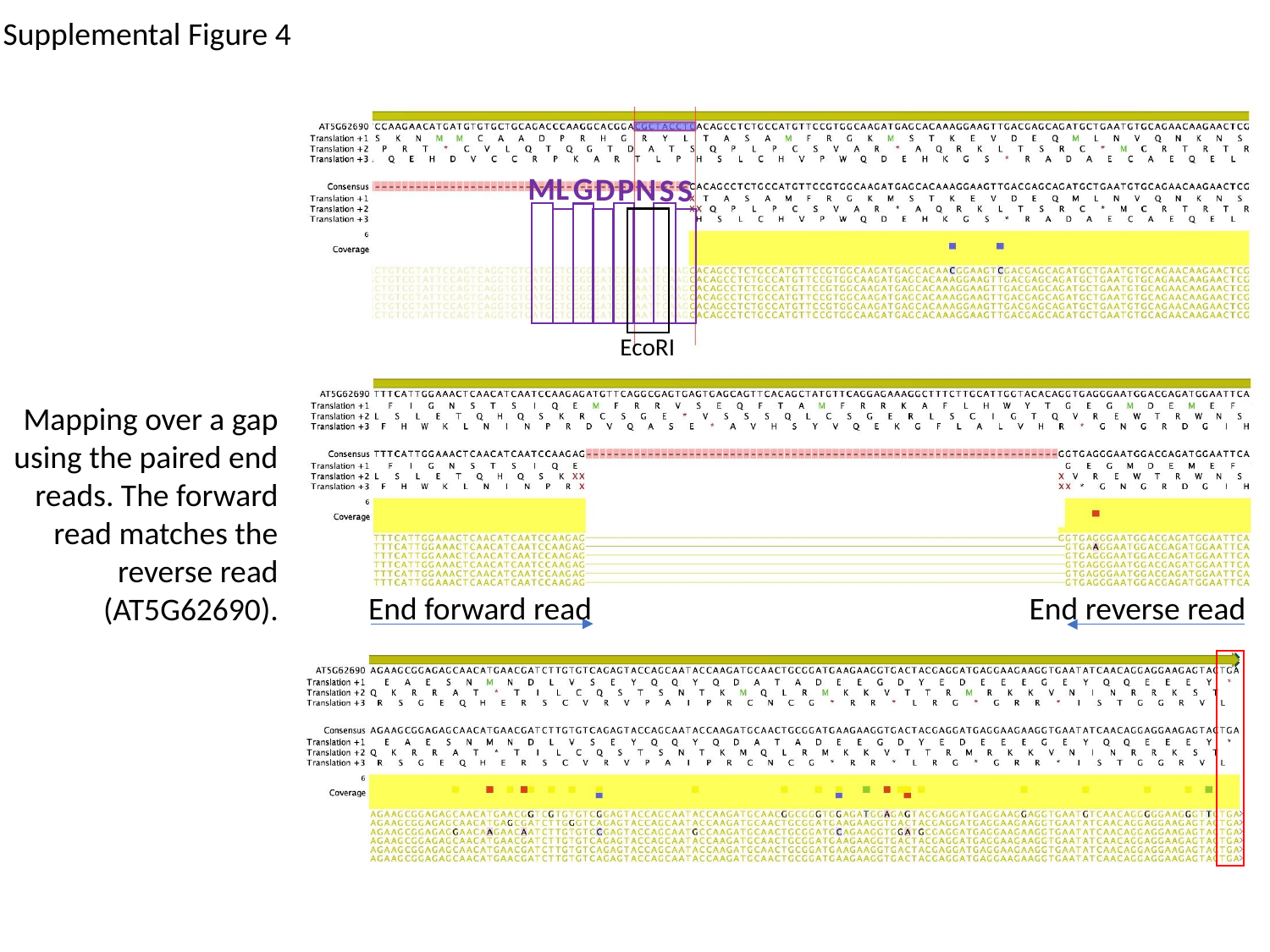

Supplemental Figure 4
M
L
G
N
D
P
S
S
EcoRI
End forward read
End reverse read
Mapping over a gap using the paired end reads. The forward read matches the reverse read (AT5G62690).

## Slide 5
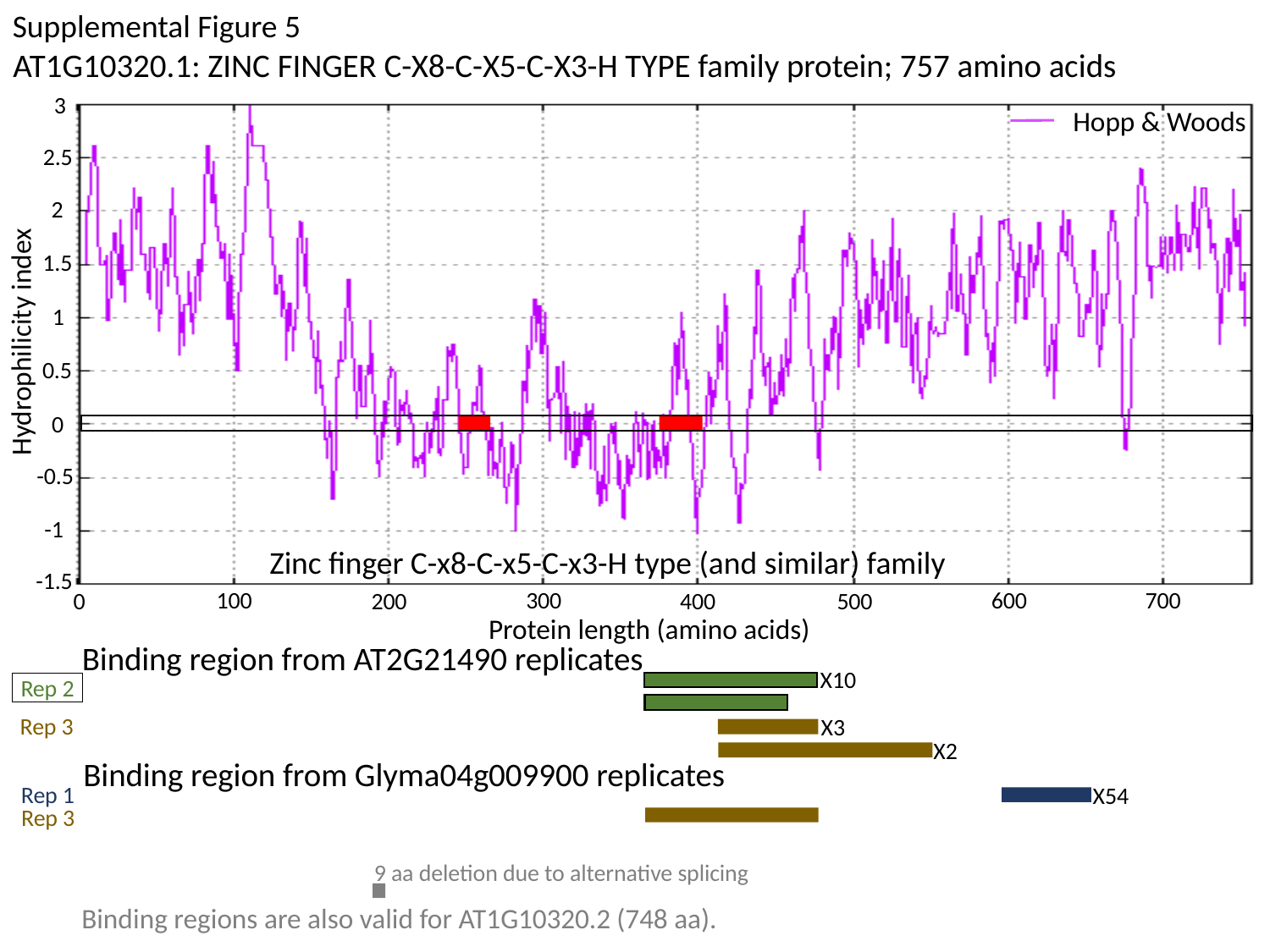

Supplemental Figure 5
AT1G10320.1: ZINC FINGER C-X8-C-X5-C-X3-H TYPE family protein; 757 amino acids
3
 Hopp & Woods
2.5
2
1.5
1
Hydrophilicity index
0.5
0
MEQANEKEEEERHEEAAGEKESFEESKEKAAEMSRKEKRKAMKKLKRKQVRKEIAAKEREEAKAKLNDPAEQERLKAIEEEDARRREKELKDFEESERAWREAMEIKRKKEEEEEAKREEEERRWKDLEELRKLEASGNDECGEDEDGEYEYIEEGPPEIIFQGNEIILKKNKVRVPKKSVVQVDGHESSNAEFVLQISDRPTSNPLPPGSEASANYQNVSSAQQILESVAQEVPNFGTEQDKAHCPFHLKTGACRFGQRCSRVHFYPNKSCTLLMKNMYNGPGITWEQDEGLEYTDEEAELCYEEFYEDVHTEFLKYGELVNFKVCRNGSFHLKGNVYVHYRSLESAILAYQSINGRYFAGKQVNCEFVNISRWKVAICGEYMKSRLKTCSRGSACNFIHCFRNPGGDYEWADHDRPPPRFWIHKMTSLFGYSDEKHMEHESSGSLNDSISDLSTDSHRQPSRRSRSRDHDHANVGSTPSYRSRKYHGDTQDSTREDKLRRHAENCHDGDDSPSRDGSLEREMYKERRYAKDTLHRDSRWSEHSPGHRVGRKRIHGRYSDDDSADGDDYGRRGTGHKRKPRRGTDSGVQEQMDNEKDRKTHRSSRKHSREGSSADKEEGHEHDRVHTVSDKSHRERSKHRHERSSSRYSHEEDSTESRHHQHKESDKKRSVETSPVGYQSDKDRDRSKQRQRYKSDDPESDQSRKGKRQSEENSDRETHKERRHRHRKRRRTQNSDDQNPKESEEVEEEIERWRPV
-0.5
-1
Zinc finger C-x8-C-x5-C-x3-H type (and similar) family
-1.5
100
300
600
700
200
400
500
0
Protein length (amino acids)
Binding region from AT2G21490 replicates
X10
Rep 2
Rep 3
X3
X2
Binding region from Glyma04g009900 replicates
Rep 1
X54
Rep 3
9 aa deletion due to alternative splicing
Binding regions are also valid for AT1G10320.2 (748 aa).

## Slide 6
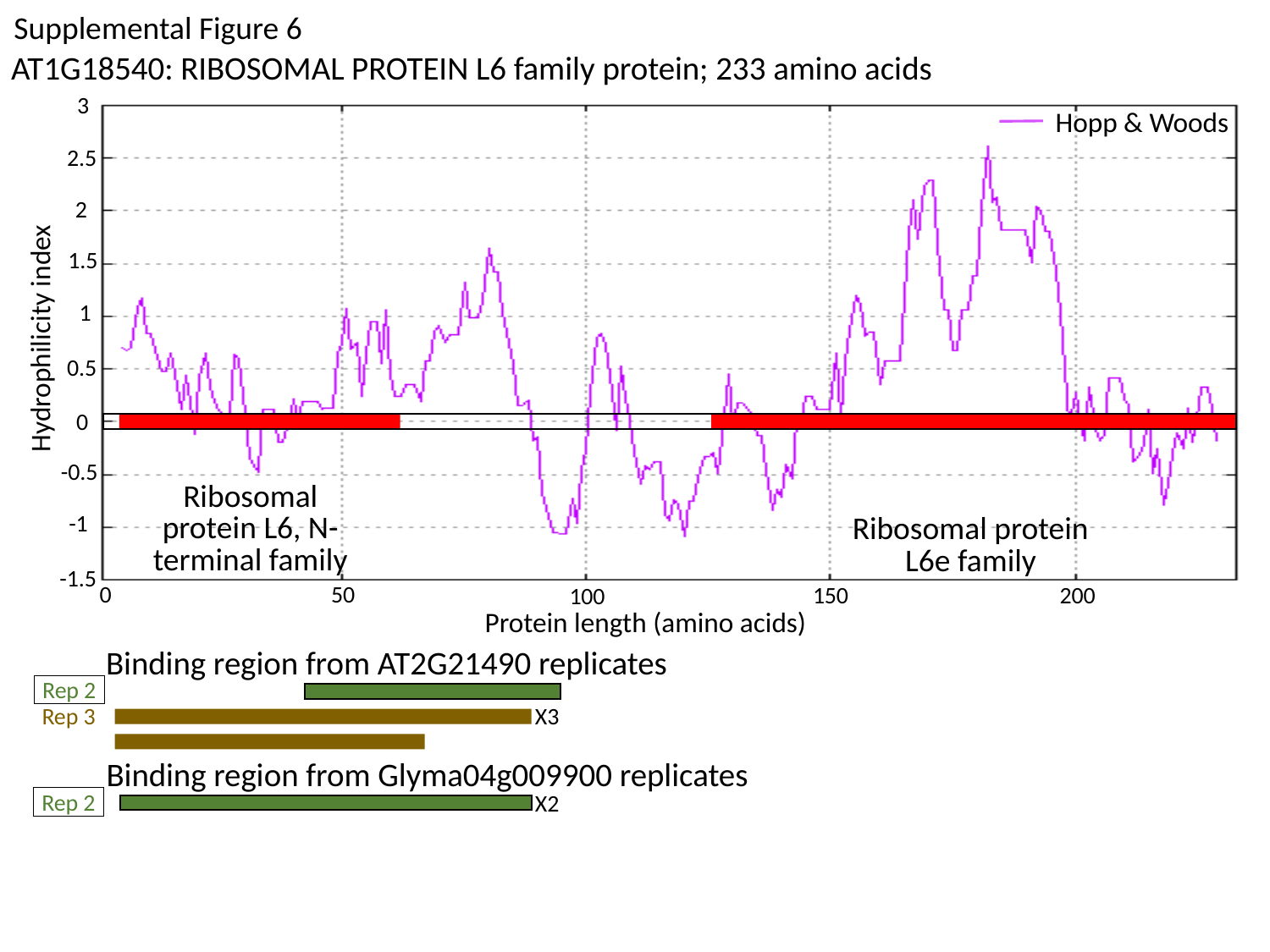

Supplemental Figure 6
AT1G18540: RIBOSOMAL PROTEIN L6 family protein; 233 amino acids
3
Hopp & Woods
2.5
2
1.5
1
Hydrophilicity index
0.5
0
MPAAKRTPKVNRNPDLIRGVGKYSRSQMYHKRGLWAIKAKNGGVFPRHDAQPKVDAPVEKPAKFYPAEDVKKPLVNRRKPKPTKLKASITPGTVLIILAGRFKGKRVVFLKQLSSGLLLVTGPFKINGVPLRRVNQAYVIGTSTKIDISGVNTEKFDDKYFGKVAEKKKKKTEGEFFEAEKEEKKEIPQEKKEDQKTVDAALIKSIEAVPELKVYLGARFSLSQGMKPHELVF
MPAAKRTPKVNRNPDLIRGVGKYSRSQMYHKRGLWAIKAKNGGVFPRHDAQPKVDAPVEKPAKFYPAEDVKKPLVNRRKPKPTKLKASITPGTVLIILAGRFKGKRVVFLKQLSSGLLLVTGPFKINGVPLRRVNQAYVIGTSTKIDISGVNTEKFDDKYFGKVAEKKKKKTEGEFFEAEKEEKKEIPQEKKEDQKTVDAALIKSIEAVPELKVYLGARFSLSQGMKPHELVF
-0.5
Ribosomal protein L6, N-terminal family
-1
Ribosomal protein L6e family
-1.5
0
50
200
150
100
Protein length (amino acids)
Binding region from AT2G21490 replicates
Rep 2
X3
Rep 3
Binding region from Glyma04g009900 replicates
X2
Rep 2

## Slide 7
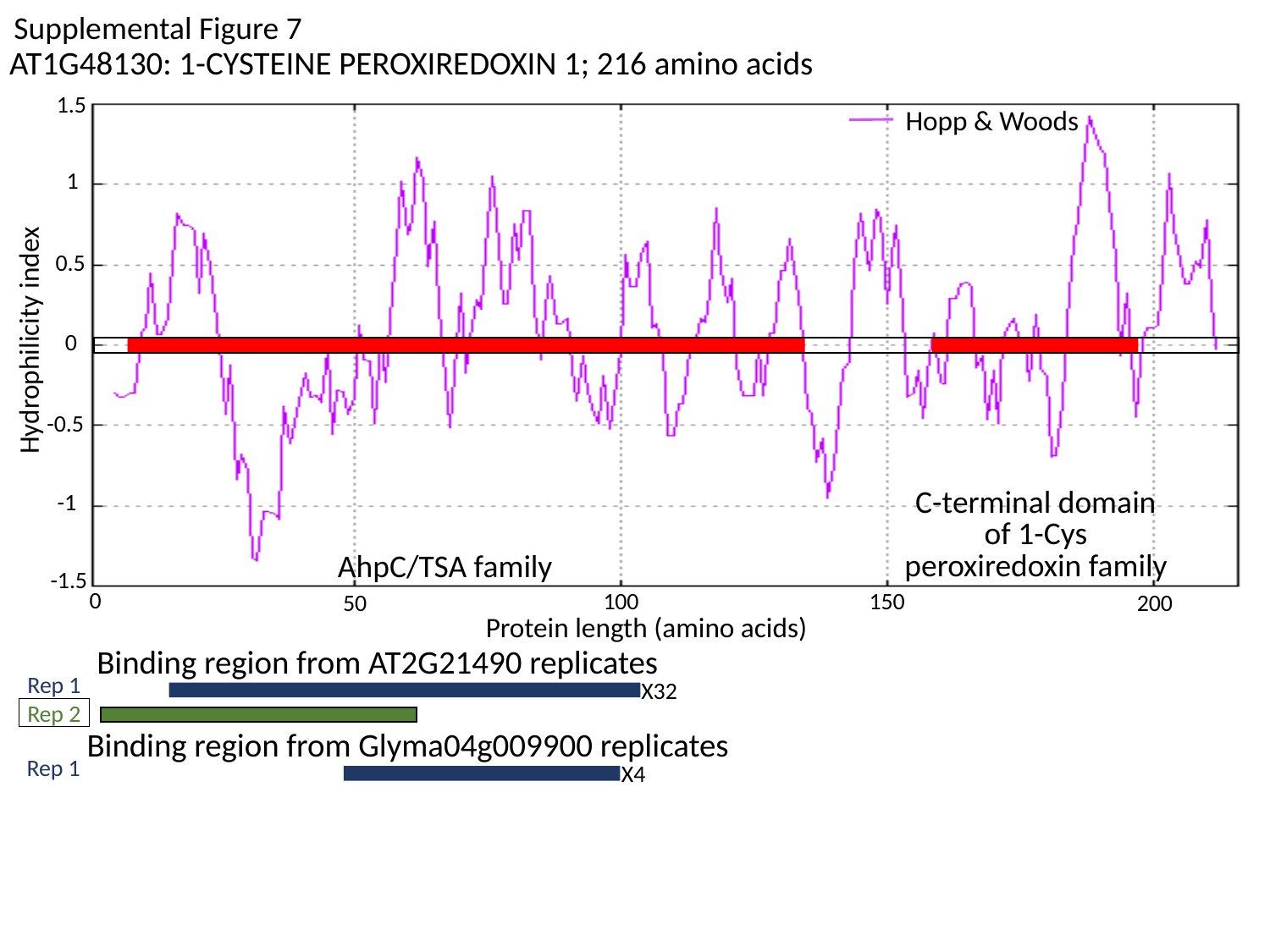

Supplemental Figure 7
AT1G48130: 1-CYSTEINE PEROXIREDOXIN 1; 216 amino acids
1.5
Hopp & Woods
1
0.5
MPGITLGDTVPNLEVETTHDKFKLHDYFANSWTVLFSHPGDFTPVCTTELGAMAKYAHEFDKRGVKLLGLSCDDVQSHKDWIKDIEAFNHGSKVNYPIIADPNKEIIPQLNMIDPIENGPSRALHIVGPDSKIKLSFLYPSTTGRNMDEVLRALDSLLMASKHNNKIATPVNWKPDQPVVISPAVSDEEAKKMFPQGFKTADLPSKKGYLRHTEVS
0
Hydrophilicity index
MPGITLGDTVPNLEVETTHDKFKLHDYFANSWTVLFSHPGDFTPVCTTELGAMAKYAHEFDKRGVKLLGLSCDDVQSHKDWIKDIEAFNHGSKVNYPIIADPNKEIIPQLNMIDPIENGPSRALHIVGPDSKIKLSFLYPSTTGRNMDEVLRALDSLLMASKHNNKIATPVNWKPDQPVVISPAVSDEEAKKMFPQGFKTADLPSKKGYLRHTEVS
-0.5
C-terminal domain of 1-Cys peroxiredoxin family
-1
AhpC/TSA family
-1.5
0
100
150
50
200
Protein length (amino acids)
Binding region from AT2G21490 replicates
Rep 1
X32
Rep 2
Binding region from Glyma04g009900 replicates
Rep 1
X4

## Slide 8
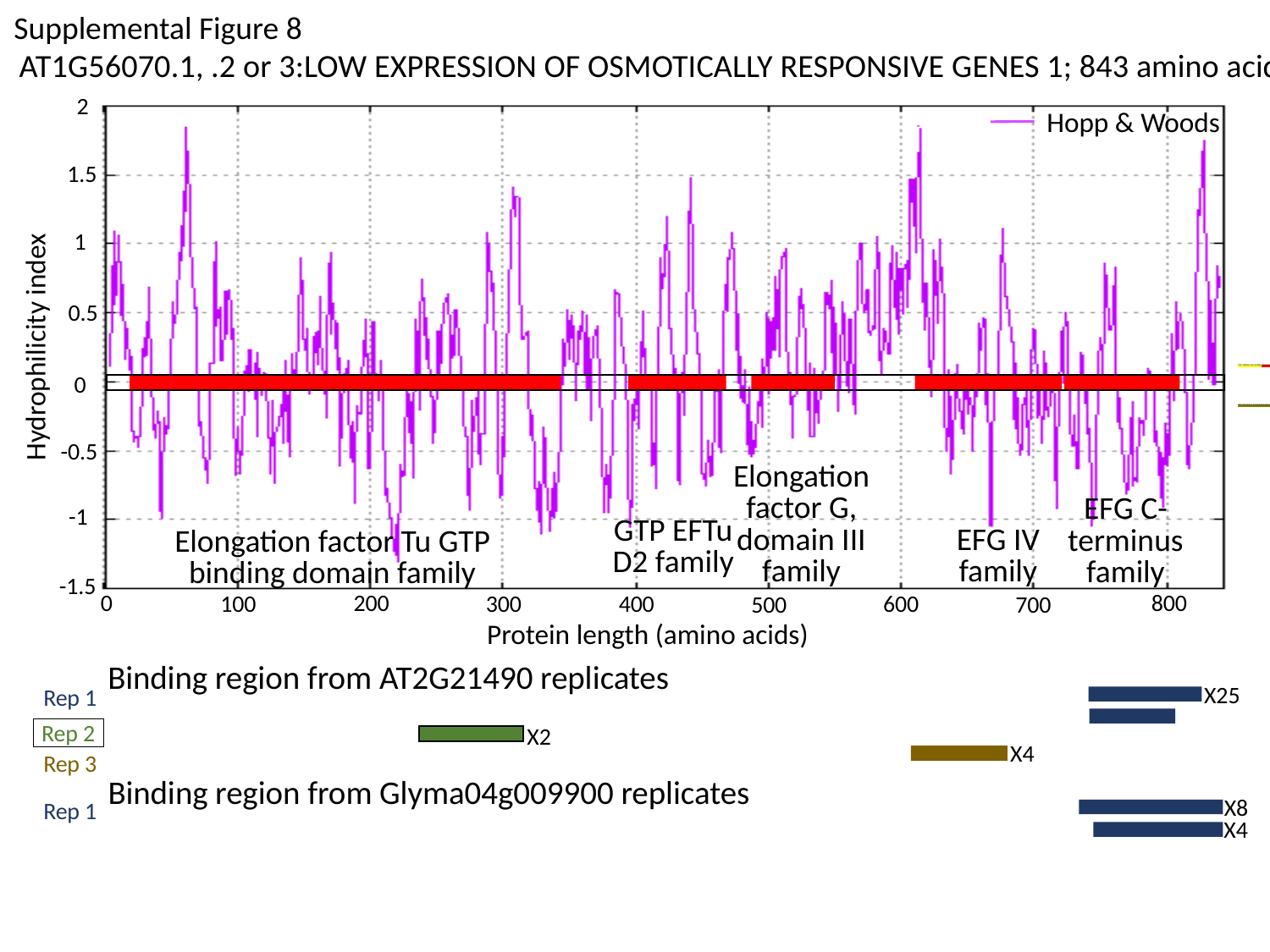

Supplemental Figure 8
AT1G56070.1, .2 or 3:LOW EXPRESSION OF OSMOTICALLY RESPONSIVE GENES 1; 843 amino acids
2
Hopp & Woods
1.5
1
0.5
Hydrophilicity index
MVKFTADELRRIMDYKHNIRNMSVIAHVDHGKSTLTDSLVAAAGIIAQEVAGDVRMTDTRADEAERGITIKSTGISLYYEMTDESLKSFTGARDGNEYLINLIDSPGHVDFSSEVTAALRITDGALVVVDCIEGVCVQTETVLRQALGERIRPVLTVNKMDRCFLELQVDGEEAYQTFSRVIENANVIMATYEDPLLGDVQVYPEKGTVAFSAGLHGWAFTLTNFAKMYASKFGVVESKMMERLWGENFFDPATRKWSGKNTGSPTCKRGFVQFCYEPIKQIIATCMNDQKDKLWPMLAKLGVSMKNDEKELMGKPLMKRVMQTWLPASTALLEMMIFHLPSPHTAQRYRVENLYEGPLDDQYANAIRNCDPNGPLMLYVSKMIPASDKGRFFAFGRVFAGKVSTGMKVRIMGPNYIPGEKKDLYTKSVQRTVIWMGKRQETVEDVPCGNTVAMVGLDQFITKNATLTNEKEVDAHPIRAMKFSVSPVVRVAVQCKVASDLPKLVEGLKRLAKSDPMVVCTMEESGEHIVAGAGELHLEICLKDLQDDFMGGAEIIKSDPVVSFRETVCDRSTRTVMSKSPNKHNRLYMEARPMEEGLAEAIDDGRIGPRDDPKIRSKILAEEFGWDKDLAKKIWAFGPETTGPNMVVDMCKGVQYLNEIKDSVVAGFQWASKEGPLAEENMRGICFEVCDVVLHSDAIHRGGGQVIPTARRVIYASQITAKPRLLEPVYMVEIQAPEGALGGIYSVLNQKRGHVFEEMQRPGTPLYNIKAYLPVVESFGFSSQLRAATSGQAFPQCVFDHWEMMSSDPLEPGTQASVLVADIRKRKGLKEAMTPLSEFEDKL
0
MVKFTADELRRIMDYKHNIRNMSVIAHVDHGKSTLTDSLVAAAGIIAQEVAGDVRMTDTRADEAERGITIKSTGISLYYEMTDESLKSFTGARDGNEYLINLIDSPGHVDFSSEVTAALRITDGALVVVDCIEGVCVQTETVLRQALGERIRPVLTVNKMDRCFLELQVDGEEAYQTFSRVIENANVIMATYEDPLLGDVQVYPEKGTVAFSAGLHGWAFTLTNFAKMYASKFGVVESKMMERLWGENFFDPATRKWSGKNTGSPTCKRGFVQFCYEPIKQIIATCMNDQKDKLWPMLAKLGVSMKNDEKELMGKPLMKRVMQTWLPASTALLEMMIFHLPSPHTAQRYRVENLYEGPLDDQYANAIRNCDPNGPLMLYVSKMIPASDKGRFFAFGRVFAGKVSTGMKVRIMGPNYIPGEKKDLYTKSVQRTVIWMGKRQETVEDVPCGNTVAMVGLDQFITKNATLTNEKEVDAHPIRAMKFSVSPVVRVAVQCKVASDLPKLVEGLKRLAKSDPMVVCTMEESGEHIVAGAGELHLEICLKDLQDDFMGGAEIIKSDPVVSFRETVCDRSTRTVMSKSPNKHNRLYMEARPMEEGLAEAIDDGRIGPRDDPKIRSKILAEEFGWDKDLAKKIWAFGPETTGPNMVVDMCKGVQYLNEIKDSVVAGFQWASKEGPLAEENMRGICFEVCDVVLHSDAIHRGGGQVIPTARRVIYASQITAKPRLLEPVYMVEIQAPEGALGGIYSVLNQKRGHVFEEMQRPGTPLYNIKAYLPVVESFGFSSQLRAATSGQAFPQCVFDHWEMMSSDPLEPGTQASVLVADIRKRKGLKEAMTPLSEFEDKL
-0.5
Elongation factor G, domain III family
EFG C-terminus family
-1
GTP EFTu D2 family
EFG IV family
Elongation factor Tu GTP binding domain family
-1.5
200
0
800
100
300
600
400
700
500
Protein length (amino acids)
Binding region from AT2G21490 replicates
X25
Rep 1
X2
Rep 2
X4
Rep 3
Binding region from Glyma04g009900 replicates
X8
Rep 1
X4

## Slide 9
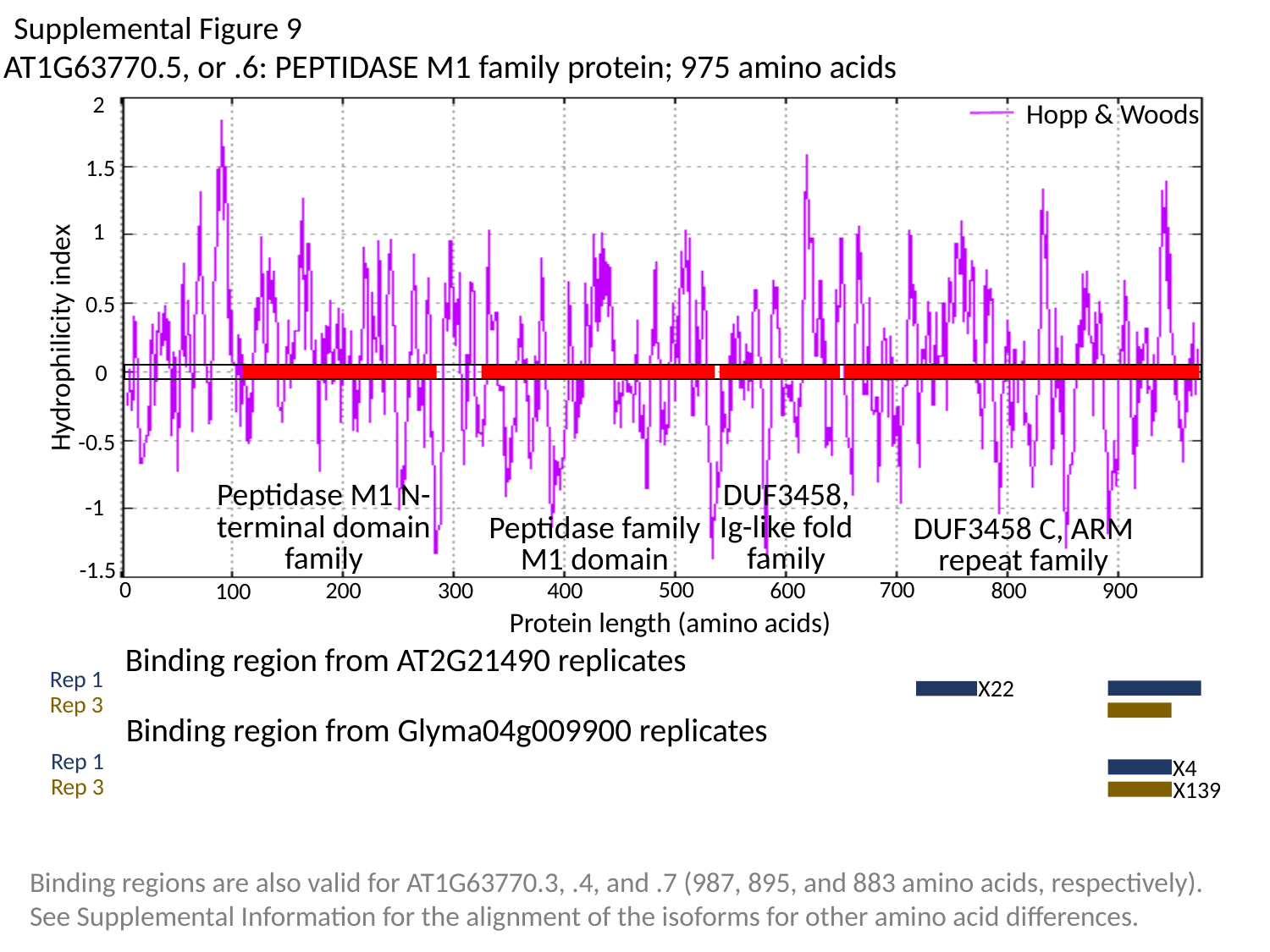

Supplemental Figure 9
AT1G63770.5, or .6: PEPTIDASE M1 family protein; 975 amino acids
2
Hopp & Woods
1.5
1
0.5
Hydrophilicity index
0
MARLIIPCRSSSLARVNLLGLLSRAPVPVRSSCLRSSANRLTQHRPFLTSEAICLRKNRFLPHSVDTHKQNSRRLICSVATESVPDKAEDSKMDAPKEIFLKNYTKPDYYFETVDLSFSLGEEKTIVSSKIKVSPRVKGSSAALVLDGHDLKLLSVKVEGKLLKEGDYQLDSRHLTLPSLPAEESFVLEIDTEIYPHKNTSLEGLYKSSGNFCTQCEAEGFRKITFYQDRPDIMAKYTCRVEGDKTLYPVLLSNGNLISQGDIEGGRHYALWEDPFKKPCYLFALVAGQLVSRDDTFTTRSGRQVSLKIWTPAEDLPKTAHAMYSLKAAMKWDEDVFGLEYDLDLFNIVAVPDFNMGAMENKSLNIFNSKLVLASPETATDADYAAILGVIGHEYFHNWTGNRVTCRDWFQLSLKEGLTVFRDQEFSSDMGSRTVKRIADVSKLRIYQFPQDAGPMAHPVRPHSYIKMDNFYTVTVYEKGAEVVRMYKTLLGTQGFRKGIDLYFERHDEQAVTCEDFFAAMRDANNADFANFLQWYSQAGTPVVKVVSSYNADARTFSLKFSQEIPPTPGQPTKEPTFIPVVVGLLDSSGKDITLSSVHHDGTVQTISGSSTILRVTKKEEEFVFSDIPERPVPSLFRGFSAPVRVETDLSNDDLFFLLAHDSDEFNRWEAGQVLARKLMLNLVSDFQQNKPLALNPKFVQGLGSVLSDSSLDKEFIAKAITLPGEGEIMDMMAVADPDAVHAVRKFVRKQLASELKEELLKIVENNRSTEAYVFDHSNMARRALKNTALAYLASLEDPAYMELALNEYKMATNLTDQFAALAALSQNPGKTRDDILADFYNKWQDDYLVVNKWFLLQSTSDIPGNVENVKKLLDHPAFDLRNPNKVYSLIGGFCGSPVNFHAKDGSGYKFLGDIVVQLDKLNPQVASRMVSAFSRWKRYDETRQGLAKAQLEMIMSANGLSENVFEIASKSLAA
-0.5
MARLIIPCRSSSLARVNLLGLLSRAPVPVRSSCLRSSANRLTQHRPFLTSEAICLRKNRFLPHSVDTHKQNSRRLICSVATESVPDKAEDSKMDAPKEIFLKNYTKPDYYFETVDLSFSLGEEKTIVSSKIKVSPRVKGSSAALVLDGHDLKLLSVKVEGKLLKEGDYQLDSRHLTLPSLPAEESFVLEIDTEIYPHKNTSLEGLYKSSGNFCTQCEAEGFRKITFYQDRPDIMAKYTCRVEGDKTLYPVLLSNGNLISQGDIEGGRHYALWEDPFKKPCYLFALVAGQLVSRDDTFTTRSGRQVSLKIWTPAEDLPKTAHAMYSLKAAMKWDEDVFGLEYDLDLFNIVAVPDFNMGAMENKSLNIFNSKLVLASPETATDADYAAILGVIGHEYFHNWTGNRVTCRDWFQLSLKEGLTVFRDQEFSSDMGSRTVKRIADVSKLRIYQFPQDAGPMAHPVRPHSYIKMDNFYTVTVYEKGAEVVRMYKTLLGTQGFRKGIDLYFERHDEQAVTCEDFFAAMRDANNADFANFLQWYSQAGTPVVKVVSSYNADARTFSLKFSQEIPPTPGQPTKEPTFIPVVVGLLDSSGKDITLSSVHHDGTVQTISGSSTILRVTKKEEEFVFSDIPERPVPSLFRGFSAPVRVETDLSNDDLFFLLAHDSDEFNRWEAGQVLARKLMLNLVSDFQQNKPLALNPKFVQGLGSVLSDSSLDKEFIAKAITLPGEGEIMDMMAVADPDAVHAVRKFVRKQLASELKEELLKIVENNRSTEAYVFDHSNMARRALKNTALAYLASLEDPAYMELALNEYKMATNLTDQFAALAALSQNPGKTRDDILADFYNKWQDDYLVVNKWFLLQSTSDIPGNVENVKKLLDHPAFDLRNPNKVYSLIGGFCGSPVNFHAKDGSGYKFLGDIVVQLDKLNPQVASRMVSAFSRWKRYDETRQGLAKAQLEMIMSANGLSENVFEIASKSLAA
Peptidase M1 N-terminal domain family
DUF3458, Ig-like fold family
-1
Peptidase family M1 domain
DUF3458 C, ARM repeat family
-1.5
0
500
700
800
600
300
900
200
400
100
Protein length (amino acids)
Binding region from AT2G21490 replicates
Rep 1
X22
Rep 3
Binding region from Glyma04g009900 replicates
Rep 1
X4
Rep 3
X139
MARLIIPCRSSSLARVNLLGLLSRAPVPVRSSCLRSSANRLTQHRPFLTSEAICLRKNRFLPHSVDTHKQNSRRLICSVATESVPDKAEDSKMDAPKEIFLKNYTKPDYYFETVDLSFSLGEEKTIVSSKIKVSPRVKGSSAALVLDGHDLKLLSVKVEGKLLKEGDYQLDSRHLTLPSLPAEESFVLEIDTEIYPHKNTSLEGLYKSSGNFCTQCEAEGFRKITFYQDRPDIMAKYTCRVEGDKTLYPVLLSNGNLISQGDIEGGRHYALWEDPFKKPCYLFALVAGQLVSRDDTFTTRSGRQVSLKIWTPAEDLPKTAHAMYSLKAAMKWDEDVFGLEYDLDLFNIVAVPDFNMGAMENKSLNIFNSKLVLASPETATDADYAAILGVIGHEYFHNWTGNRVTCRDWFQLSLKEGLTVFRDQEFSSDMGSRTVKRIADVSKLRIYQFPQDAGPMAHPVRPHSYIKMDNFYTVTVYEKGAEVVRMYKTLLGTQGFRKGIDLYFERHDEQAVTCEDFFAAMRDANNADFANFLQWYSQAGTPVVKVVSSYNADARTFSLKFSQEIPPTPGQPTKEPTFIPVVVGLLDSSGKDITLSSVHHDGTVQTISGSSTILRVTKKEEEFVFSDIPERPVPSLFRGFSAPVRVETDLSNDDLFFLLAHDSDEFNRWEAGQVLARKLMLNLVSDFQQNKPLALNPKFVQGLGSVLSDSSLDKEFIAKAITLPGEGEIMDMMAVADPDAVHAVRKFVRKQLASELKEELLKIVENNRSTEAYVFDHSNMARRALKNTALAYLASLEDPAYMELALNEYKMATNLTDQFAALAALSQNPGKTRDDILADFYNKWQDDYLVVNKWFLLQSTSDIPGNVENVKKLLDHPAFDLRNPNKVYSLIGGFCGSPVNFHAKDGSGYKFLGDIVVQLDKLNPQVASRMVSAFSRWKRYDETRQGLAKAQLEMIMSANGLSENVFEIASKSLAA
Binding regions are also valid for AT1G63770.3, .4, and .7 (987, 895, and 883 amino acids, respectively).
See Supplemental Information for the alignment of the isoforms for other amino acid differences.

## Slide 10
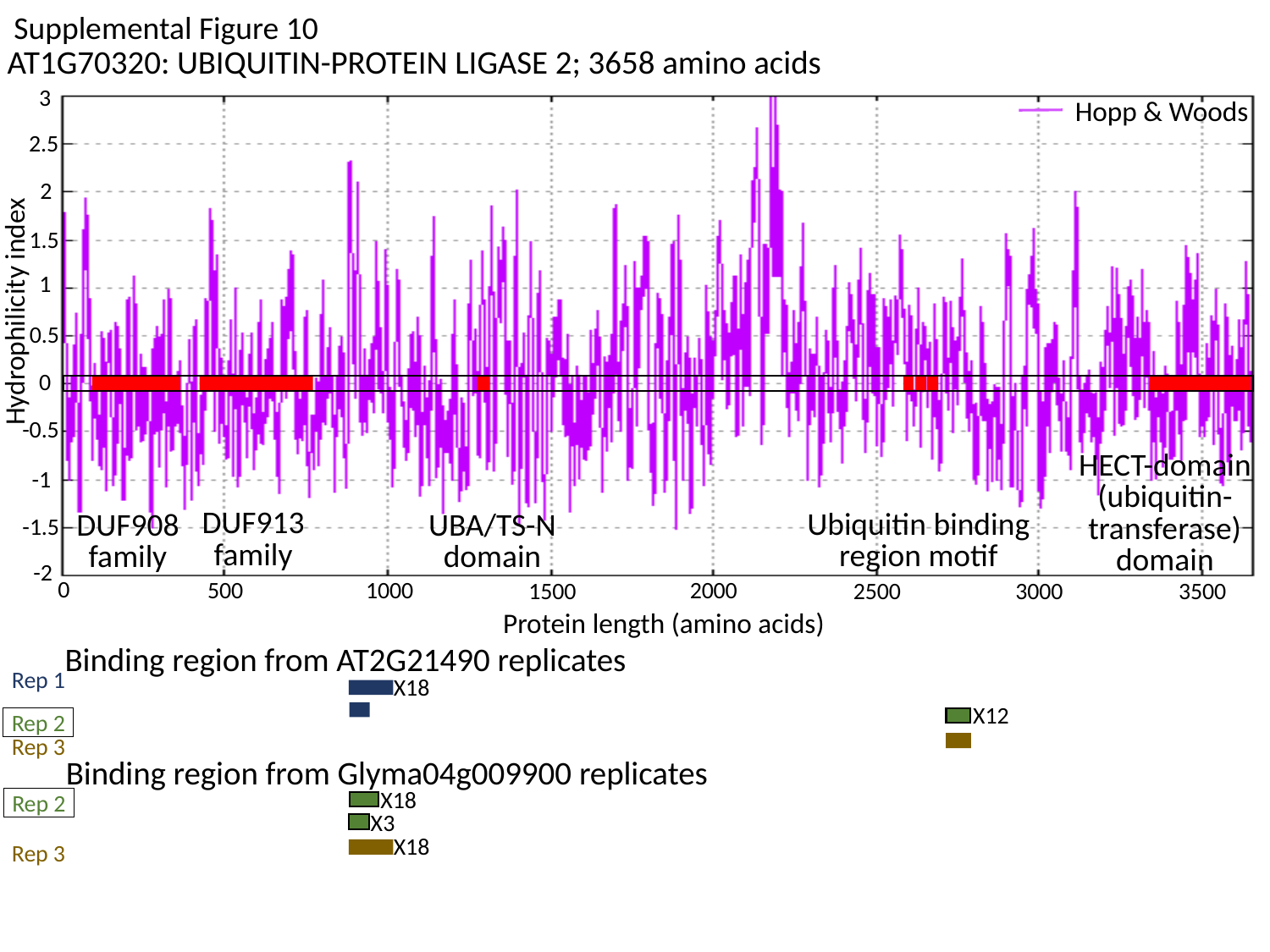

Supplemental Figure 10
AT1G70320: UBIQUITIN-PROTEIN LIGASE 2; 3658 amino acids
3
Hopp & Woods
2.5
2
1.5
1
Hydrophilicity index
0.5
0
-0.5
HECT-domain (ubiquitin-transferase) domain
-1
DUF913 family
Ubiquitin binding region motif
UBA/TS-N domain
DUF908 family
-1.5
-2
0
500
1000
2000
3500
2500
1500
3000
Protein length (amino acids)
Binding region from AT2G21490 replicates
Rep 1
X18
X12
Rep 2
Rep 3
Binding region from Glyma04g009900 replicates
X18
Rep 2
X3
X18
Rep 3

## Slide 11
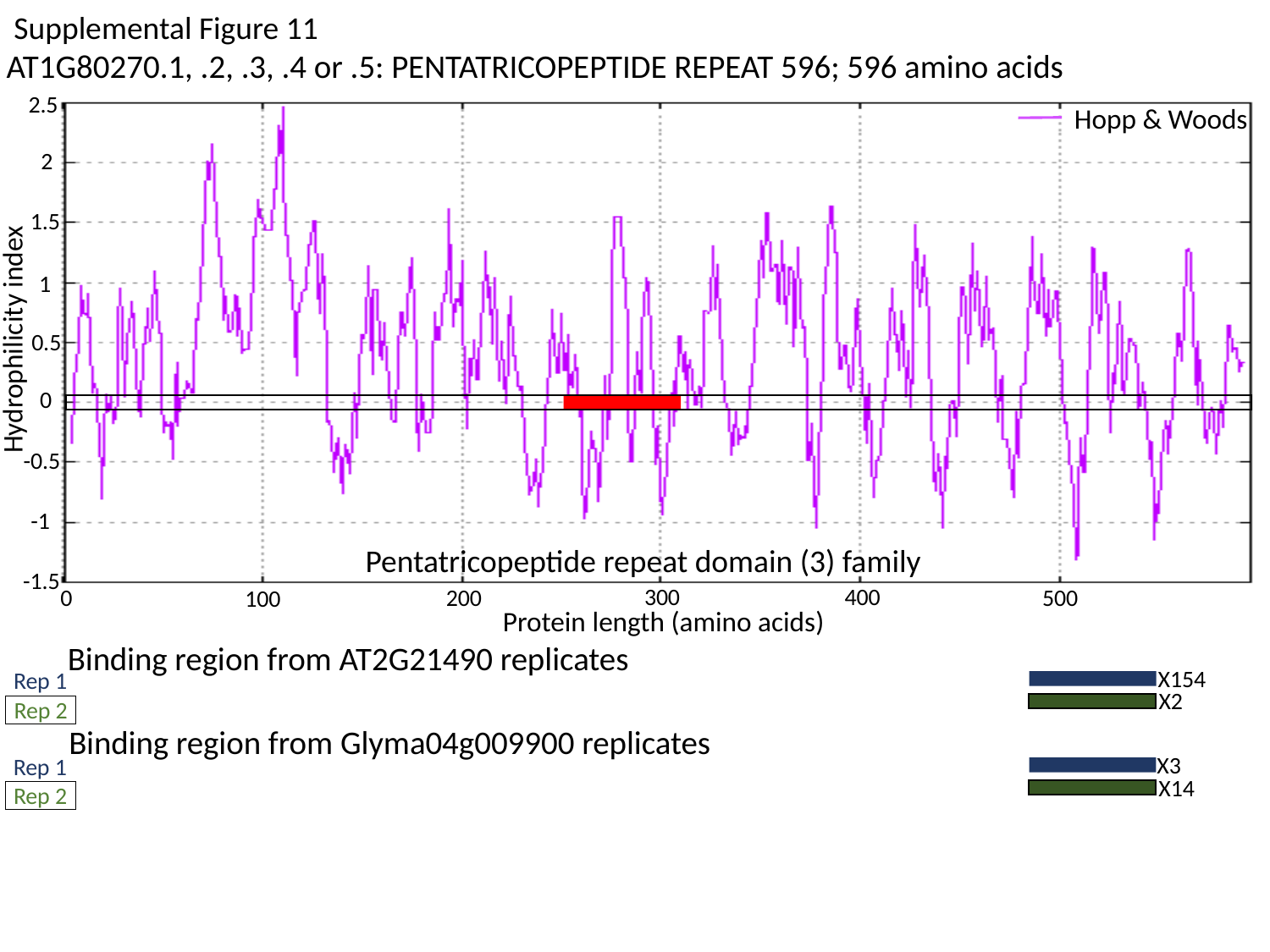

Supplemental Figure 11
AT1G80270.1, .2, .3, .4 or .5: PENTATRICOPEPTIDE REPEAT 596; 596 amino acids
2.5
Hopp & Woods
2
1.5
1
0.5
Hydrophilicity index
0
MFALSKVLRRTQRLRLGACSAVFSKDIQLGGERSFDSNSIASTKREAVPRFYEISSLSNRALSSSAGTKSDQEEDDLEDGFSELEGSKSGQGSTSSDEDEGKLSADEEEEEELDLIETDVSRKTVEKKQSELFKTIVSAPGLSIGSALDKWVEEGNEITRVEIAKAMLQLRRRRMYGRALQMSEWLEANKKIEMTERDYASRLDLTVKIRGLEKGEACMQKIPKSFKGEVLYRTLLANCVAAGNVKKSELVFNKMKDLGFPLSGFTCDQMLLLHKRIDRKKIADVLLLMEKENIKPSLLTYKILIDVKGATNDISGMEQILETMKDEGVELDFQTQALTARHYSGAGLKDKAEKVLKEMEGESLEANRRAFKDLLSIYASLGREDEVKRIWKICESKPYFEESLAAIQAFGKLNKVQEAEAIFEKIVKMDRRASSSTYSVLLRVYVDHKMLSKGKDLVKRMAESGCRIEATTWDALIKLYVEAGEVEKADSLLDKASKQSHTKLMMNSFMYIMDEYSKRGDVHNTEKIFLKMREAGYTSRLRQFQALMQAYINAKSPAYGMRDRLKADNIFPNKSMAAQLAQGDPFKKTAISDILD
-0.5
MFALSKVLRRTQRLRLGACSAVFSKDIQLGGERSFDSNSIASTKREAVPRFYEISSLSNRALSSSAGTKSDQEEDDLEDGFSELEGSKSGQGSTSSDEDEGKLSADEEEEEELDLIETDVSRKTVEKKQSELFKTIVSAPGLSIGSALDKWVEEGNEITRVEIAKAMLQLRRRRMYGRALQMSEWLEANKKIEMTERDYASRLDLTVKIRGLEKGEACMQKIPKSFKGEVLYRTLLANCVAAGNVKKSELVFNKMKDLGFPLSGFTCDQMLLLHKRIDRKKIADVLLLMEKENIKPSLLTYKILIDVKGATNDISGMEQILETMKDEGVELDFQTQALTARHYSGAGLKDKAEKVLKEMEGESLEANRRAFKDLLSIYASLGREDEVKRIWKICESKPYFEESLAAIQAFGKLNKVQEAEAIFEKIVKMDRRASSSTYSVLLRVYVDHKMLSKGKDLVKRMAESGCRIEATTWDALIKLYVEAGEVEKADSLLDKASKQSHTKLMMNSFMYIMDEYSKRGDVHNTEKIFLKMREAGYTSRLRQFQALMQAYINAKSPAYGMRDRLKADNIFPNKSMAAQLAQGDPFKKTAISDILD
-1
Pentatricopeptide repeat domain (3) family
-1.5
300
400
0
200
500
100
Protein length (amino acids)
Binding region from AT2G21490 replicates
X154
Rep 1
X2
Rep 2
Binding region from Glyma04g009900 replicates
X3
Rep 1
X14
Rep 2

## Slide 12
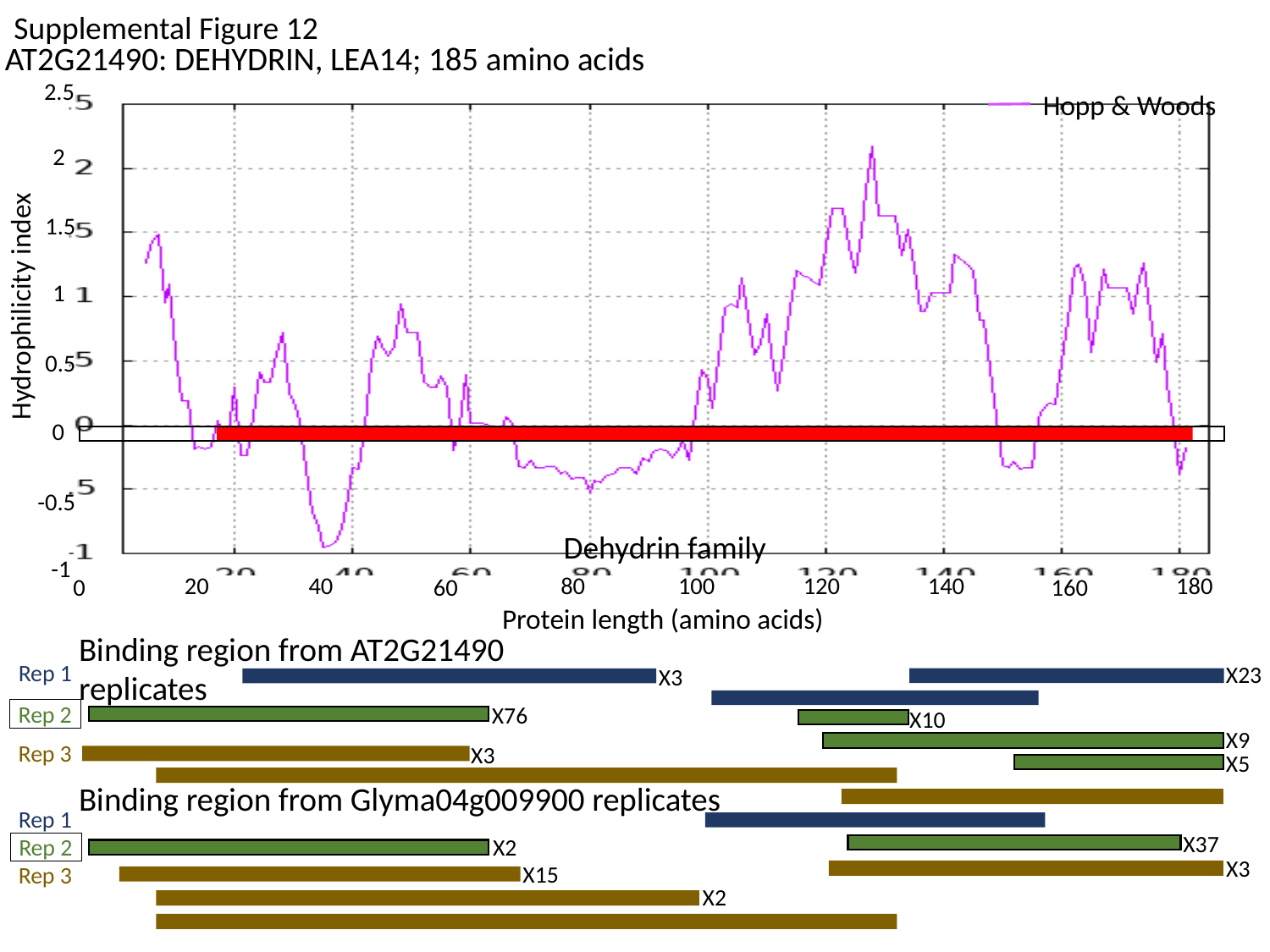

Supplemental Figure 12
AT2G21490: DEHYDRIN, LEA14; 185 amino acids
2.5
Hopp & Woods
2
1.5
1
Hydrophilicity index
0.5
MADLRDEKGNPIHLTDTQGNPIVDLTDEHGNPMYLTGVVSSTPQHKESTTSDIAEHPTSTVGETHPAAAPAGAGAATAATATGVSAGTGATTTGQQHHGSLEEHLRRSGSSSSSSSEDDGQGGRRKKSIKEKIKEKFGSGKHKDEQTPATATTTGPATTDQPHEKKGILEKIKDKLPGHHNHNHP
0
MADLRDEKGNPIHLTDTQGNPIVDLTDEHGNPMYLTGVVSSTPQHKESTTSDIAEHPTSTVGETHPAAAPAGAGAATAATATGVSAGTGATTTGQQHHGSLEEHLRRSGSSSSSSSEDDGQGGRRKKSIKEKIKEKFGSGKHKDEQTPATATTTGPATTDQPHEKKGILEKIKDKLPGHHNHNHP
MADLRDEKGNPIHLTDTQGNPIVDLTDEHGNPMYLTGVVSSTPQHKESTTSDIAEHPTSTVGETHPAAAPAGAGAATAATATGVSAGTGATTTGQQHHGSLEEHLRRSGSSSSSSSEDDGQGGRRKKSIKEKIKEKFGSGKHKDEQTPATATTTGPATTDQPHEKKGILEKIKDKLPGHHNHNHP
-0.5
Dehydrin family
-1
100
140
120
40
80
180
20
160
0
60
Protein length (amino acids)
Binding region from AT2G21490 replicates
Rep 1
X23
X3
X76
X10
Rep 2
X9
Rep 3
X3
X5
Binding region from Glyma04g009900 replicates
Rep 1
X37
X2
Rep 2
X3
X15
Rep 3
X2

## Slide 13
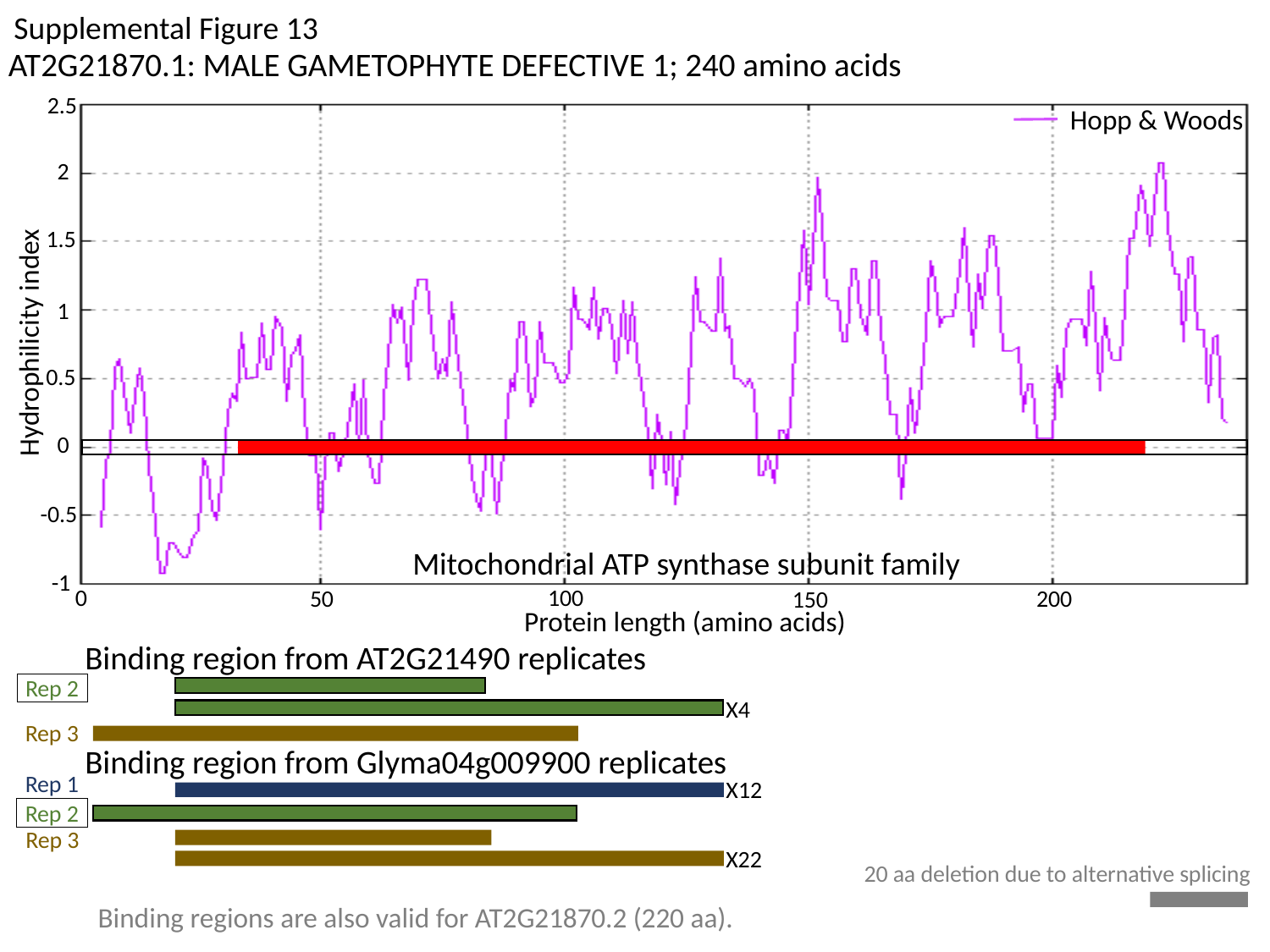

Supplemental Figure 13
AT2G21870.1: MALE GAMETOPHYTE DEFECTIVE 1; 240 amino acids
2.5
Hopp & Woods
2
1.5
1
Hydrophilicity index
0.5
0
MAYASRFLSRSKQLQGGLVILQQQHAIPVRAFAKEAARPTFKGDEMLKGVFFDIKNKFQAAVDILRKEKITLDPEDPAAVKQYANVMKTIRQKADMFSESQRIKHDIDTETQDIPDARAYLLKLQEIRTRRGLTDELGAEAMMFEALEKVEKDIKKPLLRSDKKGMDLLVAEFEKGNKKLGIRKEDLPKYEENLELSMAKAQLDELKSDAVEAMESQKKKEEFQDEEMPDVKSLDIRNFI
MAYASRFLSRSKQLQGGLVILQQQHAIPVRAFAKEAARPTFKGDEMLKGVFFDIKNKFQAAVDILRKEKITLDPEDPAAVKQYANVMKTIRQKADMFSESQRIKHDIDTETQDIPDARAYLLKLQEIRTRRGLTDELGAEAMMFEALEKVEKDIKKPLLRSDKKGMDLLVAEFEKGNKKLGIRKEDLPKYEENLELSMAKAQLDELKSDAVEAMESQKKKEEFQDEEMPDVKSLDIRNFI
-0.5
Mitochondrial ATP synthase subunit family
-1
100
0
50
200
150
Protein length (amino acids)
Binding region from AT2G21490 replicates
Rep 2
X4
Rep 3
Binding region from Glyma04g009900 replicates
Rep 1
X12
Rep 2
Rep 3
X22
MTLITDHVCSLSAGDRVDSLLDALSRLRGTLRLKTKMADTGKGSSVAGCNDSCGCPSPCPGGNSCRCRMREASAGDQGHMVCPCGEHCGCNPCNCPKTQTQTSAKGCTCGEGCTCASCAT
20 aa deletion due to alternative splicing
Binding regions are also valid for AT2G21870.2 (220 aa).

## Slide 14
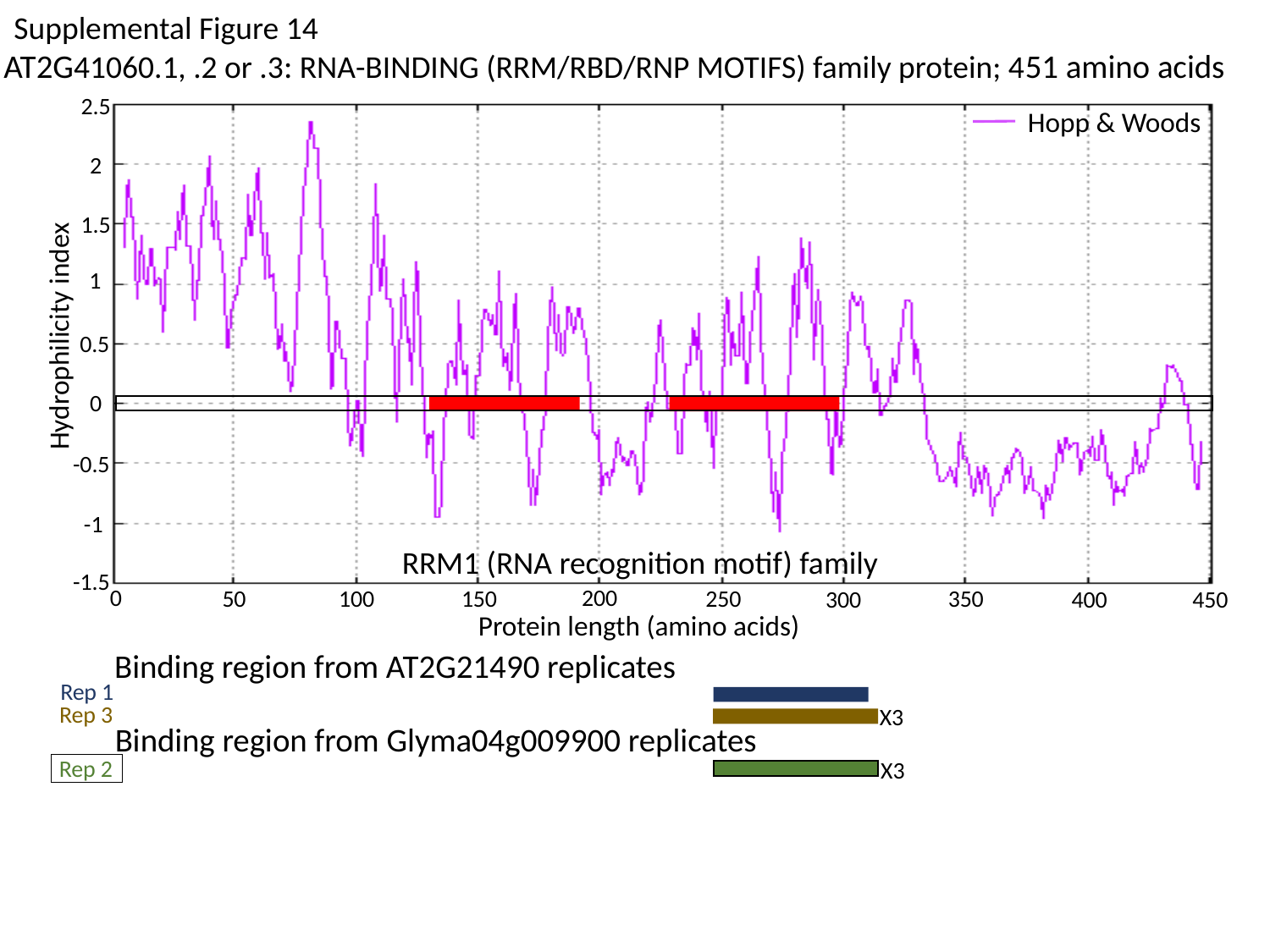

Supplemental Figure 14
AT2G41060.1, .2 or .3: RNA-BINDING (RRM/RBD/RNP MOTIFS) family protein; 451 amino acids
2.5
Hopp & Woods
2
1.5
1
Hydrophilicity index
0.5
0
MTKKRKLESESNETSEPTEKQQQQCEKEDPEIRNVDNQRDDDEQVVEQDTLKEMHEEEAKGEDNIEAETSSGSGNQGNEDDDEEEPIEDLLEPFSKDQLLILLKEAAERHRDVANRIRIVADEDLVHRKIFVHGLGWDTKADSLIDAFKQYGEIEDCKCVVDKVSGQSKGYGFILFKSRSGARNALKQPQKKIGTRMTACQLASIGPVQGNPVVAPAQHFNPENVQRKIYVSNVSADIDPQKLLEFFSRFGEIEEGPLGLDKATGRPKGFALFVYRSLESAKKALEEPHKTFEGHVLHCHKANDGPKQVKQHQHNHNSHNQNSRYQRNDNNGYGAPGGHGHFIAGNNQAVQAFNPAIGQALTALLASQGAGLGLNQAFGQALLGTLGTASPGAVGGMPSGYGTQANISPGVYPGYGAQAGYQGGYQTQQPGQGGAGRGQHGAGYGGPYMGR
MTKKRKLESESNETSEPTEKQQQQCEKEDPEIRNVDNQRDDDEQVVEQDTLKEMHEEEAKGEDNIEAETSSGSGNQGNEDDDEEEPIEDLLEPFSKDQLLILLKEAAERHRDVANRIRIVADEDLVHRKIFVHGLGWDTKADSLIDAFKQYGEIEDCKCVVDKVSGQSKGYGFILFKSRSGARNALKQPQKKIGTRMTACQLASIGPVQGNPVVAPAQHFNPENVQRKIYVSNVSADIDPQKLLEFFSRFGEIEEGPLGLDKATGRPKGFALFVYRSLESAKKALEEPHKTFEGHVLHCHKANDGPKQVKQHQHNHNSHNQNSRYQRNDNNGYGAPGGHGHFIAGNNQAVQAFNPAIGQALTALLASQGAGLGLNQAFGQALLGTLGTASPGAVGGMPSGYGTQANISPGVYPGYGAQAGYQGGYQTQQPGQGGAGRGQHGAGYGGPYMGR
-0.5
-1
RRM1 (RNA recognition motif) family
-1.5
0
200
50
100
150
250
350
400
450
300
Protein length (amino acids)
Binding region from AT2G21490 replicates
Rep 1
Rep 3
X3
Binding region from Glyma04g009900 replicates
X3
Rep 2

## Slide 15
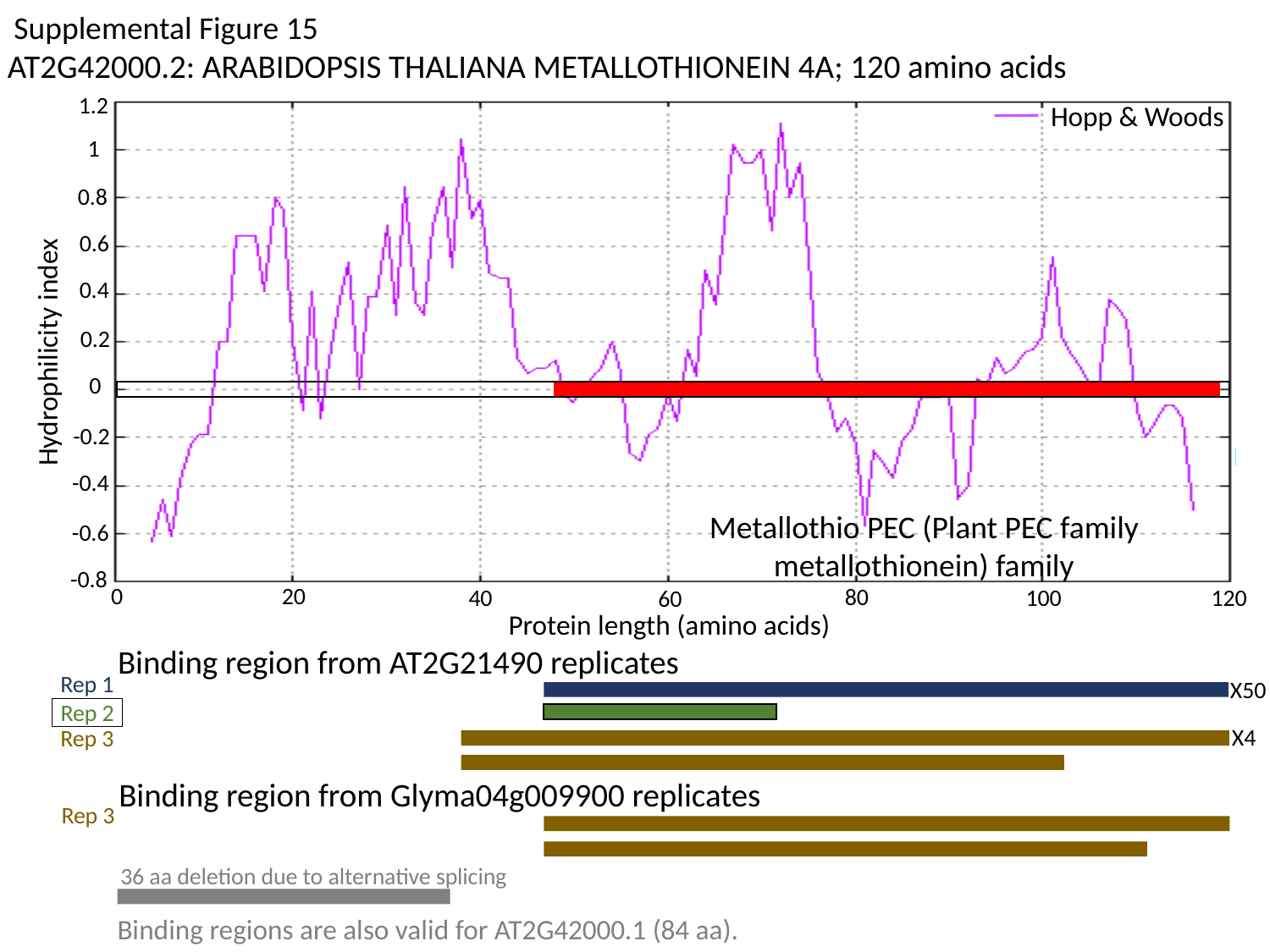

Supplemental Figure 15
AT2G42000.2: ARABIDOPSIS THALIANA METALLOTHIONEIN 4A; 120 amino acids
1.2
Hopp & Woods
1
0.8
0.6
0.4
0.2
Hydrophilicity index
MTLITDHVCSLSAGDRVDSLLDALSRLRGTLRLKTKMADTGKGSSVAGCNDSCGCPSPCPGGNSCRCRMREASAGDQGHMVCPCGEHCGCNPCNCPKTQTQTSAKGCTCGEGCTCASCAT
0
-0.2
MTLITDHVCSLSAGDRVDSLLDALSRLRGTLRLKTKMADTGKGSSVAGCNDSCGCPSPCPGGNSCRCRMREASAGDQGHMVCPCGEHCGCNPCNCPKTQTQTSAKGCTCGEGCTCASCAT
-0.4
Metallothio PEC (Plant PEC family metallothionein) family
-0.6
-0.8
20
0
80
120
40
100
60
Protein length (amino acids)
Binding region from AT2G21490 replicates
Rep 1
X50
Rep 2
X4
Rep 3
Binding region from Glyma04g009900 replicates
Rep 3
36 aa deletion due to alternative splicing
Binding regions are also valid for AT2G42000.1 (84 aa).

## Slide 16
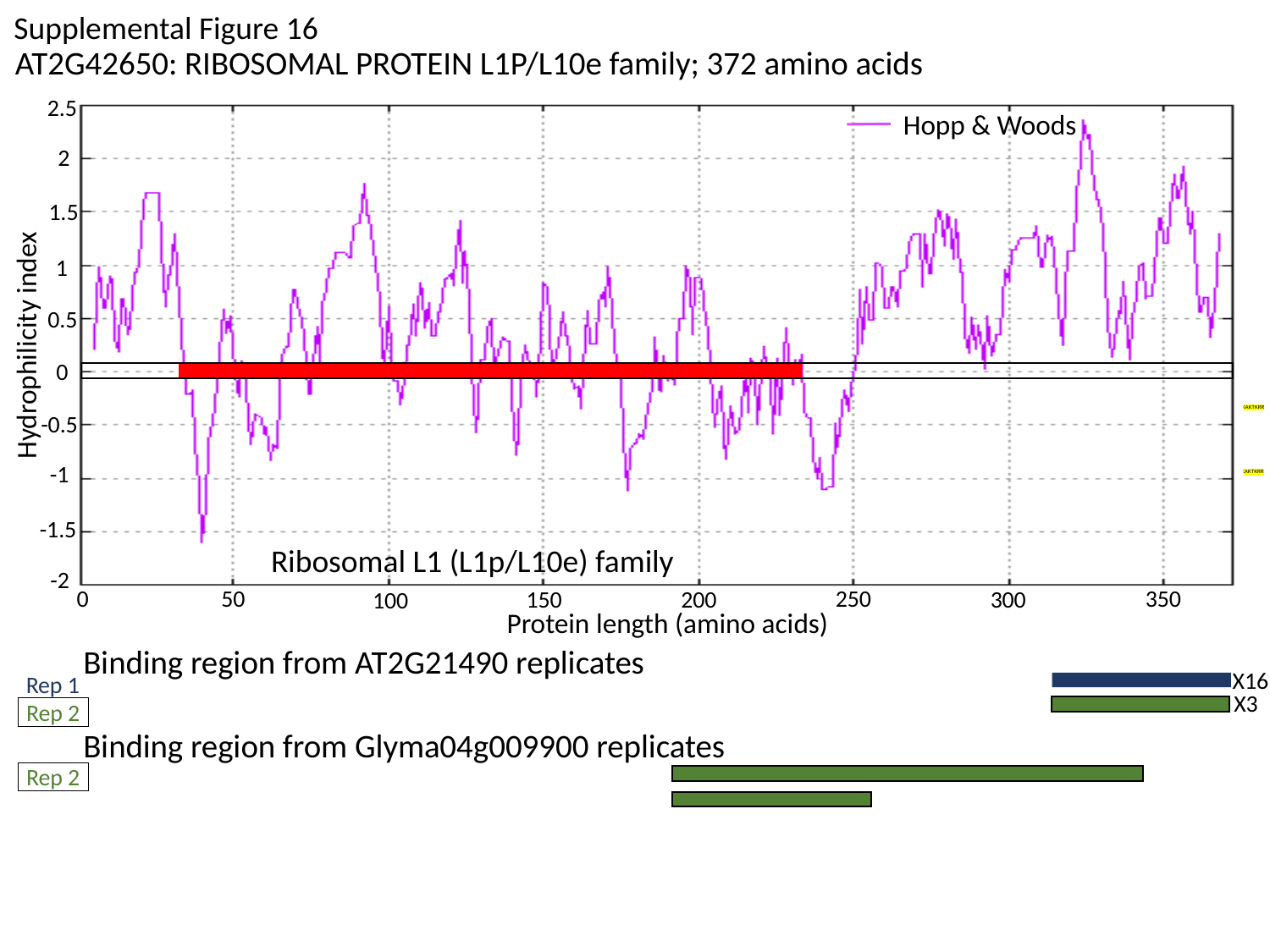

Supplemental Figure 16
AT2G42650: RIBOSOMAL PROTEIN L1P/L10e family; 372 amino acids
2.5
Hopp & Woods
2
1.5
1
0.5
Hydrophilicity index
0
MSRVSPKTVDDAVKALVKEGNEKSRTEKPQLLEEDGFFYLVVALKKIPQRNFTNAYRIPLPHPLINTTEDSPELCLIIDDRPESGLTEEDAKKNIKSENIPITKVVKLSKLKSDYGSFESKRKLCDSYDMFFSDRRVIPMLPKLIGKKFFQSKKTPVAIDLKHMNWKEQIEKACGAAMFFMRTGSCSAIKVAKLSMESDDIVENVTATLNGVVDVLPSRWKYIRSLHLKLSESLSLPLYQTVPYLQLKIDPLGVEEVKNGEGLAKSDVDDSSSKSVKTKKKNGKIHEVRYMDSNVSETLGDDEFDRSVGEDEVADDLNASGDKKKRKKMSSSKSAVSGKPDIVKSKNGQKSKKLKKDIDESGGGFKAKTKRR
-0.5
-1
MSRVSPKTVDDAVKALVKEGNEKSRTEKPQLLEEDGFFYLVVALKKIPQRNFTNAYRIPLPHPLINTTEDSPELCLIIDDRPESGLTEEDAKKNIKSENIPITKVVKLSKLKSDYGSFESKRKLCDSYDMFFSDRRVIPMLPKLIGKKFFQSKKTPVAIDLKHMNWKEQIEKACGAAMFFMRTGSCSAIKVAKLSMESDDIVENVTATLNGVVDVLPSRWKYIRSLHLKLSESLSLPLYQTVPYLQLKIDPLGVEEVKNGEGLAKSDVDDSSSKSVKTKKKNGKIHEVRYMDSNVSETLGDDEFDRSVGEDEVADDLNASGDKKKRKKMSSSKSAVSGKPDIVKSKNGQKSKKLKKDIDESGGGFKAKTKRR
-1.5
Ribosomal L1 (L1p/L10e) family
-2
0
50
250
350
200
300
150
100
Protein length (amino acids)
Binding region from AT2G21490 replicates
X16
Rep 1
X3
Rep 2
Binding region from Glyma04g009900 replicates
Rep 2

## Slide 17
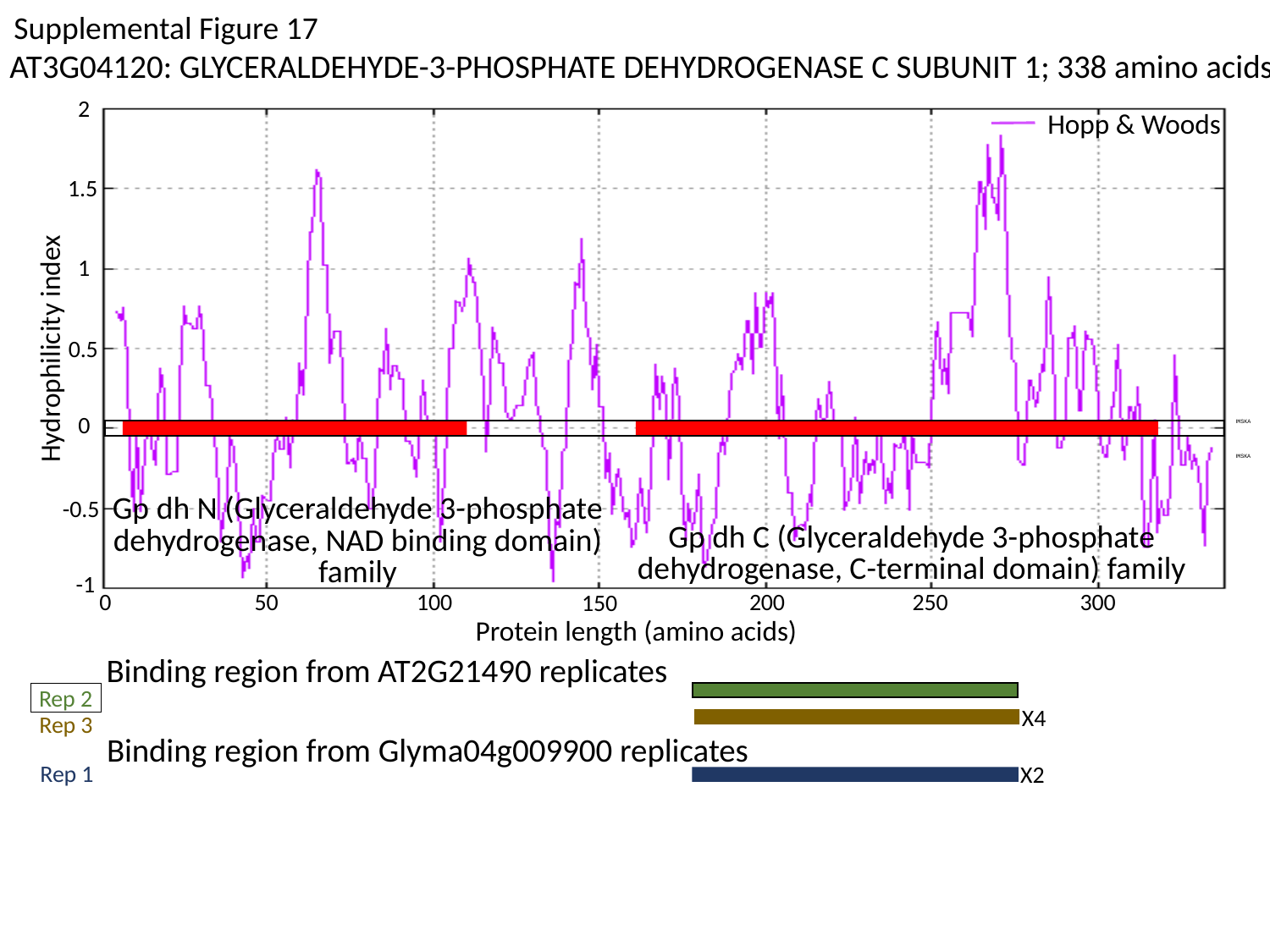

Supplemental Figure 17
AT3G04120: GLYCERALDEHYDE-3-PHOSPHATE DEHYDROGENASE C SUBUNIT 1; 338 amino acids
2
Hopp & Woods
1.5
1
0.5
Hydrophilicity index
0
MADKKIRIGINGFGRIGRLVARVVLQRDDVELVAVNDPFITTEYMTYMFKYDSVHGQWKHNELKIKDEKTLLFGEKPVTVFGIRNPEDIPWAEAGADYVVESTGVFTDKDKAAAHLKGGAKKVVISAPSKDAPMFVVGVNEHEYKSDLDIVSNASCTTNCLAPLAKVINDRFGIVEGLMTTVHSITATQKTVDGPSMKDWRGGRAASFNIIPSSTGAAKAVGKVLPALNGKLTGMSFRVPTVDVSVVDLTVRLEKAATYDEIKKAIKEESEGKLKGILGYTEDDVVSTDFVGDNRSSIFDAKAGIALSDKFVKLVSWYDNEWGYSSRVVDLIVHMSKA
MADKKIRIGINGFGRIGRLVARVVLQRDDVELVAVNDPFITTEYMTYMFKYDSVHGQWKHNELKIKDEKTLLFGEKPVTVFGIRNPEDIPWAEAGADYVVESTGVFTDKDKAAAHLKGGAKKVVISAPSKDAPMFVVGVNEHEYKSDLDIVSNASCTTNCLAPLAKVINDRFGIVEGLMTTVHSITATQKTVDGPSMKDWRGGRAASFNIIPSSTGAAKAVGKVLPALNGKLTGMSFRVPTVDVSVVDLTVRLEKAATYDEIKKAIKEESEGKLKGILGYTEDDVVSTDFVGDNRSSIFDAKAGIALSDKFVKLVSWYDNEWGYSSRVVDLIVHMSKA
-0.5
Gp dh N (Glyceraldehyde 3-phosphate dehydrogenase, NAD binding domain) family
Gp dh C (Glyceraldehyde 3-phosphate dehydrogenase, C-terminal domain) family
-1
50
200
100
0
250
300
150
Protein length (amino acids)
Binding region from AT2G21490 replicates
Rep 2
X4
Rep 3
Binding region from Glyma04g009900 replicates
Rep 1
X2

## Slide 18
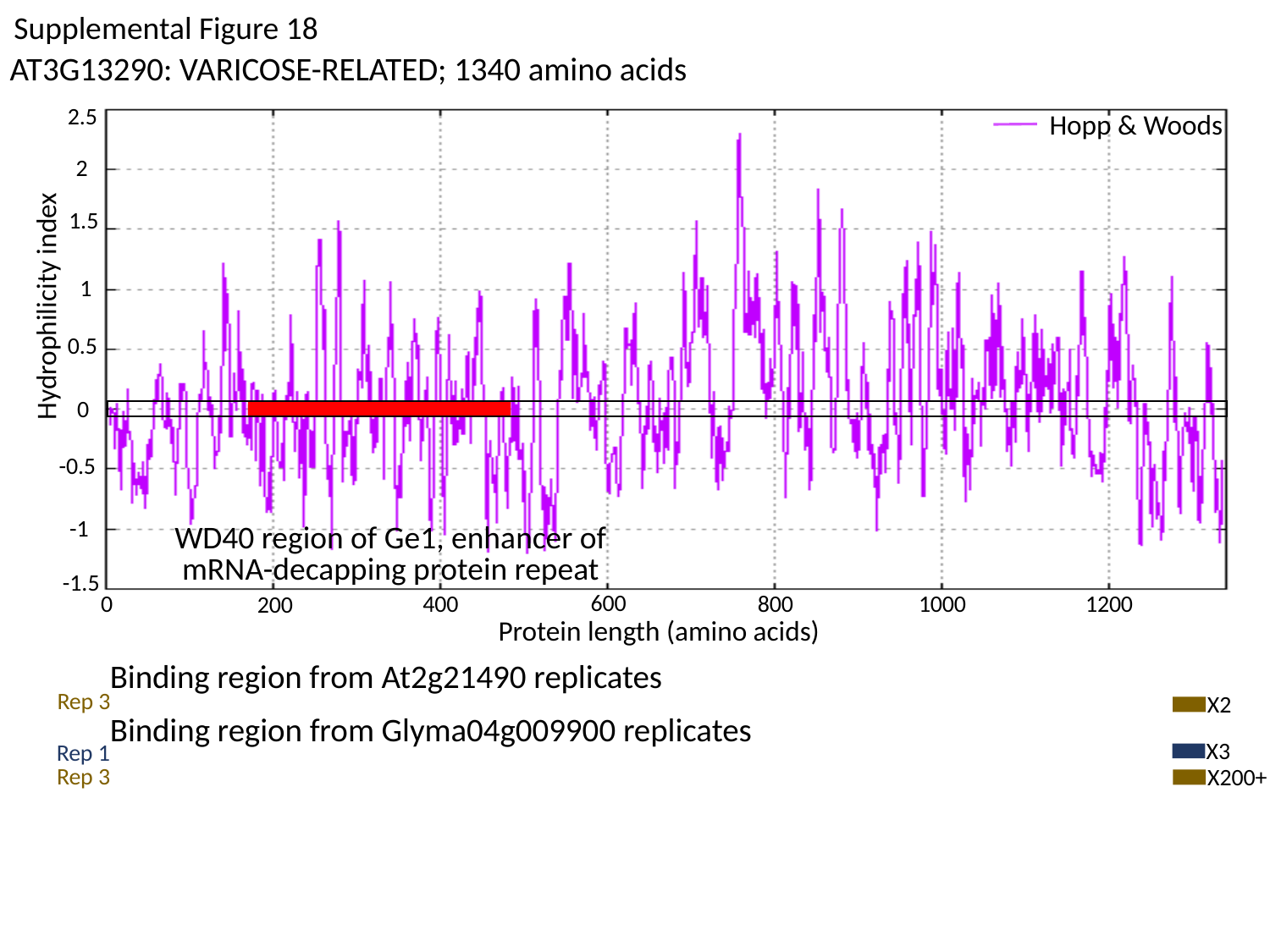

Supplemental Figure 18
AT3G13290: VARICOSE-RELATED; 1340 amino acids
2.5
Hopp & Woods
2
1.5
1
Hydrophilicity index
0.5
0
-0.5
-1
WD40 region of Ge1, enhancer of mRNA-decapping protein repeat
-1.5
600
1000
0
1200
800
400
200
Protein length (amino acids)
Binding region from At2g21490 replicates
Rep 3
X2
Binding region from Glyma04g009900 replicates
X3
Rep 1
Rep 3
X200+

## Slide 19
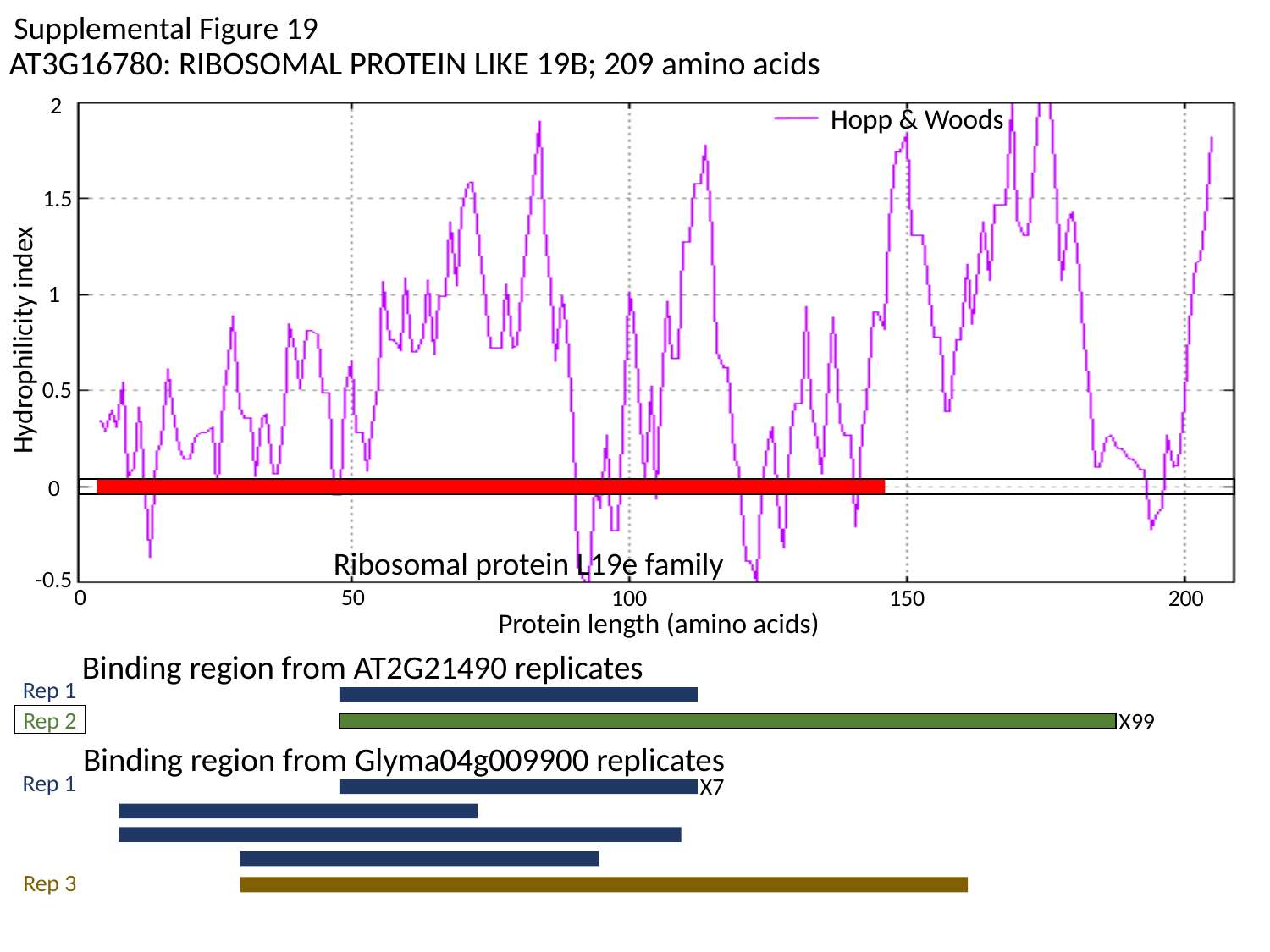

Supplemental Figure 19
AT3G16780: RIBOSOMAL PROTEIN LIKE 19B; 209 amino acids
2
Hopp & Woods
1.5
1
Hydrophilicity index
0.5
MVSLKIQKRLAASVMKCGKGKVWLDPNESGDISMANSRQNIRKLVKDGFIIRKPTKIHSRSRARALNEAKRKGRHSGYGKRKGTREARLPTKILWMRRMRVLRRFLSKYRESKKIDRHMYHDMYMKVKGNVFKNKRVLMESIHKMKAEKAREKTLADQFEAKRIKNKASRERKFARREERLAQGPGGGETTTPAGAPQQPEVTKKKSKK
0
MVSLKIQKRLAASVMKCGKGKVWLDPNESGDISMANSRQNIRKLVKDGFIIRKPTKIHSRSRARALNEAKRKGRHSGYGKRKGTREARLPTKILWMRRMRVLRRFLSKYRESKKIDRHMYHDMYMKVKGNVFKNKRVLMESIHKMKAEKAREKTLADQFEAKRIKNKASRERKFARREERLAQGPGGGETTTPAGAPQQPEVTKKKSKK
Ribosomal protein L19e family
-0.5
50
0
150
200
100
Protein length (amino acids)
Binding region from AT2G21490 replicates
Rep 1
X99
Rep 2
Binding region from Glyma04g009900 replicates
Rep 1
X7
Rep 3

## Slide 20
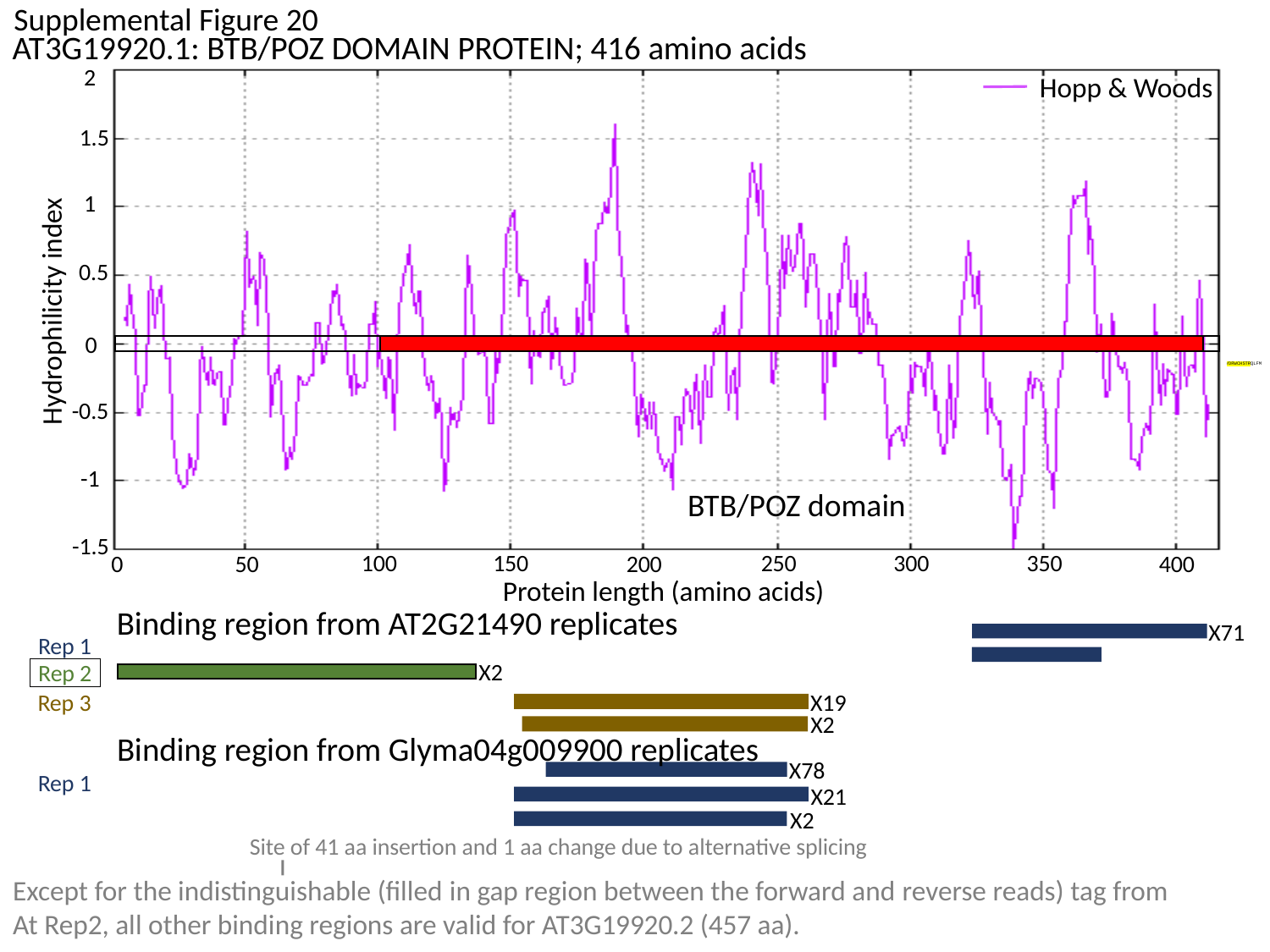

Supplemental Figure 20
AT3G19920.1: BTB/POZ DOMAIN PROTEIN; 416 amino acids
2
Hopp & Woods
1.5
1
0.5
Hydrophilicity index
0
MSSSRELQYLEYTYRNNRPTTGLLNSIFMTTVNTAARSLVSVASTASTPEIPSRRWSASDHLSFASGLLTTAAENALVPAKASSSSSTSSTALVKYSGSSDLGMMICDGVDEPSVNSLGRALCHALALMNEIPVTSRKYQFAMGMAEKIMEDNAQSGHVDLLDVNRAALASSFARTTARLQDCLKRSRTADEPFGGLPLRVVSALPLGGYVASYVRGLSACINTVRSLADMTGNLLSQTRRRESAVVRAGGIQENEAELAVEKLAEELLWMTEKLRRYGAVAEGIKRWSYASGLASLSLTAAPRVQGLMVKISALLIGELARDSTQVPGQVTFRLLANWLPLFSHARNGLAFPVLTGYERVEVERAIDKAISTLPALDQEILLTNWLQDFSVSASEWPNLQPAYDRWCHSTRQLFM
-0.5
-1
-1.5
100
300
350
150
250
200
400
50
0
Protein length (amino acids)
Binding region from AT2G21490 replicates
X71
Rep 1
X2
Rep 2
X19
Rep 3
X2
Binding region from Glyma04g009900 replicates
X78
Rep 1
X21
X2
BTB/POZ domain
Site of 41 aa insertion and 1 aa change due to alternative splicing
Except for the indistinguishable (filled in gap region between the forward and reverse reads) tag from
At Rep2, all other binding regions are valid for AT3G19920.2 (457 aa).

## Slide 21
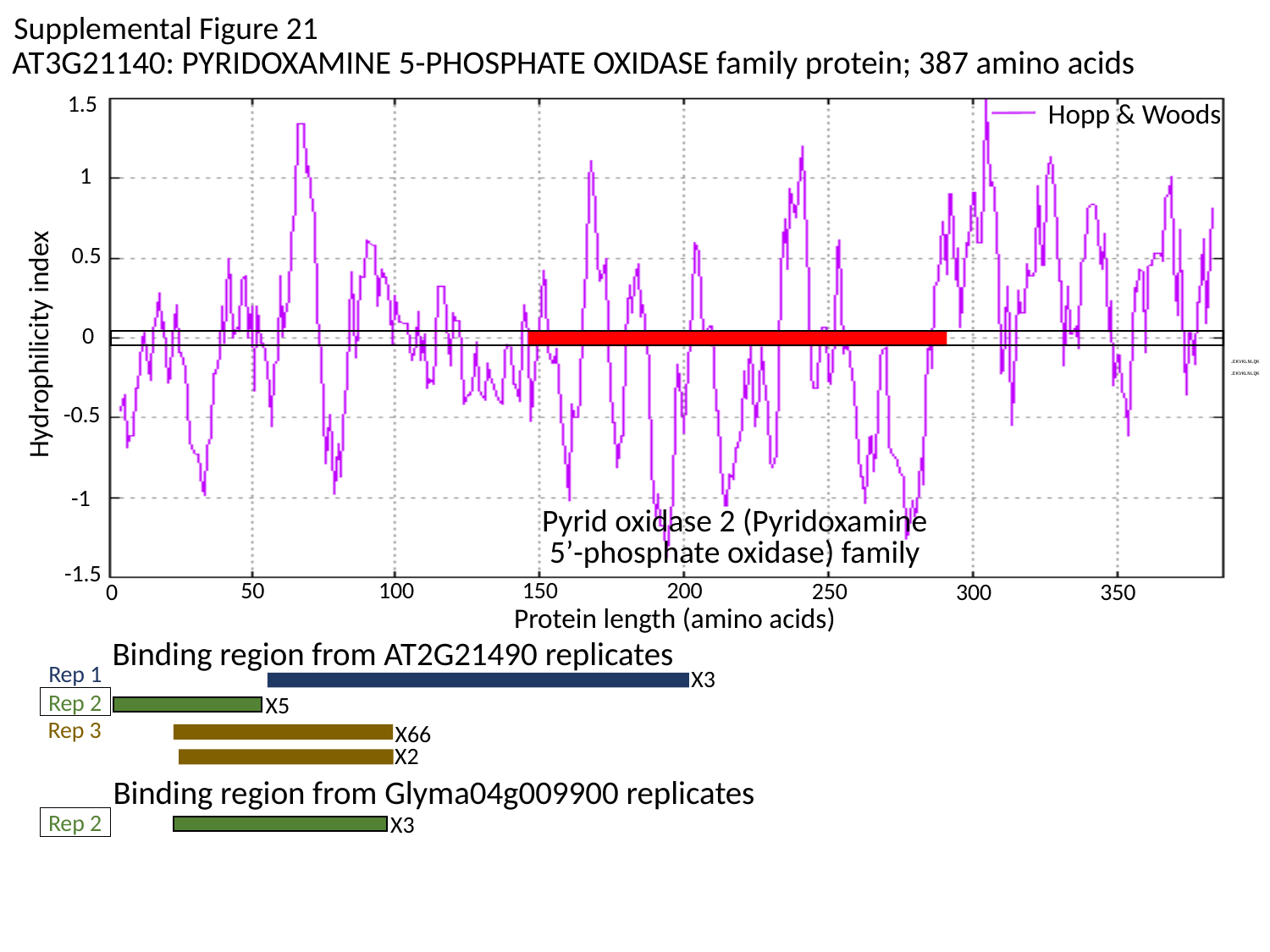

Supplemental Figure 21
AT3G21140: PYRIDOXAMINE 5-PHOSPHATE OXIDASE family protein; 387 amino acids
1.5
Hopp & Woods
1
0.5
0
Hydrophilicity index
MEALFTSTHTPNLQTKPLLKSPLPTSSQSSCWFCNSLPKTQFPKLRLSNGSSHGLRIQALLRNETPSEGEDNNGFGFFPGDIFSLSQEKLGSNSNGETSHSVIDVEASLAHPQGGGGNRAGLFRTPISGGVQNATSAHALPRPALAVRNLLEQARFAHLCTVMSKMHHRREGYPFGSLVDFAPDRMGHPIFLFSPLAIHTRNLLNEPRCSLVVQIPGWSGLSNARVTLFGDVYPLSEDEQEWAHKQYIAKHPHGPSEQWGNFHYFRMQNISDIYFIGGFGTVAWVDVKEYEGLQPDKIAVDGGERNLKELNAIFSKPLRELLSTESEVDDAALISIDSKGIDVRVRQGAQFNIQRLAFEEGHGVETLEEAKSALWKVLEKVKLNLQK
MEALFTSTHTPNLQTKPLLKSPLPTSSQSSCWFCNSLPKTQFPKLRLSNGSSHGLRIQALLRNETPSEGEDNNGFGFFPGDIFSLSQEKLGSNSNGETSHSVIDVEASLAHPQGGGGNRAGLFRTPISGGVQNATSAHALPRPALAVRNLLEQARFAHLCTVMSKMHHRREGYPFGSLVDFAPDRMGHPIFLFSPLAIHTRNLLNEPRCSLVVQIPGWSGLSNARVTLFGDVYPLSEDEQEWAHKQYIAKHPHGPSEQWGNFHYFRMQNISDIYFIGGFGTVAWVDVKEYEGLQPDKIAVDGGERNLKELNAIFSKPLRELLSTESEVDDAALISIDSKGIDVRVRQGAQFNIQRLAFEEGHGVETLEEAKSALWKVLEKVKLNLQK
-0.5
-1
Pyrid oxidase 2 (Pyridoxamine 5’-phosphate oxidase) family
-1.5
200
100
50
150
250
0
350
300
Protein length (amino acids)
Binding region from AT2G21490 replicates
Rep 1
X3
X5
Rep 2
Rep 3
X66
X2
Binding region from Glyma04g009900 replicates
X3
Rep 2

## Slide 22
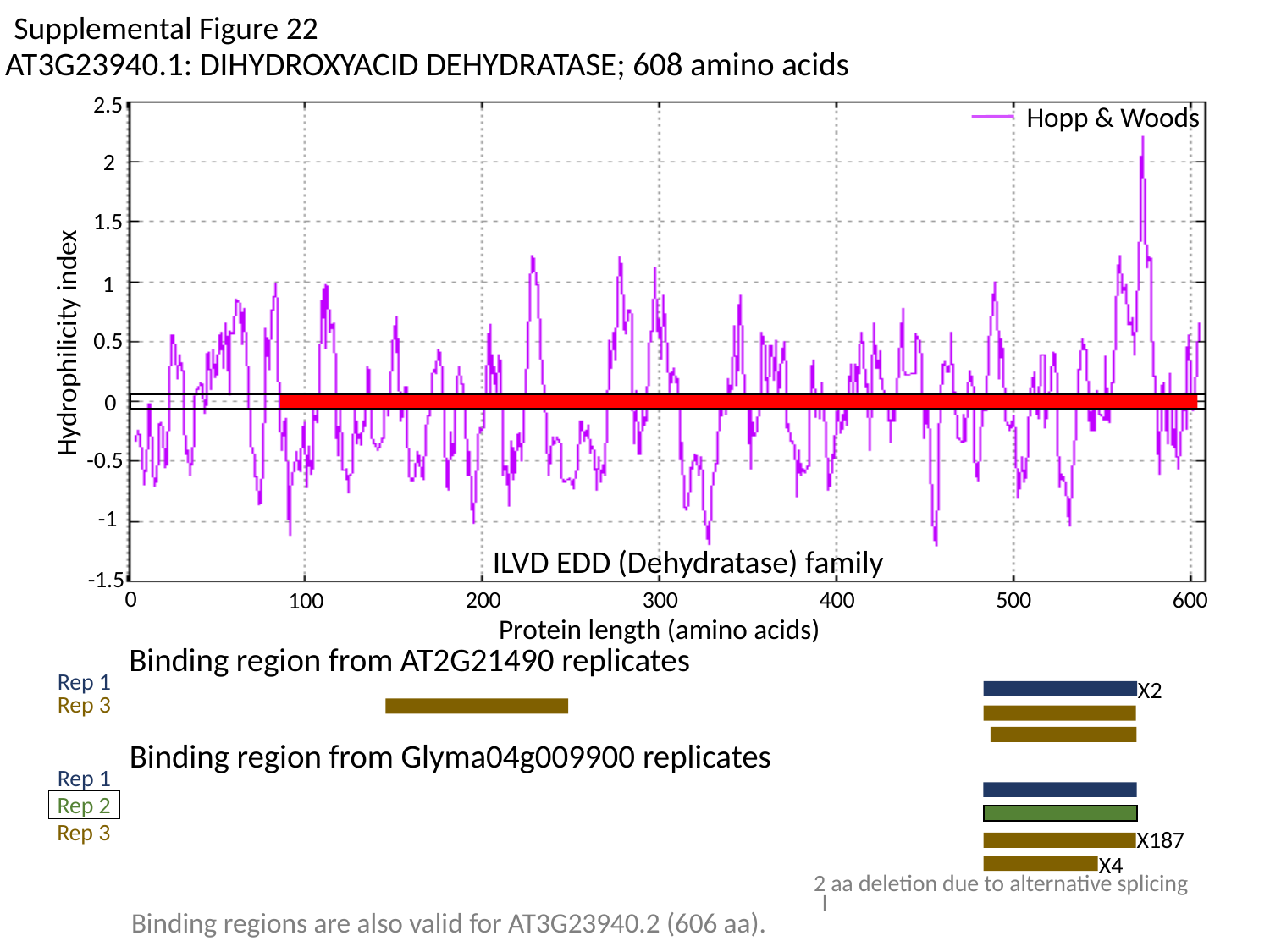

Supplemental Figure 22
AT3G23940.1: DIHYDROXYACID DEHYDRATASE; 608 amino acids
2.5
Hopp & Woods
2
1.5
1
0.5
Hydrophilicity index
MQATIFSPRATLFPCKPLLPSHNVNSRRPSIISCSAQSVTADPSPPITDTNKLNKYSSRITEPKSQGGSQAILHGVGLSDDDLLKPQIGISSVWYEGNTCNMHLLKLSEAVKEGVENAGMVGFRFNTIGVSDAISMGTRGMCFSLQSRDLIADSIETVMSAQWYDGNISIPGCDKNMPGTIMAMGRLNRPGIMVYGGTIKPGHFQDKTYDIVSAFQSYGEFVSGSISDEQRKTVLHHSCPGAGACGGMYTANTMASAIEAMGMSLPYSSSIPAEDPLKLDECRLAGKYLLELLKMDLKPRDIITPKSLRNAMVSVMALGGSTNAVLHLIAIARSVGLELTLDDFQKVSDAVPFLADLKPSGKYVMEDIHKIGGTPAVLRYLLELGLMDGDCMTVTGQTLAQNLENVPSLTEGQEIIRPLSNPIKETGHIQILRGDLAPDGSVAKITGKEGLYFSGPALVFEGEESMLAAISADPMSFKGTVVVIRGEGPKGGPGMPEMLTPTSAIMGAGLGKECALLTDGRFSGGSHGFVVGHICPEAQEGGPIGLIKNGDIITIDIGKKRIDTQVSPEEMNDRRKKWTAPAYKVNRGVLYKYIKNVQSASDGCVTDE
0
MQATIFSPRATLFPCKPLLPSHNVNSRRPSIISCSAQSVTADPSPPITDTNKLNKYSSRITEPKSQGGSQAILHGVGLSDDDLLKPQIGISSVWYEGNTCNMHLLKLSEAVKEGVENAGMVGFRFNTIGVSDAISMGTRGMCFSLQSRDLIADSIETVMSAQWYDGNISIPGCDKNMPGTIMAMGRLNRPGIMVYGGTIKPGHFQDKTYDIVSAFQSYGEFVSGSISDEQRKTVLHHSCPGAGACGGMYTANTMASAIEAMGMSLPYSSSIPAEDPLKLDECRLAGKYLLELLKMDLKPRDIITPKSLRNAMVSVMALGGSTNAVLHLIAIARSVGLELTLDDFQKVSDAVPFLADLKPSGKYVMEDIHKIGGTPAVLRYLLELGLMDGDCMTVTGQTLAQNLENVPSLTEGQEIIRPLSNPIKETGHIQILRGDLAPDGSVAKITGKEGLYFSGPALVFEGEESMLAAISADPMSFKGTVVVIRGEGPKGGPGMPEMLTPTSAIMGAGLGKECALLTDGRFSGGSHGFVVGHICPEAQEGGPIGLIKNGDIITIDIGKKRIDTQVSPEEMNDRRKKWTAPAYKVNRGVLYKYIKNVQSASDGCVTDE
-0.5
-1
ILVD EDD (Dehydratase) family
-1.5
0
500
400
200
300
600
100
Protein length (amino acids)
Binding region from AT2G21490 replicates
Rep 1
X2
Rep 3
Binding region from Glyma04g009900 replicates
Rep 1
Rep 2
Rep 3
X187
X4
2 aa deletion due to alternative splicing
Binding regions are also valid for AT3G23940.2 (606 aa).

## Slide 23
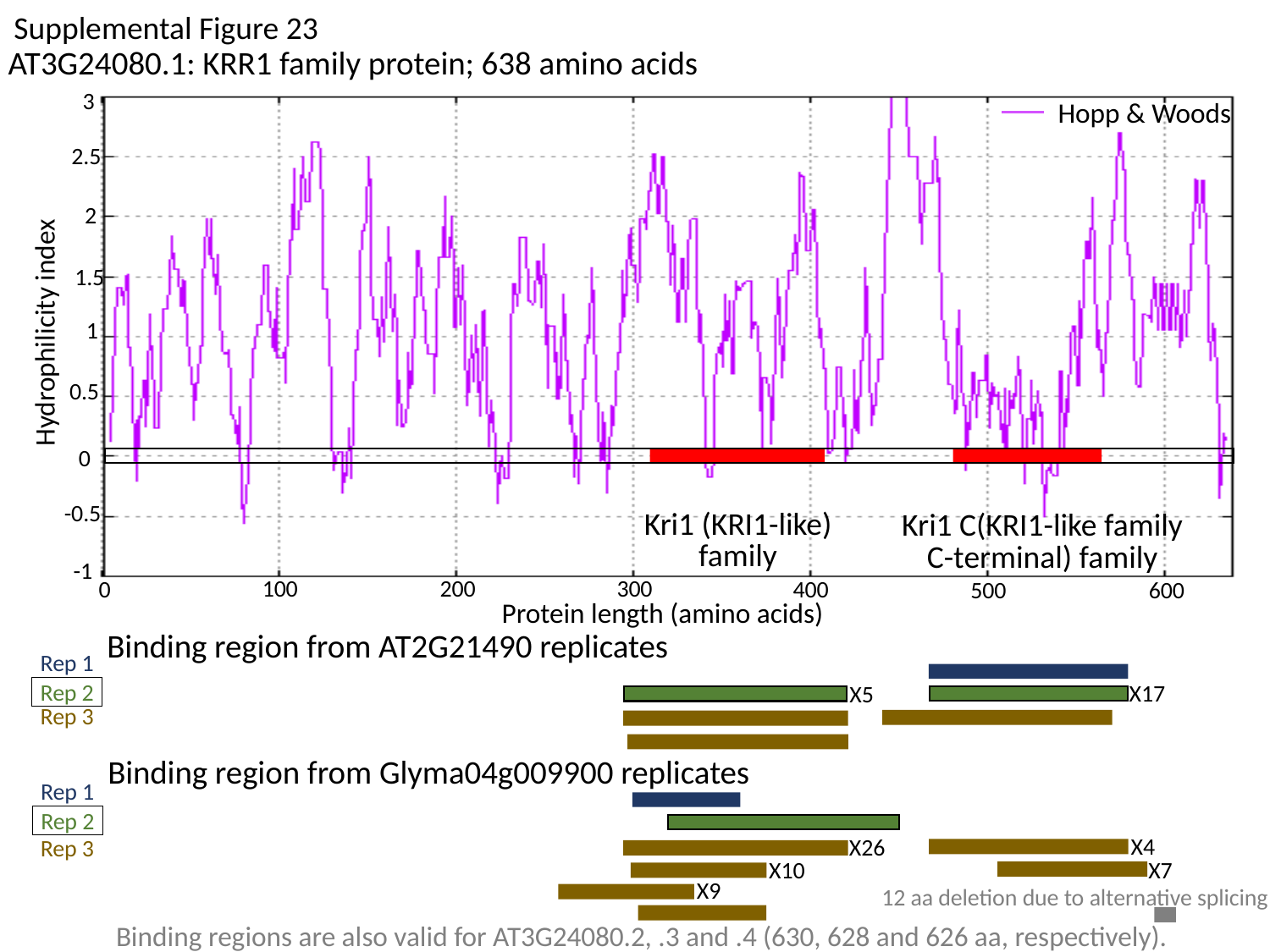

Supplemental Figure 23
AT3G24080.1: KRR1 family protein; 638 amino acids
3
Hopp & Woods
2.5
2
1.5
1
Hydrophilicity index
0.5
MMGRPKAIPDDDDGELSQFKGFDVDNDFAKRLLHNKEREDLQRYEEKNKQGLFSESEEEPETESEPDSDLGNPESNLKFVDLLIKVKKKDPIIKDKDAKFYEYDDESSEEDEVDKKDTKKKKKKKKMYLKDVQAQHLLEGGPEFVEEDEERKVRTYAEEQKETRKAVTDAWKAEGNESGEDDDFLRVVEKEGDDDVEVDEELAKKMDEYYGDEAEATENQFLKDYLVKQLWKEKEERVPGEEELKELSEDDHEVWDQEDFEAGRPLDLLNYRHEDENAGDTVMGQSRVVEGSVRKKDNARKAQRKNKDERMKKEDDVRKEELKRLKNVKKKEIKEKMKKVLSVAGFKDGEECPLDAKDFDDEFDPEEYDKMMKAAFDDKYYGEEDSDLNSDEDDDGEKPDFDKEDELLGLPKDWDVTKGGDVFAAAREKVLKHKENMLGIDEEEEEDEEEEEDEEEEEVDEEKEAEGKRKRKRKTSLVQKTKEALMEEYYKLDYEDTIGELRTRFKYAKVQPNRFELDTGEILTLDDAELNQYVPLKKMAPYVEKDWEVNRHKVKEQKRKIRELWEGKHDEKKSKKRKKNDVVETKPTPKEAEAEAEAEAEAEAEAEAEAKLSRKAKRRRRQAEKKLPPSRMAAYGKA
0
MMGRPKAIPDDDDGELSQFKGFDVDNDFAKRLLHNKEREDLQRYEEKNKQGLFSESEEEPETESEPDSDLGNPESNLKFVDLLIKVKKKDPIIKDKDAKFYEYDDESSEEDEVDKKDTKKKKKKKKMYLKDVQAQHLLEGGPEFVEEDEERKVRTYAEEQKETRKAVTDAWKAEGNESGEDDDFLRVVEKEGDDDVEVDEELAKKMDEYYGDEAEATENQFLKDYLVKQLWKEKEERVPGEEELKELSEDDHEVWDQEDFEAGRPLDLLNYRHEDENAGDTVMGQSRVVEGSVRKKDNARKAQRKNKDERMKKEDDVRKEELKRLKNVKKKEIKEKMKKVLSVAGFKDGEECPLDAKDFDDEFDPEEYDKMMKAAFDDKYYGEEDSDLNSDEDDDGEKPDFDKEDELLGLPKDWDVTKGGDVFAAAREKVLKHKENMLGIDEEEEEDEEEEEDEEEEEVDEEKEAEGKRKRKRKTSLVQKTKEALMEEYYKLDYEDTIGELRTRFKYAKVQPNRFELDTGEILTLDDAELNQYVPLKKMAPYVEKDWEVNRHKVKEQKRKIRELWEGKHDEKKSKKRKKNDVVETKPTPKEAEAEAEAEAEAEAEAEAEAKLSRKAKRRRRQAEKKLPPSRMAAYGKA
-0.5
Kri1 (KRI1-like) family
Kri1 C(KRI1-like family C-terminal) family
-1
100
300
200
400
0
500
600
Protein length (amino acids)
Binding region from AT2G21490 replicates
Rep 1
X17
X5
Rep 2
Rep 3
Binding region from Glyma04g009900 replicates
Rep 1
Rep 2
X4
X26
Rep 3
X10
X7
X9
12 aa deletion due to alternative splicing
Binding regions are also valid for AT3G24080.2, .3 and .4 (630, 628 and 626 aa, respectively).

## Slide 24
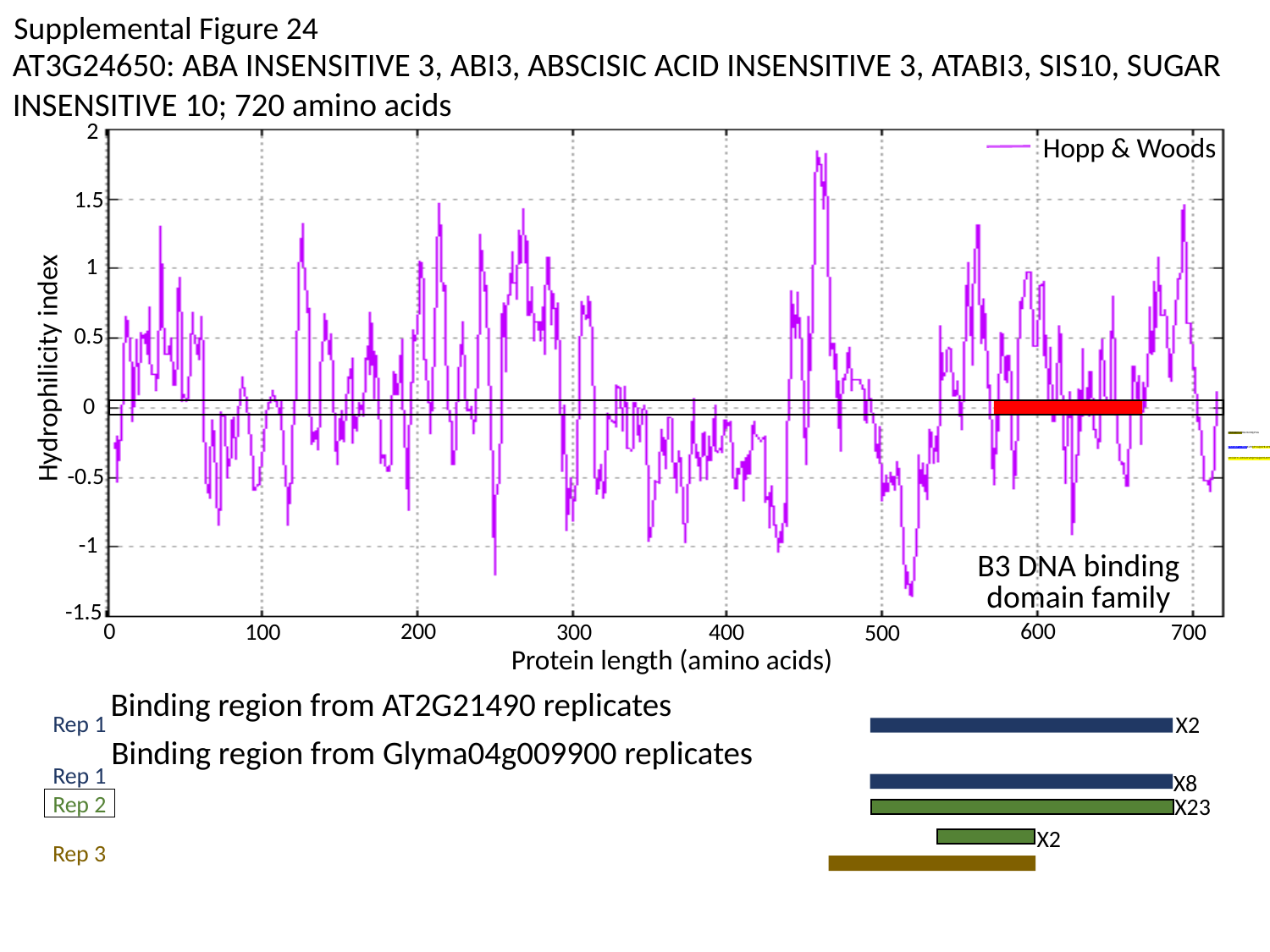

Supplemental Figure 24
AT3G24650: ABA INSENSITIVE 3, ABI3, ABSCISIC ACID INSENSITIVE 3, ATABI3, SIS10, SUGAR INSENSITIVE 10; 720 amino acids
2
Hopp & Woods
1.5
1
0.5
Hydrophilicity index
0
MKSLHVAANAGDLAEDCGILGGDADDTVLMDGIDEVGREIWLDDHGGDNNHVHGHQDDDLIVHHDPSIFYGDLPTLPDFPCMSSSSSSSTSPAPVNAIVSSASSSSAASSSTSSAASWAILRSDGEDPTPNQNQYASGNCDDSSGALQSTASMEIPLDSSQGFGCGEGGGDCIDMMETFGYMDLLDSNEFFDTSAIFSQDDDTQNPNLMDQTLERQEDQVVVPMMENNSGGDMQMMNSSLEQDDDLAAVFLEWLKNNKETVSAEDLRKVKIKKATIESAARRLGGGKEAMKQLLKLILEWVQTNHLQRRRTTTTTTNLSYQQSFQQDPFQNPNPNNNNLIPPSDQTCFSPSTWVPPPPQQQAFVSDPGFGYMPAPNYPPQPEFLPLLESPPSWPPPPQSGPMPHQQFPMPPTSQYNQFGDPTGFNGYNMNPYQYPYVPAGQMRDQRLLRLCSSATKEARKKRMARQRRFLSHHHRHNNNNNNNNNNQQNQTQIGETCAAVAPQLNPVATTATGGTWMYWPNVPAVPPQLPPVMETQLPTMDRAGSASAMPRQQVVPDRRQGWKPEKNLRFLLQKVLKQSDVGNLGRIVLPKKEAETHLPELEARDGISLAMEDIGTSRVWNMRYRFWPNNKSRMYLLENTGDFVKTNGLQEGDFIVIYSDVKCGKYLIRGVKVRQPSGQKPEAPPSSAATKRQNKSQRNINNNSPSANVVVASPTSQTVK
MKSLHVAANAGDLAEDCGILGGDADDTVLMDGIDEVGREIWLDDHGGDNNHVHGHQDDDLIVHHDPSIFYGDLPTLPDFPCMSSSSSSSTSPAPVNAIVSSASSSSAASSSTSSAASWAILRSDGEDPTPNQNQYASGNCDDSSGALQSTASMEIPLDSSQGFGCGEGGGDCIDMMETFGYMDLLDSNEFFDTSAIFSQDDDTQNPNLMDQTLERQEDQVVVPMMENNSGGDMQMMNSSLEQDDDLAAVFLEWLKNNKETVSAEDLRKVKIKKATIESAARRLGGGKEAMKQLLKLILEWVQTNHLQRRRTTTTTTNLSYQQSFQQDPFQNPNPNNNNLIPPSDQTCFSPSTWVPPPPQQQAFVSDPGFGYMPAPNYPPQPEFLPLLESPPSWPPPPQSGPMPHQQFPMPPTSQYNQFGDPTGFNGYNMNPYQYPYVPAGQMRDQRLLRLCSSATKEARKKRMARQRRFLSHHHRHNNNNNNNNNNQQNQTQIGETCAAVAPQLNPVATTATGGTWMYWPNVPAVPPQLPPVMETQLPTMDRAGSASAMPRQQVVPDRRQGWKPEKNLRFLLQKVLKQSDVGNLGRIVLPKKEAETHLPELEARDGISLAMEDIGTSRVWNMRYRFWPNNKSRMYLLENTGDFVKTNGLQEGDFIVIYSDVKCGKYLIRGVKVRQPSGQKPEAPPSSAATKRQNKSQRNINNNSPSANVVVASPTSQTVK
MKSLHVAANAGDLAEDCGILGGDADDTVLMDGIDEVGREIWLDDHGGDNNHVHGHQDDDLIVHHDPSIFYGDLPTLPDFPCMSSSSSSSTSPAPVNAIVSSASSSSAASSSTSSAASWAILRSDGEDPTPNQNQYASGNCDDSSGALQSTASMEIPLDSSQGFGCGEGGGDCIDMMETFGYMDLLDSNEFFDTSAIFSQDDDTQNPNLMDQTLERQEDQVVVPMMENNSGGDMQMMNSSLEQDDDLAAVFLEWLKNNKETVSAEDLRKVKIKKATIESAARRLGGGKEAMKQLLKLILEWVQTNHLQRRRTTTTTTNLSYQQSFQQDPFQNPNPNNNNLIPPSDQTCFSPSTWVPPPPQQQAFVSDPGFGYMPAPNYPPQPEFLPLLESPPSWPPPPQSGPMPHQQFPMPPTSQYNQFGDPTGFNGYNMNPYQYPYVPAGQMRDQRLLRLCSSATKEARKKRMARQRRFLSHHHRHNNNNNNNNNNQQNQTQIGETCAAVAPQLNPVATTATGGTWMYWPNVPAVPPQLPPVMETQLPTMDRAGSASAMPRQQVVPDRRQGWKPEKNLRFLLQKVLKQSDVGNLGRIVLPKKEAETHLPELEARDGISLAMEDIGTSRVWNMRYRFWPNNKSRMYLLENTGDFVKTNGLQEGDFIVIYSDVKCGKYLIRGVKVRQPSGQKPEAPPSSAATKRQNKSQRNINNNSPSANVVVASPTSQTVK
-0.5
-1
B3 DNA binding domain family
-1.5
600
200
0
700
400
100
300
500
Protein length (amino acids)
Binding region from AT2G21490 replicates
Rep 1
X2
Binding region from Glyma04g009900 replicates
Rep 1
X8
X23
Rep 2
X2
Rep 3

## Slide 25
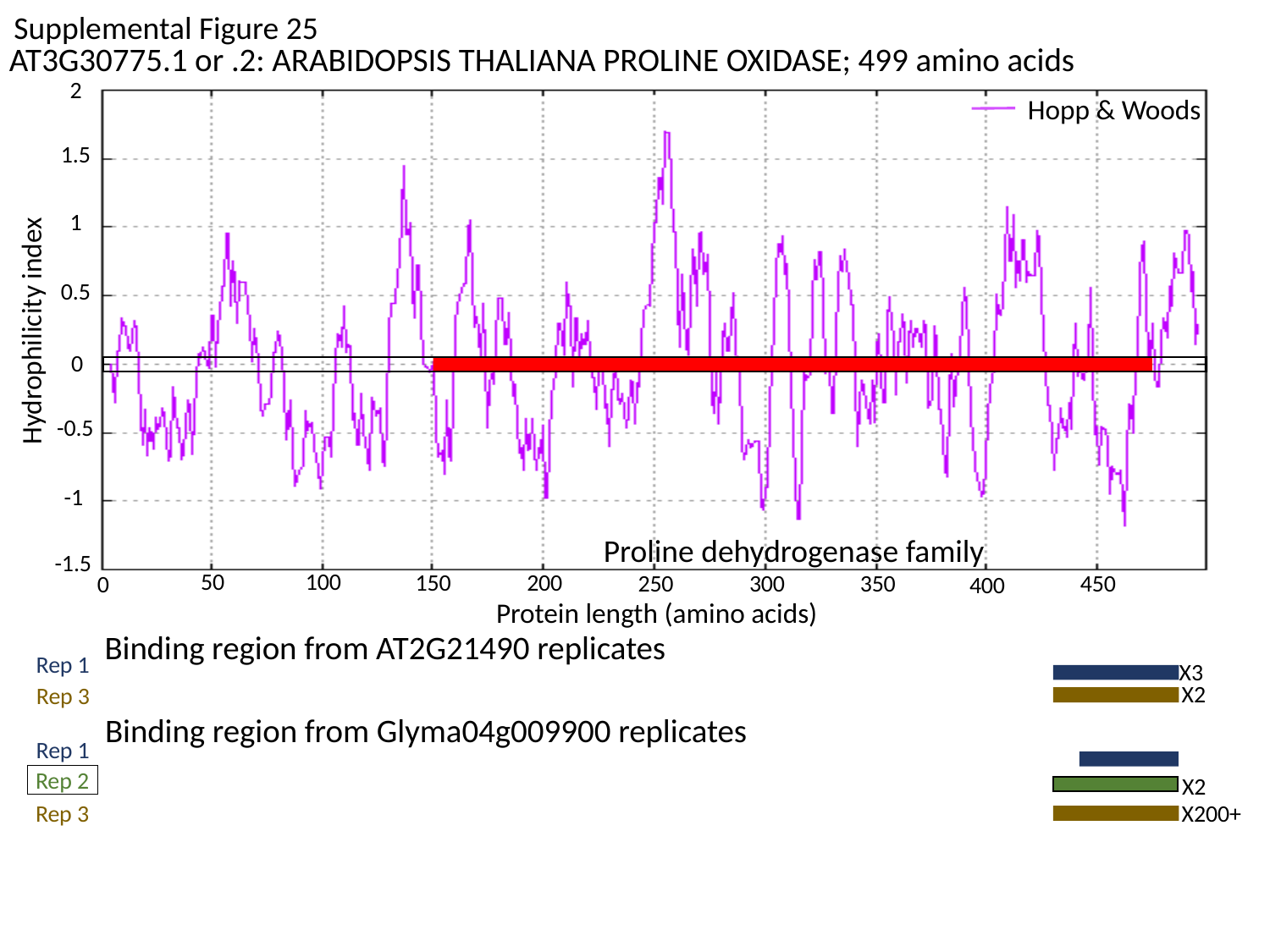

Supplemental Figure 25
AT3G30775.1 or .2: ARABIDOPSIS THALIANA PROLINE OXIDASE; 499 amino acids
2
Hopp & Woods
1.5
1
0.5
Hydrophilicity index
MATRLLRTNFIRRSYRLPAFSPVGPPTVTASTAVVPEILSFGQQAPEPPLHHPKPTEQSHDGLDLSDQARLFSSIPTSDLLRSTAVLHAAAIGPMVDLGTWVMSSKLMDASVTRGMVLGLVKSTFYDHFCAGEDADAAAERVRSVYEATGLKGMLVYGVEHADDAVSCDDNMQQFIRTIEAAKSLPTSHFSSVVVKITAICPISLLKRVSDLLRWEYKSPNFKLSWKLKSFPVFSESSPLYHTNSEPEPLTAEEERELEAAHGRIQEICRKCQESNVPLLIDAEDTILQPAIDYMAYSSAIMFNADKDRPIVYNTIQAYLRDAGERLHLAVQNAEKENVPMGFKLVRGAYMSSEASLADSLGCKSPVHDTIQDTHSCYNDCMTFLMEKASNGSGFGVVLATHNADSGRLASRKASDLGIDKQNGKIEFAQLYGMSDALSFGLKRAGFNVSKYMPFGPVATAIPYLLRRAYENRGMMATGAHDRQLMRMELKRRLIAGIA
MATRLLRTNFIRRSYRLPAFSPVGPPTVTASTAVVPEILSFGQQAPEPPLHHPKPTEQSHDGLDLSDQARLFSSIPTSDLLRSTAVLHAAAIGPMVDLGTWVMSSKLMDASVTRGMVLGLVKSTFYDHFCAGEDADAAAERVRSVYEATGLKGMLVYGVEHADDAVSCDDNMQQFIRTIEAAKSLPTSHFSSVVVKITAICPISLLKRVSDLLRWEYKSPNFKLSWKLKSFPVFSESSPLYHTNSEPEPLTAEEERELEAAHGRIQEICRKCQESNVPLLIDAEDTILQPAIDYMAYSSAIMFNADKDRPIVYNTIQAYLRDAGERLHLAVQNAEKENVPMGFKLVRGAYMSSEASLADSLGCKSPVHDTIQDTHSCYNDCMTFLMEKASNGSGFGVVLATHNADSGRLASRKASDLGIDKQNGKIEFAQLYGMSDALSFGLKRAGFNVSKYMPFGPVATAIPYLLRRAYENRGMMATGAHDRQLMRMELKRRLIAGIA
0
-0.5
-1
Proline dehydrogenase family
-1.5
50
100
200
150
450
350
250
300
0
400
Protein length (amino acids)
Binding region from AT2G21490 replicates
Rep 1
X3
X2
Rep 3
Binding region from Glyma04g009900 replicates
Rep 1
Rep 2
X2
Rep 3
X200+

## Slide 26
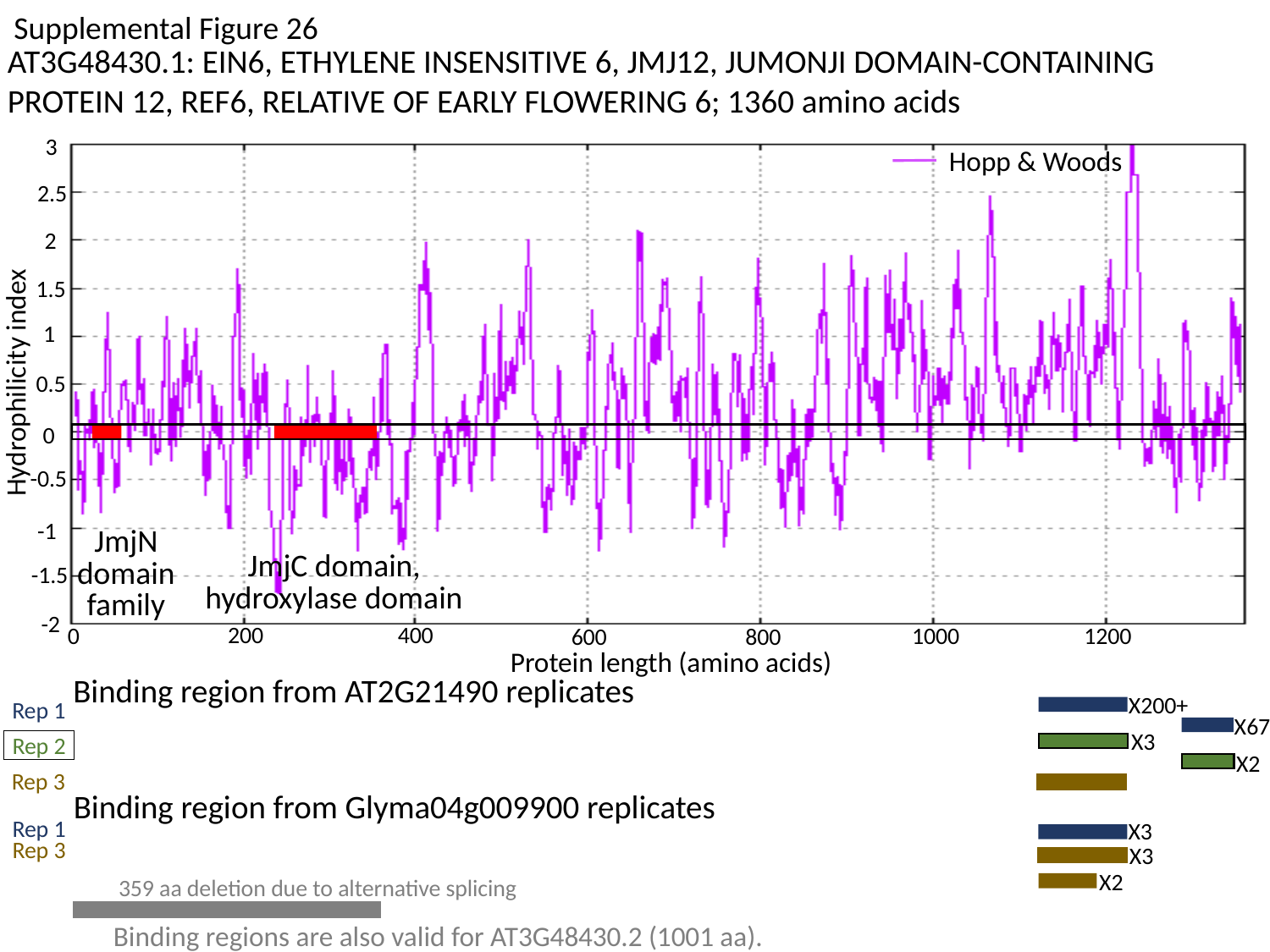

Supplemental Figure 26
AT3G48430.1: EIN6, ETHYLENE INSENSITIVE 6, JMJ12, JUMONJI DOMAIN-CONTAINING PROTEIN 12, REF6, RELATIVE OF EARLY FLOWERING 6; 1360 amino acids
3
Hopp & Woods
2.5
2
1.5
1
0.5
Hydrophilicity index
0
-0.5
-1
JmjN domain family
JmjC domain, hydroxylase domain
-1.5
-2
400
200
1200
0
1000
800
600
Protein length (amino acids)
Binding region from AT2G21490 replicates
X200+
Rep 1
X67
X3
Rep 2
X2
Rep 3
Binding region from Glyma04g009900 replicates
Rep 1
X3
Rep 3
X3
X2
MAVSEQSQDVFPWLKSLPVAPEFRPTLAEFQDPIAYILKIEEEASRYGICKILPPLPPPSKKTSISNLNRSLAARAAARVRDGGFGACDYDGGPTFATRQQQIGFCPRKQRPVQRPVWQSGEEYSFGEFEFKAKNFEKNYLKKCGKKSQLSALEIETLYWRATVDKPFSVEYANDMPGSAFIPLSLAAARRRESGGEGGTVGETAWNMRAMSRAEGSLLKFMKEEIPGVTSPMVYVAMMFSWFAWHVEDHDLHSLNYLHMGAGKTWYGVPKDAALAFEEVVRVHGYGEELNPLVTFSTLGEKTTVMSPEVFVKAGIPCCRLVQNPGEFVVTFPGAYHSGFSHGFNFGEASNIATPEWLRMAKDAAIRRAAINYPPMVSHLQLLYDFVLALGSRVPTSINPKPRSSRLKDKARSEGERLTKKLFVQNIIHNNELLSSLGKGSPVALLPQSSSDISVCSDLRIGSHLITNQENPIQLKCEDLSSDSVVVDLSNGLKDTVSVKEKFTSLCERSRNHLASTEKDTQETLSDAERRKNDAAVALSDQRLFSCVTCGVLSFDCVAIVQPKEAAARYLMSADCSFFNDWTAASGSANLGQAARSLHPQSKEKHDVNYFYNVPVQTMDHSVKTGDQKTSTTSPTIAHKDNDVLGMLASAYGDSSDSEEEDQKGLVTPSSKGETKTYDQEGSDGHEEARDGRTSDFNCQRLTSEQNGLSKGGKSSLLEIALPFIPRSDDDSCRLHVFCLEHAAEVEQQLRPFGGINLMLLCHPEYPRIEAEAKIVAEELVINHEWNDTEFRNVTREDEETIQAALDNVEAKGGNSDWTVKLGVNLSYSAILSRSPLYSKQMPYNSIIYKAFGRSSPVASSPSKPKVSGKRSSRQRKYVVGKWCGKVWMSHQVHPFLLEQDLEGEESERSCHLRVAMDEDATGKRSFPNNVSRDSTTMFGRKYCRKRKIRAKAVPRKKLTSFKREDGVSDDTSEDHSYKQQWRASGNEEESYFETGNTASGDSSNQMSDPHKGIIRHKGYKEFESDDEVSDRSLGEEYTVRACAASESSMENGSQHSMYDHDDDDDDIDRQPRGIPRSQQTRVFRNPVSYESEDNGVYQQSGRISISNRQANRMVGEYDSAENSLEERGFCSTGKRQTRSTAKRIAKTKTVQSSRDTKGRFLQEFASGKKNEELDSYMEGPSTRLRVRHQKPSRGSLETKPKKIGKKRSGNASFSRVATEKDVEEKEEEEEEEENEEEECAAYQCNMEGCTMSFSSEKQLMLHKRNICPIKGCGKNFFSHKYLVQHQRVHSDDRPLKCPWKGCKMTFKWAWSRTEHIRVHTGARPYVCAEPDCGQTFRFVSDFSRHKRKTGHSVKKTNKR
359 aa deletion due to alternative splicing
Binding regions are also valid for AT3G48430.2 (1001 aa).

## Slide 27
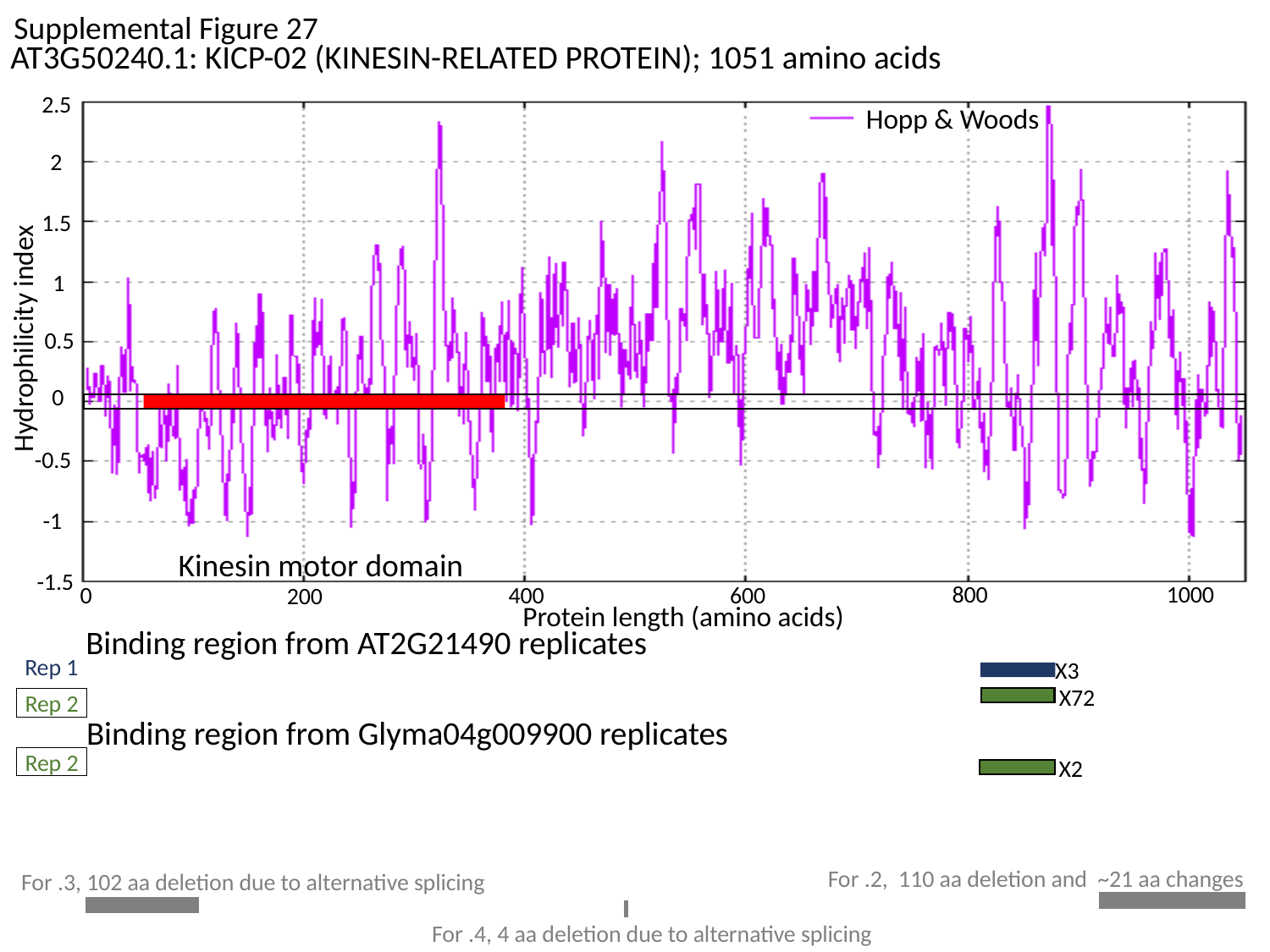

Supplemental Figure 27
AT3G50240.1: KICP-02 (KINESIN-RELATED PROTEIN); 1051 amino acids
2.5
Hopp & Woods
2
1.5
1
0.5
Hydrophilicity index
0
MESHSSLSSSSSSSPPSSLSSESCCVKVAVNVRPLIGDEVTQGCRECVSVSPVTPQVQMGTHPFTFDHVYGSNGSPSSLMFEECVAPLVDGLFHGYNATVLAYGQTGSGKTYTMGTGIKDGTKNGLIPQVMSALFNKIDSVKHQMGFQLHVSFIEILKEEVLDLLDSSVPFNRLANGTPGKVVLSKSPVQIRESPNGVITLSGATEVPIATKEEMASCLEQGSLTRATGSTNMNNESSRSHAIFTITLEQMRKISSISVVKDTVDEDMGEEYCCAKLHLVDLAGSERAKRTGSGGVRLKEGIHINRGLLALGNVISALGDEKRRKEGAHVPYRDSKLTRLLQDSLGGNSKTVMIACISPADINAEETLNTLKYANRARNIQNKPVANKDLICSEMQKMRQELQYLQATLCARGATSSEEVQVMREKIMKLESANEELSRELHIYRSKRVTLDYCNIDAQEDGVIFSKDDGLKRGFESMDSDYEMSEATSGGISEDIGAAEEWEHALRQNSMGKELNELSKRLEEKESEMRVCGIGTETIRQHFEKKMMELEKEKRTVQDERDMLLAEVEELAASSDRQAQVARDNHAHKLKALETQILNLKKKQENQVEVLKQKQKSEDAAKRLKTEIQCIKAQKVQLQQKMKQEAEQFRQWKASQEKELLQLKKEGRKTEHERLKLEALNRRQKMVLQRKTEEAAMATKRLKELLEARKSSPHDISVIANGQPPSRQTNEKSLRKWLDNELEVMAKVHQVRFQYEKQIQVRAALAVELTSLRQEMEFPSNSHQEKNGQFRFLSPNTRLERIASLESMLDVSSNALTAMGSQLSEAEEREHSLHAKPRWNHIQSMTDAKYLLQYVFDSTAEARSKIWEKDRDIKEKKEQLNDLLCLLQLTEVQNREILKEKKTREQTVSIALASTSSSYSGSSRSSSKHYGDNNASDDPSSPSSTYHRATKHLKYTGPGIVNISVRESEALLEETRKMKAMKKMGQSGKLWKWKRSHHQWLLQFKWKWQKPWKLSEWIKQNDETTMHVMSKSHHDDEDDHSWNRHSMFQGA
-0.5
MESHSSLSSSSSSSPPSSLSSESCCVKVAVNVRPLIGDEVTQGCRECVSVSPVTPQVQMGTHPFTFDHVYGSNGSPSSLMFEECVAPLVDGLFHGYNATVLAYGQTGSGKTYTMGTGIKDGTKNGLIPQVMSALFNKIDSVKHQMGFQLHVSFIEILKEEVLDLLDSSVPFNRLANGTPGKVVLSKSPVQIRESPNGVITLSGATEVPIATKEEMASCLEQGSLTRATGSTNMNNESSRSHAIFTITLEQMRKISSISVVKDTVDEDMGEEYCCAKLHLVDLAGSERAKRTGSGGVRLKEGIHINRGLLALGNVISALGDEKRRKEGAHVPYRDSKLTRLLQDSLGGNSKTVMIACISPADINAEETLNTLKYANRARNIQNKPVANKDLICSEMQKMRQELQYLQATLCARGATSSEEVQVMREKIMKLESANEELSRELHIYRSKRVTLDYCNIDAQEDGVIFSKDDGLKRGFESMDSDYEMSEATSGGISEDIGAAEEWEHALRQNSMGKELNELSKRLEEKESEMRVCGIGTETIRQHFEKKMMELEKEKRTVQDERDMLLAEVEELAASSDRQAQVARDNHAHKLKALETQILNLKKKQENQVEVLKQKQKSEDAAKRLKTEIQCIKAQKVQLQQKMKQEAEQFRQWKASQEKELLQLKKEGRKTEHERLKLEALNRRQKMVLQRKTEEAAMATKRLKELLEARKSSPHDISVIANGQPPSRQTNEKSLRKWLDNELEVMAKVHQVRFQYEKQIQVRAALAVELTSLRQEMEFPSNSHQEKNGQFRFLSPNTRLERIASLESMLDVSSNALTAMGSQLSEAEEREHSLHAKPRWNHIQSMTDAKYLLQYVFDSTAEARSKIWEKDRDIKEKKEQLNDLLCLLQLTEVQNREILKEKKTREQTVSIALASTSSSYSGSSRSSSKHYGDNNASDDPSSPSSTYHRATKHLKYTGPGIVNISVRESEALLEETRKMKAMKKMGQSGKLWKWKRSHHQWLLQFKWKWQKPWKLSEWIKQNDETTMHVMSKSHHDDEDDHSWNRHSMFQGA
-1
Kinesin motor domain
-1.5
800
1000
0
600
400
200
Protein length (amino acids)
Binding region from AT2G21490 replicates
Rep 1
X3
X72
Rep 2
Binding region from Glyma04g009900 replicates
X2
Rep 2
For .2, 110 aa deletion and ~21 aa changes
For .3, 102 aa deletion due to alternative splicing
For .4, 4 aa deletion due to alternative splicing

## Slide 28
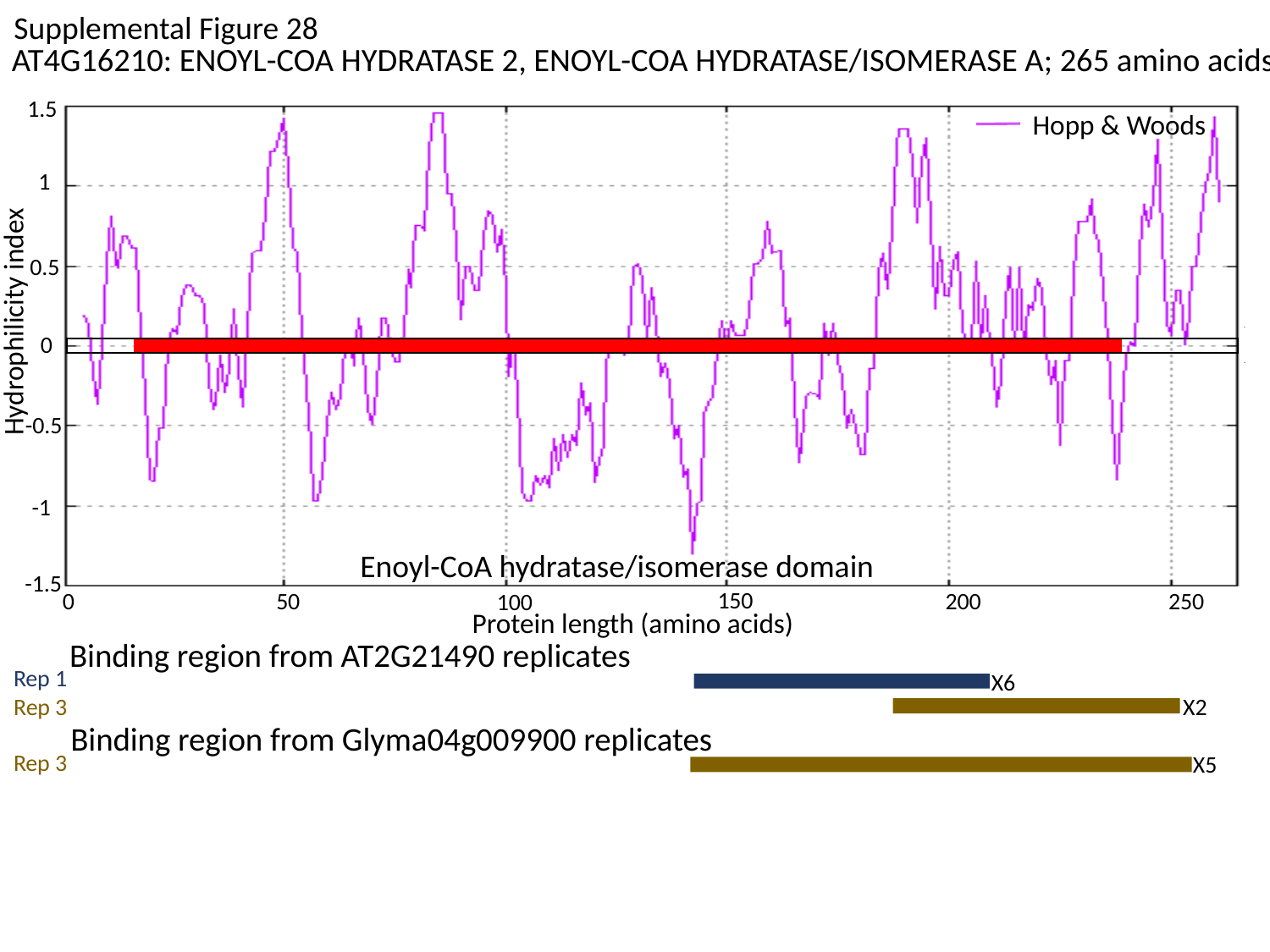

Supplemental Figure 28
AT4G16210: ENOYL-COA HYDRATASE 2, ENOYL-COA HYDRATASE/ISOMERASE A; 265 amino acids
1.5
Hopp & Woods
1
0.5
Hydrophilicity index
MDQTVSENLIQVKKESGGIAVITINRPKSLNSLTRAMMVDLAKAFKDMDSDESVQVVIFTGSGRSFCSGVDLTAAESVFKGDVKDPETDPVVQMERLRKPIIGAINGFAITAGFELALACDILVASRGAKFMDTHARFGIFPSWGLSQKLSRIIGANKAREVSLTSMPLTADVAGKLGFVNHVVEEGEALKKAREIAEAIIKNEQGMVLRIKSVINDGLKLDLGHALTLEKERAHAYYSGMTKEQFRKMQEFIAGRGSKKPSSKL
0
MDQTVSENLIQVKKESGGIAVITINRPKSLNSLTRAMMVDLAKAFKDMDSDESVQVVIFTGSGRSFCSGVDLTAAESVFKGDVKDPETDPVVQMERLRKPIIGAINGFAITAGFELALACDILVASRGAKFMDTHARFGIFPSWGLSQKLSRIIGANKAREVSLTSMPLTADVAGKLGFVNHVVEEGEALKKAREIAEAIIKNEQGMVLRIKSVINDGLKLDLGHALTLEKERAHAYYSGMTKEQFRKMQEFIAGRGSKKPSSKL
-0.5
-1
Enoyl-CoA hydratase/isomerase domain
-1.5
150
50
200
250
0
100
Protein length (amino acids)
Binding region from AT2G21490 replicates
Rep 1
X6
X2
Rep 3
Binding region from Glyma04g009900 replicates
Rep 3
X5

## Slide 29
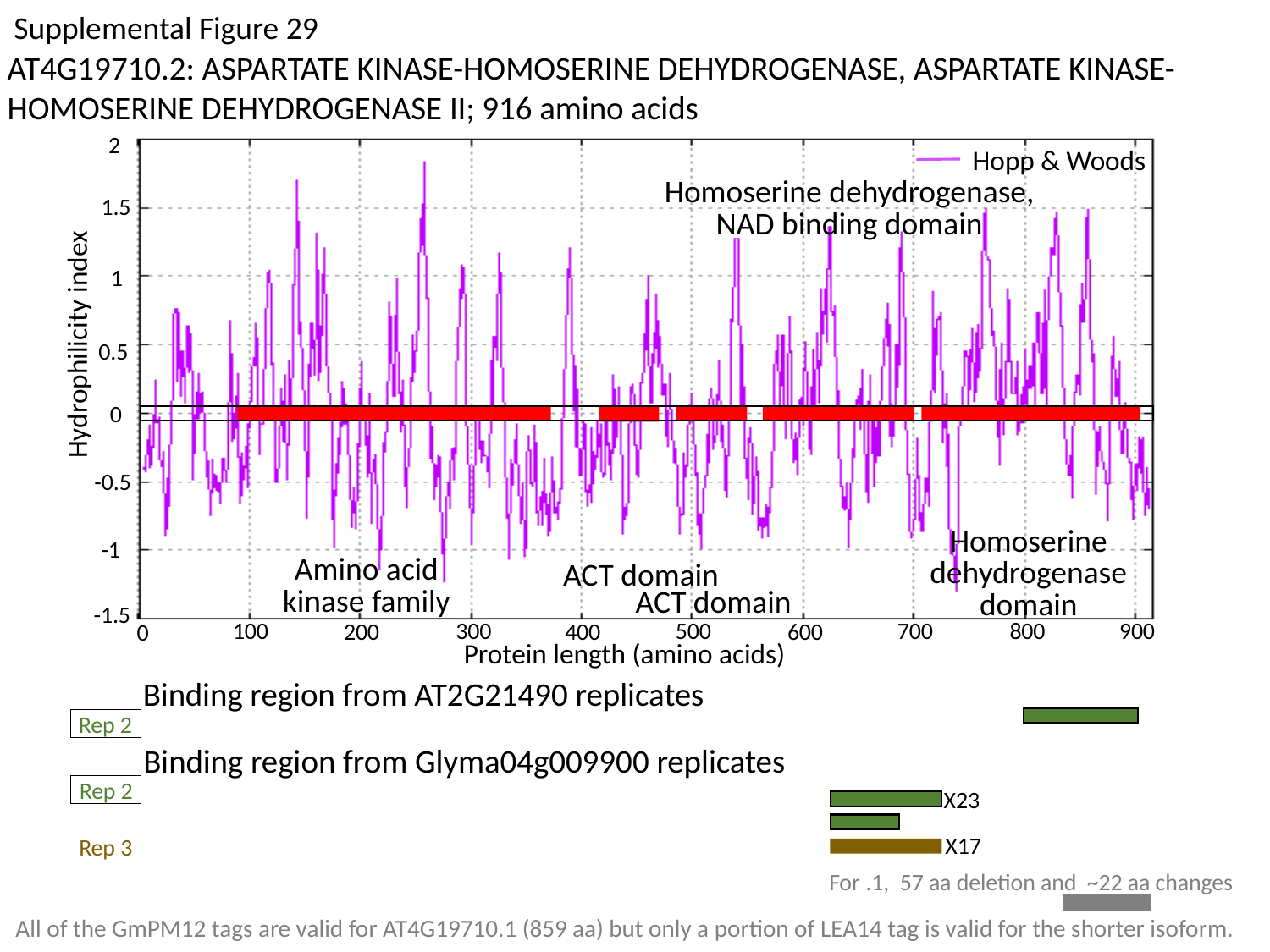

Supplemental Figure 29
AT4G19710.2: ASPARTATE KINASE-HOMOSERINE DEHYDROGENASE, ASPARTATE KINASE-HOMOSERINE DEHYDROGENASE II; 916 amino acids
2
Hopp & Woods
Homoserine dehydrogenase, NAD binding domain
1.5
1
Hydrophilicity index
0.5
0
MATLKPSFTVSPPNSNPIRFGSFPPQCFLRVPKPRRLILPRFRKTTGGGGGLIRCELPDFHLSATATTVSGVSTVNLVDQVQIPKGEMWSVHKFGGTCVGNSQRIRNVAEVIINDNSERKLVVVSAMSKVTDMMYDLIRKAQSRDDSYLSALEAVLEKHRLTARDLLDGDDLASFLSHLHNDISNLKAMLRAIYIAGHASESFSDFVAGHGELWSAQMLSYVVRKTGLECKWMDTRDVLIVNPTSSNQVDPDFGESEKRLDKWFSLNPSKIIIATGFIASTPQNIPTTLKRDGSDFSAAIMGALLRARQVTIWTDVDGVYSADPRKVNEAVILQTLSYQEAWEMSYFGANVLHPRTIIPVMRYNIPIVIRNIFNLSAPGTIICQPPEDDYDLKLTTPVKGFATIDNLALINVEGTGMAGVPGTASDIFGCVKDVGANVIMISQASSEHSVCFAVPEKEVNAVSEALRSRFSEALQAGRLSQIEVIPNCSILAAVGQKMASTPGVSCTLFSALAKANINVRAISQGCSEYNVTVVIKREDSVKALRAVHSRFFLSRTTLAMGIVGPGLIGATLLDQLRDQAAVLKQEFNIDLRVLGITGSKKMLLSDIGIDLSRWRELLNEKGTEADLDKFTQQVHGNHFIPNSVVVDCTADSAIASRYYDWLRKGIHVITPNKKANSGPLDQYLKLRDLQRKSYTHYFYEATVGAGLPIISTLRGLLETGDKILRIEGICSGTLSYLFNNFVGDRSFSEVVTEAKNAGFTEPDPRDDLSGTDVARKVIILARESGLKLDLADLPIRSLVPEPLKGCTSVEEFMEKLPQYDGDLAKERLDAENSGEVLRYVGVVDAVNQKGTVELRRYKKEHPFAQLAGSDNIIAFTTTRYKDHPLIVRGPGAGAQVTAGGIFSDILRLASYLGAPS
-0.5
MATLKPSFTVSPPNSNPIRFGSFPPQCFLRVPKPRRLILPRFRKTTGGGGGLIRCELPDFHLSATATTVSGVSTVNLVDQVQIPKGEMWSVHKFGGTCVGNSQRIRNVAEVIINDNSERKLVVVSAMSKVTDMMYDLIRKAQSRDDSYLSALEAVLEKHRLTARDLLDGDDLASFLSHLHNDISNLKAMLRAIYIAGHASESFSDFVAGHGELWSAQMLSYVVRKTGLECKWMDTRDVLIVNPTSSNQVDPDFGESEKRLDKWFSLNPSKIIIATGFIASTPQNIPTTLKRDGSDFSAAIMGALLRARQVTIWTDVDGVYSADPRKVNEAVILQTLSYQEAWEMSYFGANVLHPRTIIPVMRYNIPIVIRNIFNLSAPGTIICQPPEDDYDLKLTTPVKGFATIDNLALINVEGTGMAGVPGTASDIFGCVKDVGANVIMISQASSEHSVCFAVPEKEVNAVSEALRSRFSEALQAGRLSQIEVIPNCSILAAVGQKMASTPGVSCTLFSALAKANINVRAISQGCSEYNVTVVIKREDSVKALRAVHSRFFLSRTTLAMGIVGPGLIGATLLDQLRDQAAVLKQEFNIDLRVLGITGSKKMLLSDIGIDLSRWRELLNEKGTEADLDKFTQQVHGNHFIPNSVVVDCTADSAIASRYYDWLRKGIHVITPNKKANSGPLDQYLKLRDLQRKSYTHYFYEATVGAGLPIISTLRGLLETGDKILRIEGICSGTLSYLFNNFVGDRSFSEVVTEAKNAGFTEPDPRDDLSGTDVARKVIILARESGLKLDLADLPIRSLVPEPLKGCTSVEEFMEKLPQYDGDLAKERLDAENSGEVLRYVGVVDAVNQKGTVELRRYKKEHPFAQLAGSDNIIAFTTTRYKDHPLIVRGPGAGAQVTAGGIFSDILRLASYLGAPS
Homoserine dehydrogenase domain
-1
Amino acid kinase family
ACT domain
ACT domain
-1.5
800
900
700
500
100
300
200
400
600
0
Protein length (amino acids)
Binding region from AT2G21490 replicates
Rep 2
Binding region from Glyma04g009900 replicates
Rep 2
X23
X17
Rep 3
For .1, 57 aa deletion and ~22 aa changes
All of the GmPM12 tags are valid for AT4G19710.1 (859 aa) but only a portion of LEA14 tag is valid for the shorter isoform.

## Slide 30
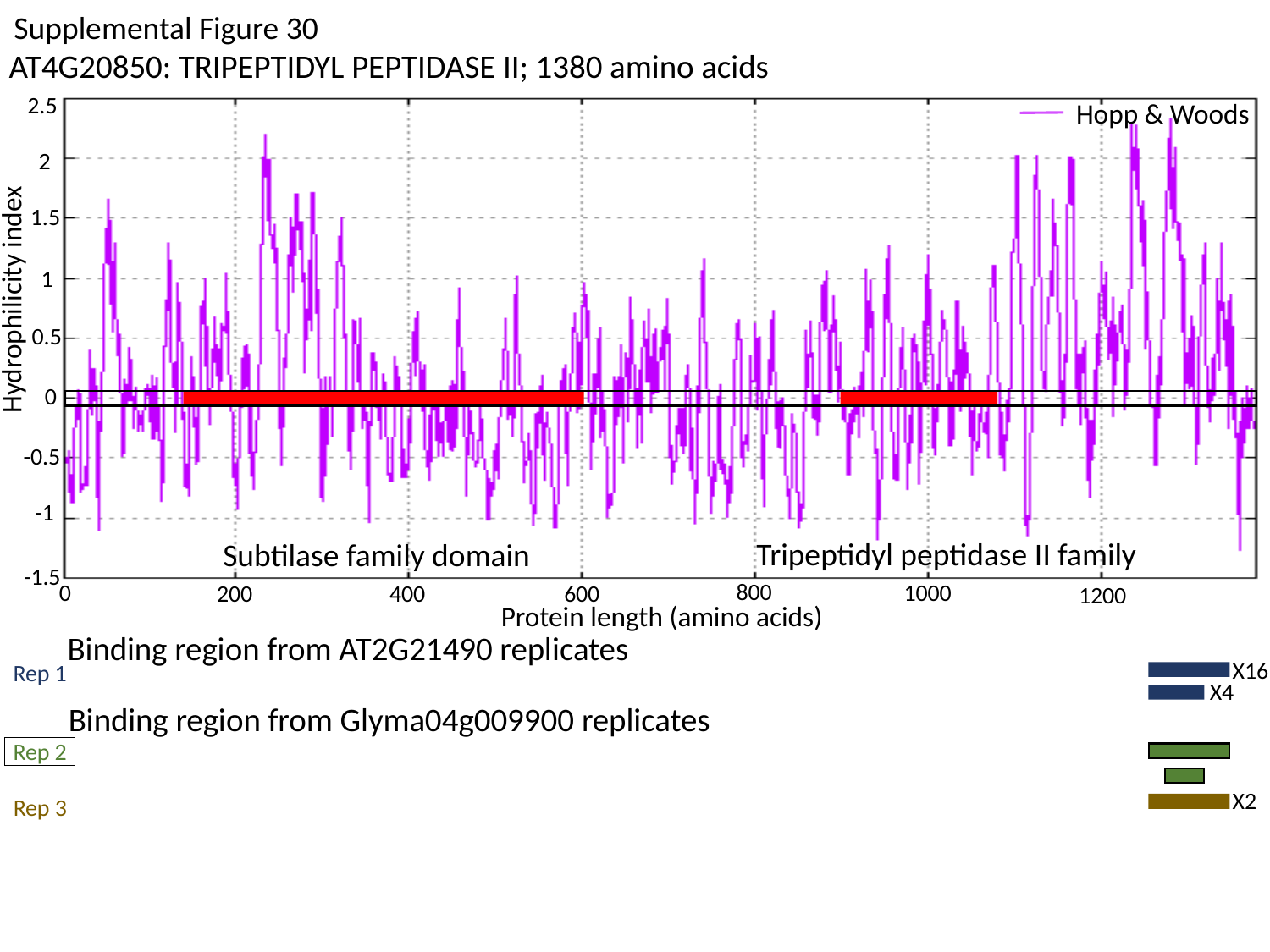

Supplemental Figure 30
AT4G20850: TRIPEPTIDYL PEPTIDASE II; 1380 amino acids
2.5
Hopp & Woods
2
1.5
1
Hydrophilicity index
0.5
0
MDLSLQLQIHGALINKGPSCTSYWASSSSLSLPRDFISSSTFLLHRRLRRRSCSRSRGIRLRRSGFSAMPCSSSDTLTASRVGCGGGGGGGAVGGGAENASVANFKLNESTFIASLMPKKEIRADCFIEAHPEYDGRGVVIAIFDSGFDPSAAGLHVTSDGKPKVLDVIDCTGSGDIDTSTVVKANEDGHIRGASGATLVVNSSWKNPTGEWRVGSKLVYQLFTDDLTSRVKKERRKSWDEKNQEEIAKAVNNLYDFDQKHSKVEDAKLKKTREDLQSKVDFLKKQADKYEDKGPVIDAVVWHDGEVWRVALDTQSLEEDPDSGKLADFSPLTNYRIERKYGVFSRLDACSFVANVYDEGKVLSIVTDSSPHGTHVAGIATAHHPEEHLLNGVAPGAQIISCKIGDSRLGSMETGTGLTRALIAALEHNCDLVNMSYGEPALLPDYGRFVDLVTEAVNKRRLIFVSSAGNSGPALTTVGAPGGTTSSIIGVGAYVSPAMAAGAHSVVEPPSEGLEYTWSSRGPTSDGDLGVCISAPGGAVAPVPTWTLQRRMLMNGTSMASPSACGAIALLLSAMKAEGIPVSPYSVRRALENTSTPVGDLPEDKLTTGQGLMQVDKAYEYLKQFQDYPCVFYQIKVNLSGKTIPTSRGIYLREGTACRQSTEWTIQVDPKFHEGASNLKELVPFEECLELHSTDEGVVRVPDYLLLTNNGRGFNVVVDPTNLGDGVHYFEVYGIDCKAPERGPLFRIPVTIIIPKTVANQPPVISFQQMSFISGHIERRYIEVPHGATWAEATMRTSGFDTTRRFYIDTLQVCPLRRPIKWESAPTFASPSAKSFVFPVVSGQTMELAIAQFWSSGLGSREPTIVDFEIEFHGVGVDKEELLLDGSEAPIKVEAEALLASEKLVPIAVLNKIRVPYQPIDAQLKTLSTGRDRLLSGKQILALTLTYKFKLEDSAEVKPYIPLLNNRIYDTKFESQFFMISDTNKRVYAMGDVYPESSKLPKGEYKLQLYLRHENVELLEKLKQLTVFIERNMGEIRLNLHSEPDGPFTGNGAFKSSVLMPGVKEAFYLGPPTKDKLPKNTPQGSMLVGEISYGKLSFDEKEGKNPKDNPVSYPISYVVPPNKPEEDKKAASAPTCSKSVSERLEQEVRDTKIKFLGNLKQETEEERSEWRKLCTCLKSEYPDYTPLLAKILEGLLSRSDAGDKISHHEEIIEAANEVVRSVDVDELARFLLDKTEPEDDEAEKLKKKMEVTRDQLADALYQKGLAMARIENLKGEKEGEGEEESSQKDKFEENFKELTKWVDVKSSKYGTLTVLREKRLSRLGTALKVLDDLIQNENETANKKLYELKLDLLEEIGWSHLVTYEKQWMQVRFPKSLPLF
-0.5
-1
Tripeptidyl peptidase II family
Subtilase family domain
-1.5
800
0
1000
200
400
600
1200
Protein length (amino acids)
Binding region from AT2G21490 replicates
X16
Rep 1
X4
Binding region from Glyma04g009900 replicates
Rep 2
X2
Rep 3

## Slide 31
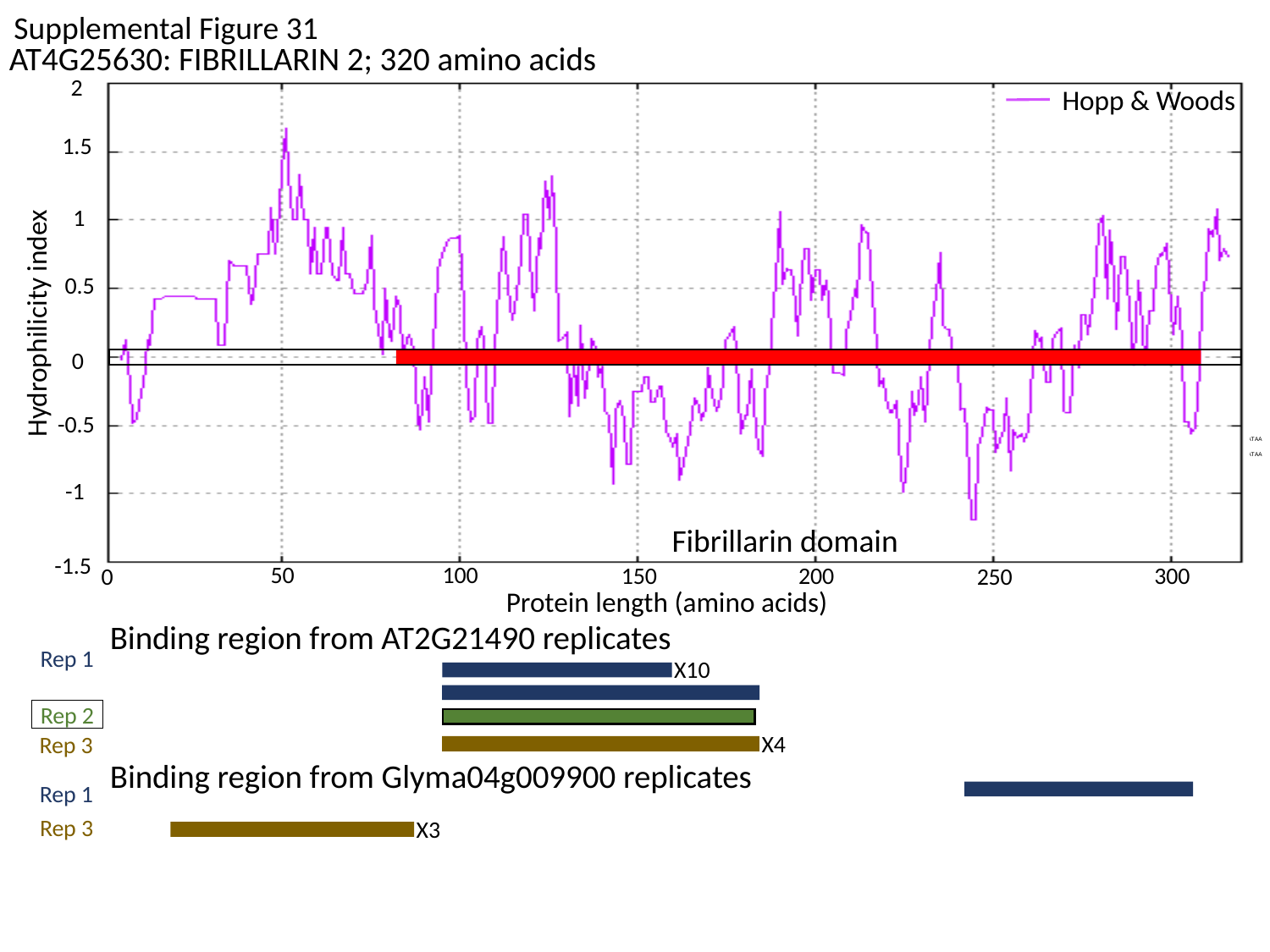

Supplemental Figure 31
AT4G25630: FIBRILLARIN 2; 320 amino acids
2
Hopp & Woods
1.5
1
0.5
Hydrophilicity index
0
-0.5
MRPPLTGSGGGFSGGRGRGGYSGGRGDGGFSGGRGGGGRGGGRGFSDRGGRGRGRGPPRGGARGGRGPAGRGGMKGGSKVIVEPHRHAGVFIAKGKEDALVTKNLVPGEAVYNEKRISVQNEDGTKTEYRVWNPFRSKLAAAILGGVDNIWIKPGAKVLYLGAASGTTVSHVSDLVGPEGCVYAVEFSHRSGRDLVNMAKKRTNVIPIIEDARHPAKYRMLVGMVDVIFSDVAQPDQARILALNASYFLKSGGHFVISIKANCIDSTVPAEAVFQTEVKKLQQEQFKPAEQVTLEPFERDHACVVGGYRMPKKPKAATAA
MRPPLTGSGGGFSGGRGRGGYSGGRGDGGFSGGRGGGGRGGGRGFSDRGGRGRGRGPPRGGARGGRGPAGRGGMKGGSKVIVEPHRHAGVFIAKGKEDALVTKNLVPGEAVYNEKRISVQNEDGTKTEYRVWNPFRSKLAAAILGGVDNIWIKPGAKVLYLGAASGTTVSHVSDLVGPEGCVYAVEFSHRSGRDLVNMAKKRTNVIPIIEDARHPAKYRMLVGMVDVIFSDVAQPDQARILALNASYFLKSGGHFVISIKANCIDSTVPAEAVFQTEVKKLQQEQFKPAEQVTLEPFERDHACVVGGYRMPKKPKAATAA
-1
Fibrillarin domain
-1.5
50
100
200
150
300
0
250
Protein length (amino acids)
Binding region from AT2G21490 replicates
Rep 1
X10
Rep 2
X4
Rep 3
Binding region from Glyma04g009900 replicates
Rep 1
Rep 3
X3

## Slide 32
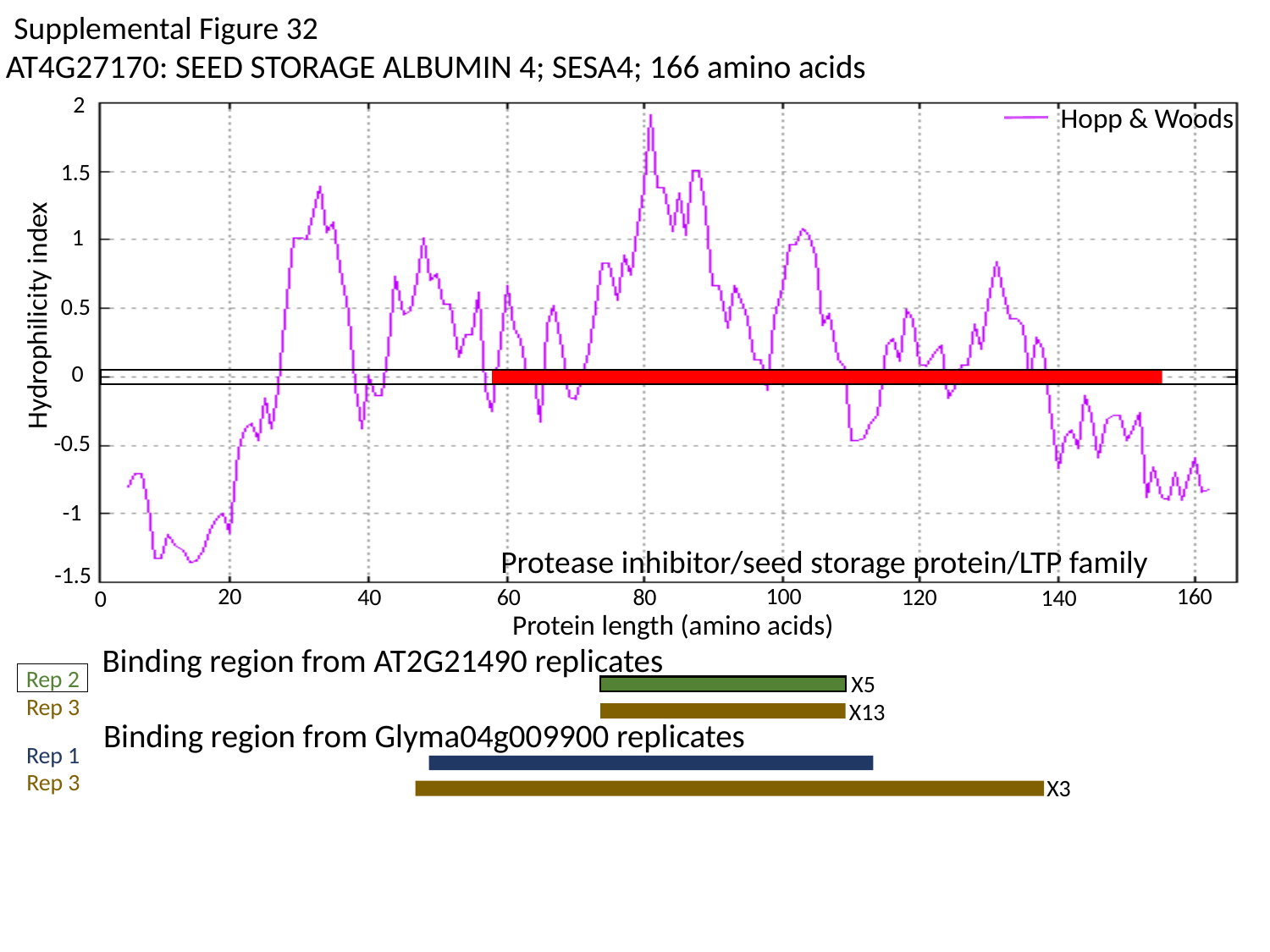

Supplemental Figure 32
AT4G27170: SEED STORAGE ALBUMIN 4; SESA4; 166 amino acids
2
Hopp & Woods
1.5
1
0.5
Hydrophilicity index
0
MANKLFLVCAALALCFILTNASVYRTVVEFDEDDASNPIGPIQKCQKEFQQDQHLRACQRWMRKQMWQGRGGGPSLDDEFDMEDDIENPQRRQLLQKCCSELRQEEPVCVCPTLRQAAKAVRFQGQQHQPEQVRKIYQAAKYLPNICKIQQVGVCPFQIPSIPSYY
-0.5
MANKLFLVCAALALCFILTNASVYRTVVEFDEDDASNPIGPIQKCQKEFQQDQHLRACQRWMRKQMWQGRGGGPSLDDEFDMEDDIENPQRRQLLQKCCSELRQEEPVCVCPTLRQAAKAVRFQGQQHQPEQVRKIYQAAKYLPNICKIQQVGVCPFQIPSIPSYY
-1
Protease inhibitor/seed storage protein/LTP family
-1.5
160
100
20
60
120
40
80
140
0
Protein length (amino acids)
Binding region from AT2G21490 replicates
X5
Rep 2
Rep 3
X13
Binding region from Glyma04g009900 replicates
Rep 1
Rep 3
X3

## Slide 33
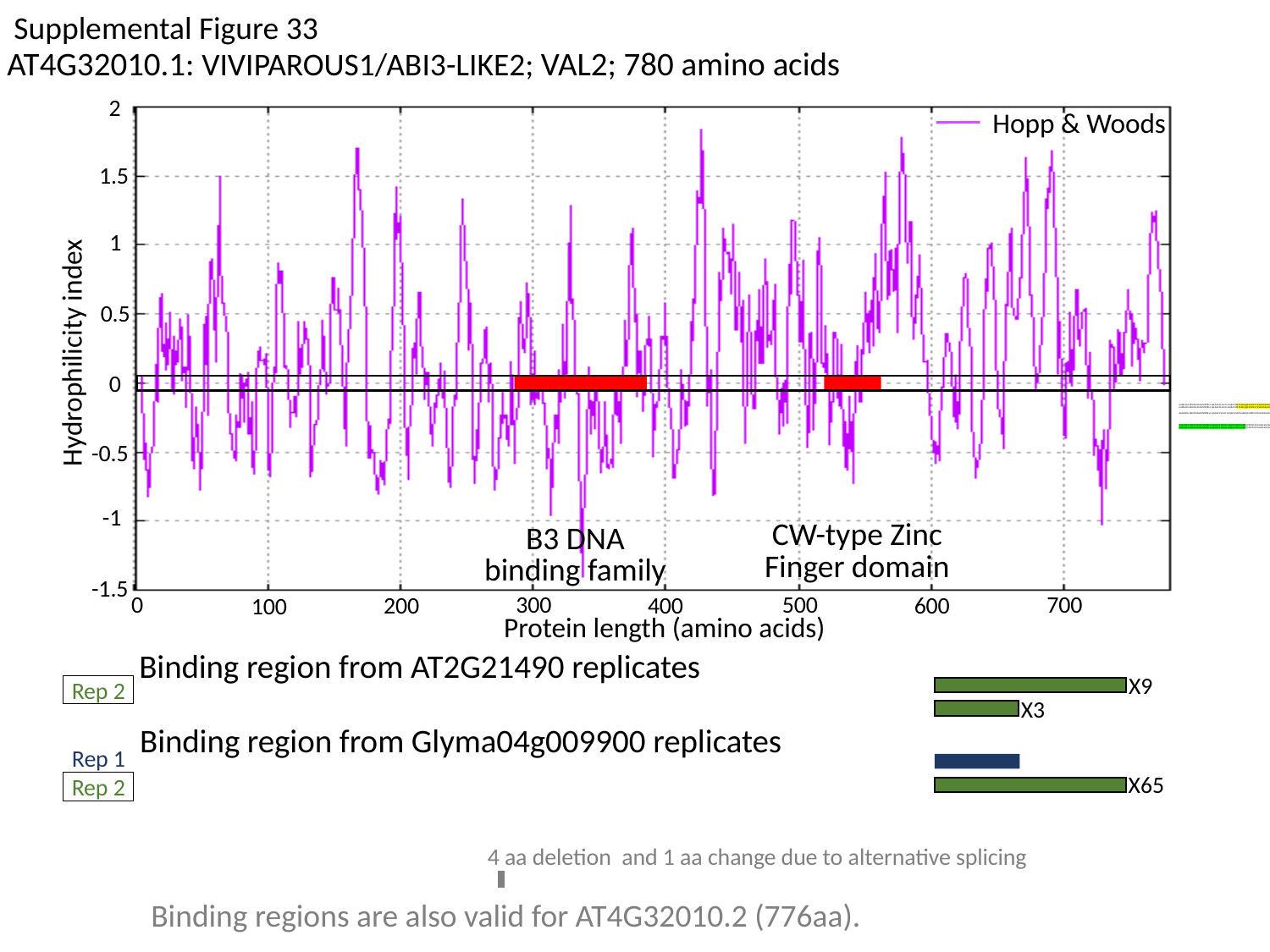

Supplemental Figure 33
AT4G32010.1: VIVIPAROUS1/ABI3-LIKE2; VAL2; 780 amino acids
2
Hopp & Woods
1.5
1
0.5
Hydrophilicity index
0
MESIKVCMNALCGAASTSGEWKKGWPMRSGDLASLCDKCGCAYEQSIFCEVFHAKESGWRECNSCDKRLHCGCIASRFMMELLENGGVTCISCAKKSGLISMNVSHESNGKDFPSFASAEHVGSVLERTNLKHLLHFQRIDPTHSSLQMKQEESLLPSSLDALRHKTERKELSAQPNLSISLGPTLMTSPFHDAAVDDRSKTNSIFQLAPRSRQLLPKPANSAPIAAGMEPSGSLVSQIHVARPPPEGRGKTQLLPRYWPRITDQELLQLSGQYPHLSNSKIIPLFEKVLSASDAGRIGRLVLPKACAEAYFPPISLPEGLPLKIQDIKGKEWVFQFRFWPNNNSRMYVLEGVTPCIQSMQLQAGDTVTFSRTEPEGKLVMGYRKATNSTATQMFKGSSEPNLNMFSNSLNPGCGDINWSKLEKSEDMAKDNLFLQSSLTSARKRVRNIGTKSKRLLIDSVDVLELKITWEEAQELLRPPQSTKPSIFTLENQDFEEYDEPPVFGKRTLFVSRQTGEQEQWVQCDACGKWRQLPVDILLPPKWSCSDNLLDPGRSSCSAPDELSPREQDTLVRQSKEFKRRRLASSNEKLNQSQDASALNSLGNAGITTTGEQGEITVAATTKHPRHRAGCSCIVCSQPPSGKGKHKPSCTCTVCEAVKRRFRTLMLRKRNKGEAGQASQQAQSQSECRDETEVESIPAVELAAGENIDLNSDPGASRVSMMRLLQAAAFPLEAYLKQKAISNTAGEQQSSDMVSTEHGSSSAAQETEKDTTNGAHDPVN
MESIKVCMNALCGAASTSGEWKKGWPMRSGDLASLCDKCGCAYEQSIFCEVFHAKESGWRECNSCDKRLHCGCIASRFMMELLENGGVTCISCAKKSGLISMNVSHESNGKDFPSFASAEHVGSVLERTNLKHLLHFQRIDPTHSSLQMKQEESLLPSSLDALRHKTERKELSAQPNLSISLGPTLMTSPFHDAAVDDRSKTNSIFQLAPRSRQLLPKPANSAPIAAGMEPSGSLVSQIHVARPPPEGRGKTQLLPRYWPRITDQELLQLSGQYPHLSNSKIIPLFEKVLSASDAGRIGRLVLPKACAEAYFPPISLPEGLPLKIQDIKGKEWVFQFRFWPNNNSRMYVLEGVTPCIQSMQLQAGDTVTFSRTEPEGKLVMGYRKATNSTATQMFKGSSEPNLNMFSNSLNPGCGDINWSKLEKSEDMAKDNLFLQSSLTSARKRVRNIGTKSKRLLIDSVDVLELKITWEEAQELLRPPQSTKPSIFTLENQDFEEYDEPPVFGKRTLFVSRQTGEQEQWVQCDACGKWRQLPVDILLPPKWSCSDNLLDPGRSSCSAPDELSPREQDTLVRQSKEFKRRRLASSNEKLNQSQDASALNSLGNAGITTTGEQGEITVAATTKHPRHRAGCSCIVCSQPPSGKGKHKPSCTCTVCEAVKRRFRTLMLRKRNKGEAGQASQQAQSQSECRDETEVESIPAVELAAGENIDLNSDPGASRVSMMRLLQAAAFPLEAYLKQKAISNTAGEQQSSDMVSTEHGSSSAAQETEKDTTNGAHDPVN
MESIKVCMNALCGAASTSGEWKKGWPMRSGDLASLCDKCGCAYEQSIFCEVFHAKESGWRECNSCDKRLHCGCIASRFMMELLENGGVTCISCAKKSGLISMNVSHESNGKDFPSFASAEHVGSVLERTNLKHLLHFQRIDPTHSSLQMKQEESLLPSSLDALRHKTERKELSAQPNLSISLGPTLMTSPFHDAAVDDRSKTNSIFQLAPRSRQLLPKPANSAPIAAGMEPSGSLVSQIHVARPPPEGRGKTQLLPRYWPRITDQELLQLSGQYPHLSNSKIIPLFEKVLSASDAGRIGRLVLPKACAEAYFPPISLPEGLPLKIQDIKGKEWVFQFRFWPNNNSRMYVLEGVTPCIQSMQLQAGDTVTFSRTEPEGKLVMGYRKATNSTATQMFKGSSEPNLNMFSNSLNPGCGDINWSKLEKSEDMAKDNLFLQSSLTSARKRVRNIGTKSKRLLIDSVDVLELKITWEEAQELLRPPQSTKPSIFTLENQDFEEYDEPPVFGKRTLFVSRQTGEQEQWVQCDACGKWRQLPVDILLPPKWSCSDNLLDPGRSSCSAPDELSPREQDTLVRQSKEFKRRRLASSNEKLNQSQDASALNSLGNAGITTTGEQGEITVAATTKHPRHRAGCSCIVCSQPPSGKGKHKPSCTCTVCEAVKRRFRTLMLRKRNKGEAGQASQQAQSQSECRDETEVESIPAVELAAGENIDLNSDPGASRVSMMRLLQAAAFPLEAYLKQKAISNTAGEQQSSDMVSTEHGSSSAAQETEKDTTNGAHDPVN
MESIKVCMNALCGAASTSGEWKKGWPMRSGDLASLCDKCGCAYEQSIFCEVFHAKESGWRECNSCDKRLHCGCIASRFMMELLENGGVTCISCAKKSGLISMNVSHESNGKDFPSFASAEHVGSVLERTNLKHLLHFQRIDPTHSSLQMKQEESLLPSSLDALRHKTERKELSAQPNLSISLGPTLMTSPFHDAAVDDRSKTNSIFQLAPRSRQLLPKPANSAPIAAGMEPSGSLVSQIHVARPPPEGRGKTQLLPRYWPRITDQELLQLSGQYPHLSNSKIIPLFEKVLSASDAGRIGRLVLPKACAEAYFPPISLPEGLPLKIQDIKGKEWVFQFRFWPNNNSRMYVLEGVTPCIQSMQLQAGDTVTFSRTEPEGKLVMGYRKATNSTATQMFKGSSEPNLNMFSNSLNPGCGDINWSKLEKSEDMAKDNLFLQSSLTSARKRVRNIGTKSKRLLIDSVDVLELKITWEEAQELLRPPQSTKPSIFTLENQDFEEYDEPPVFGKRTLFVSRQTGEQEQWVQCDACGKWRQLPVDILLPPKWSCSDNLLDPGRSSCSAPDELSPREQDTLVRQSKEFKRRRLASSNEKLNQSQDASALNSLGNAGITTTGEQGEITVAATTKHPRHRAGCSCIVCSQPPSGKGKHKPSCTCTVCEAVKRRFRTLMLRKRNKGEAGQASQQAQSQSECRDETEVESIPAVELAAGENIDLNSDPGASRVSMMRLLQAAAFPLEAYLKQKAISNTAGEQQSSDMVSTEHGSSSAAQETEKDTTNGAHDPVN
MESIKVCMNALCGAASTSGEWKKGWPMRSGDLASLCDKCGCAYEQSIFCEVFHAKESGWRECNSCDKRLHCGCIASRFMMELLENGGVTCISCAKKSGLISMNVSHESNGKDFPSFASAEHVGSVLERTNLKHLLHFQRIDPTHSSLQMKQEESLLPSSLDALRHKTERKELSAQPNLSISLGPTLMTSPFHDAAVDDRSKTNSIFQLAPRSRQLLPKPANSAPIAAGMEPSGSLVSQIHVARPPPEGRGKTQLLPRYWPRITDQELLQLSGQYPHLSNSKIIPLFEKVLSASDAGRIGRLVLPKACAEAYFPPISLPEGLPLKIQDIKGKEWVFQFRFWPNNNSRMYVLEGVTPCIQSMQLQAGDTVTFSRTEPEGKLVMGYRKATNSTATQMFKGSSEPNLNMFSNSLNPGCGDINWSKLEKSEDMAKDNLFLQSSLTSARKRVRNIGTKSKRLLIDSVDVLELKITWEEAQELLRPPQSTKPSIFTLENQDFEEYDEPPVFGKRTLFVSRQTGEQEQWVQCDACGKWRQLPVDILLPPKWSCSDNLLDPGRSSCSAPDELSPREQDTLVRQSKEFKRRRLASSNEKLNQSQDASALNSLGNAGITTTGEQGEITVAATTKHPRHRAGCSCIVCSQPPSGKGKHKPSCTCTVCEAVKRRFRTLMLRKRNKGEAGQASQQAQSQSECRDETEVESIPAVELAAGENIDLNSDPGASRVSMMRLLQAAAFPLEAYLKQKAISNTAGEQQSSDMVSTEHGSSSAAQETEKDTTNGAHDPVN
-0.5
-1
CW-type Zinc Finger domain
B3 DNA binding family
-1.5
0
700
500
300
200
400
600
100
Protein length (amino acids)
Binding region from AT2G21490 replicates
X9
Rep 2
X3
Binding region from Glyma04g009900 replicates
Rep 1
X65
Rep 2
4 aa deletion and 1 aa change due to alternative splicing
Binding regions are also valid for AT4G32010.2 (776aa).

## Slide 34
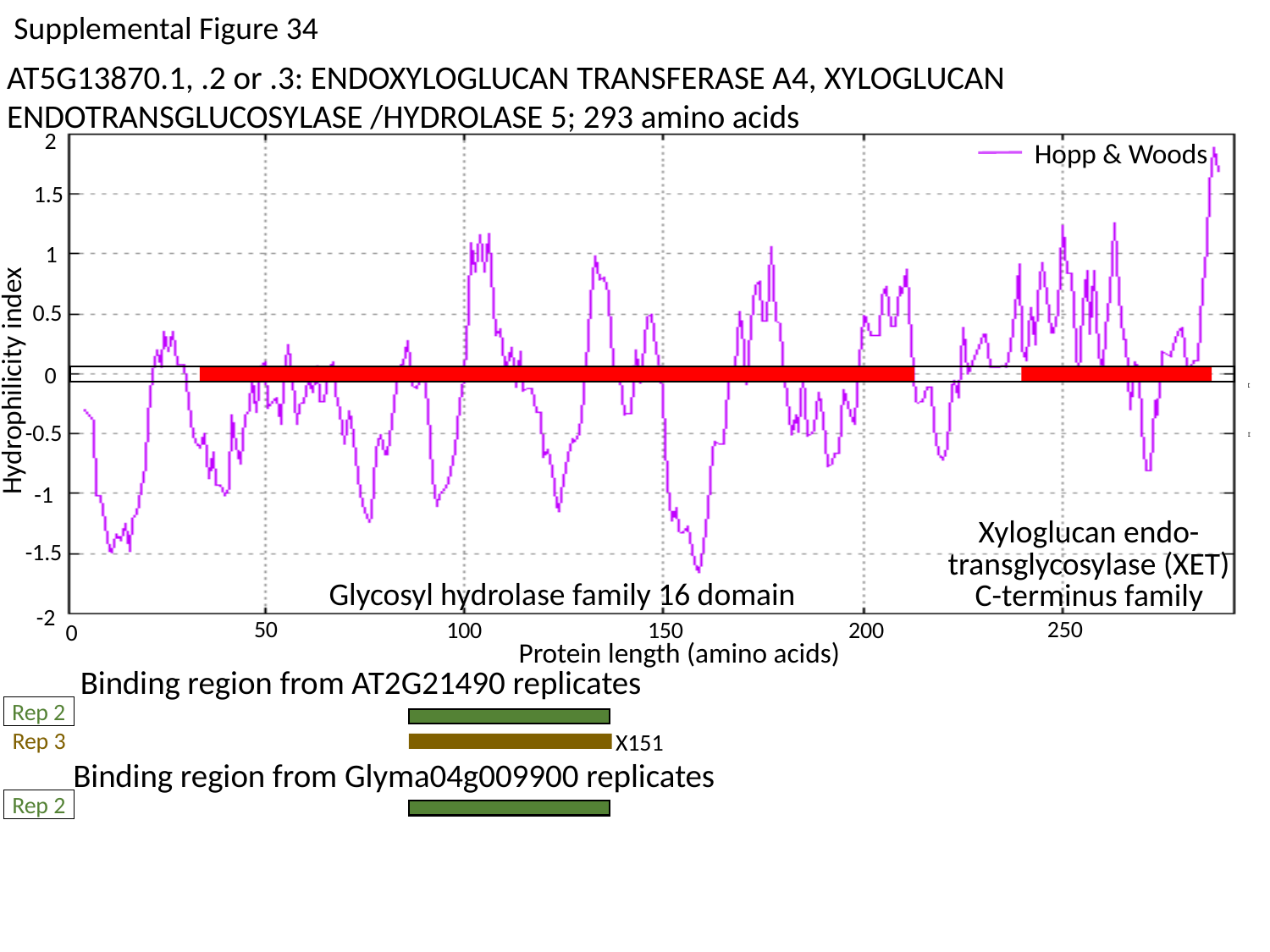

Supplemental Figure 34
AT5G13870.1, .2 or .3: ENDOXYLOGLUCAN TRANSFERASE A4, XYLOGLUCAN ENDOTRANSGLUCOSYLASE /HYDROLASE 5; 293 amino acids
2
Hopp & Woods
1.5
1
0.5
0
Hydrophilicity index
MGRLSSTLCLTFLILATVAFGVPPKKSINVPFGRNYFPTWAFDHIKYLNGGSEVHLVLDKYTGTGFQSKGSYLFGHFSMHIKMVAGDSAGTVTAFYLSSQNSEHDEIDFEFLGNRTGQPYILQTNVFTGGAGNREQRINLWFDPSKDYHSYSVLWNMYQIVFFVDDVPIRVFKNSKDVGVKFPFNQPMKIYSSLWNADDWATRGGLEKTNWEKAPFVASYRGFHVDGCEASVNAKFCETQGKRWWDQKEFQDLDANQYKRLKWVRKRYTIYNYCTDRVRFPVPPPECRRDRDI
-0.5
MGRLSSTLCLTFLILATVAFGVPPKKSINVPFGRNYFPTWAFDHIKYLNGGSEVHLVLDKYTGTGFQSKGSYLFGHFSMHIKMVAGDSAGTVTAFYLSSQNSEHDEIDFEFLGNRTGQPYILQTNVFTGGAGNREQRINLWFDPSKDYHSYSVLWNMYQIVFFVDDVPIRVFKNSKDVGVKFPFNQPMKIYSSLWNADDWATRGGLEKTNWEKAPFVASYRGFHVDGCEASVNAKFCETQGKRWWDQKEFQDLDANQYKRLKWVRKRYTIYNYCTDRVRFPVPPPECRRDRDI
-1
Xyloglucan endo-transglycosylase (XET) C-terminus family
-1.5
Glycosyl hydrolase family 16 domain
-2
250
50
150
200
100
0
Protein length (amino acids)
Binding region from AT2G21490 replicates
Rep 2
Rep 3
X151
Binding region from Glyma04g009900 replicates
Rep 2

## Slide 35
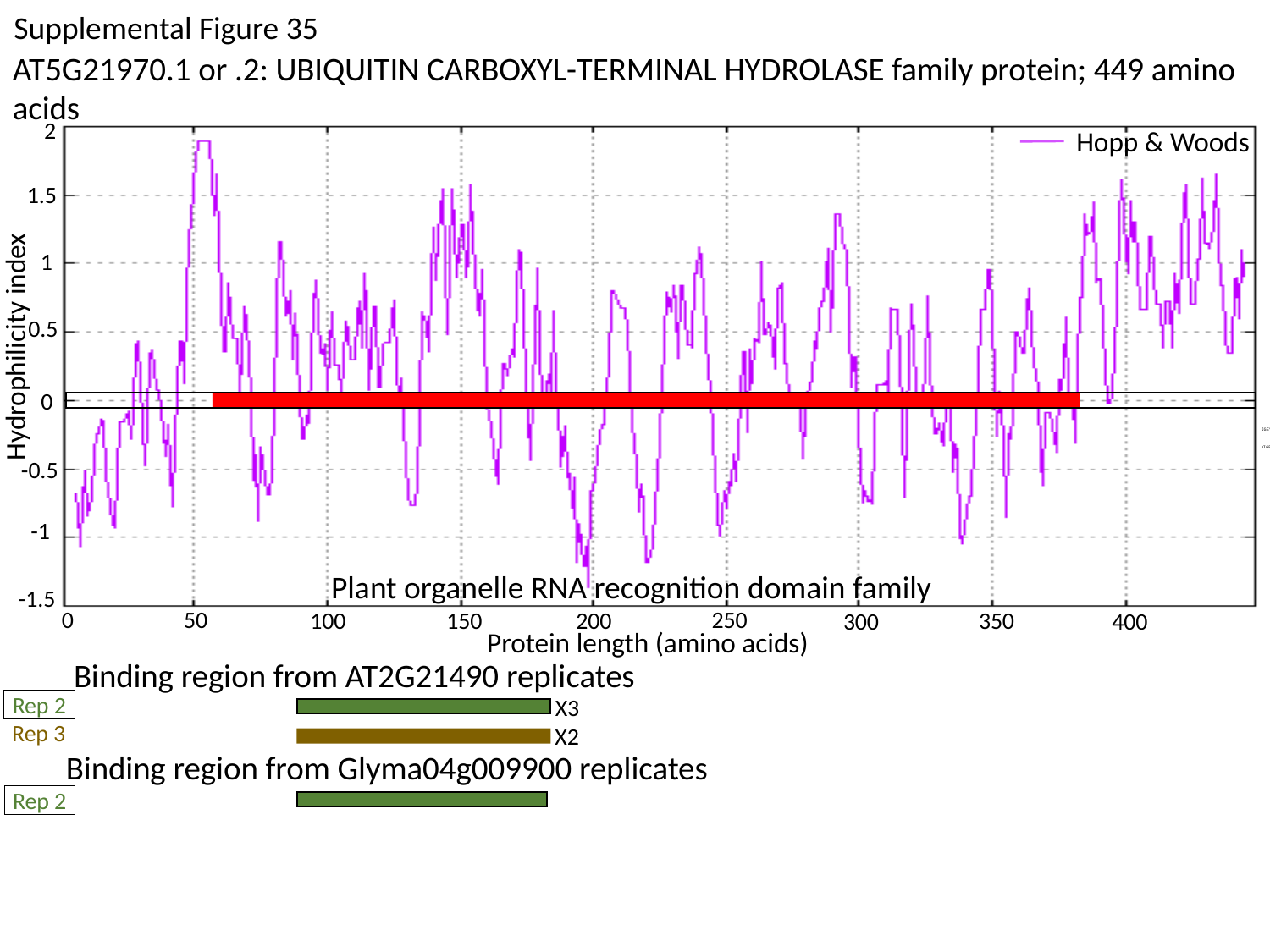

Supplemental Figure 35
AT5G21970.1 or .2: UBIQUITIN CARBOXYL-TERMINAL HYDROLASE family protein; 449 amino acids
2
Hopp & Woods
1.5
1
0.5
Hydrophilicity index
0
MGGLRCWLQFYRNGNAHNVFAIMPERNVLYGESSFRRVWVRFMTSSKRVQDRSREKRVQELEIATEKWKIASKVIFLMEVLKGERDMIMTVRSFEQYRRQINLPKPHKISDFIRKSPKLFELYKDQRGVLWCGLTEGGEDLLDEHDKLLEENGDKAAEHVTRCLMMSVDKKLPLDKIVHFRRDFGLPLDFRINWVYNFPQHFKVVKLGDGEEYLELVSWNPAWAITELEKKTLGITEDCEHKPGMLSLAFPMKFPPSYKKMYRYRGKIEHFQKRSYLSPYADARGLEAGSKEFDKRAIAVMHELLSFTLEKRLVTDHLTHFRREFVMPQKLMRIFLKHCGIFYVSERGKRFSVFLTEGYEGPELIEKCPLILWKEKLLKFTGYRGRKRDIQTYSDTLDMEERELLESGSGDEDLSVGFEKDGDYDDVVTDDDEMDIGEVNDAYEENSKV
MGGLRCWLQFYRNGNAHNVFAIMPERNVLYGESSFRRVWVRFMTSSKRVQDRSREKRVQELEIATEKWKIASKVIFLMEVLKGERDMIMTVRSFEQYRRQINLPKPHKISDFIRKSPKLFELYKDQRGVLWCGLTEGGEDLLDEHDKLLEENGDKAAEHVTRCLMMSVDKKLPLDKIVHFRRDFGLPLDFRINWVYNFPQHFKVVKLGDGEEYLELVSWNPAWAITELEKKTLGITEDCEHKPGMLSLAFPMKFPPSYKKMYRYRGKIEHFQKRSYLSPYADARGLEAGSKEFDKRAIAVMHELLSFTLEKRLVTDHLTHFRREFVMPQKLMRIFLKHCGIFYVSERGKRFSVFLTEGYEGPELIEKCPLILWKEKLLKFTGYRGRKRDIQTYSDTLDMEERELLESGSGDEDLSVGFEKDGDYDDVVTDDDEMDIGEVNDAYEENSKV
-0.5
-1
Plant organelle RNA recognition domain family
-1.5
0
250
50
150
100
200
350
400
300
Protein length (amino acids)
Binding region from AT2G21490 replicates
X3
Rep 2
Rep 3
X2
Binding region from Glyma04g009900 replicates
Rep 2

## Slide 36
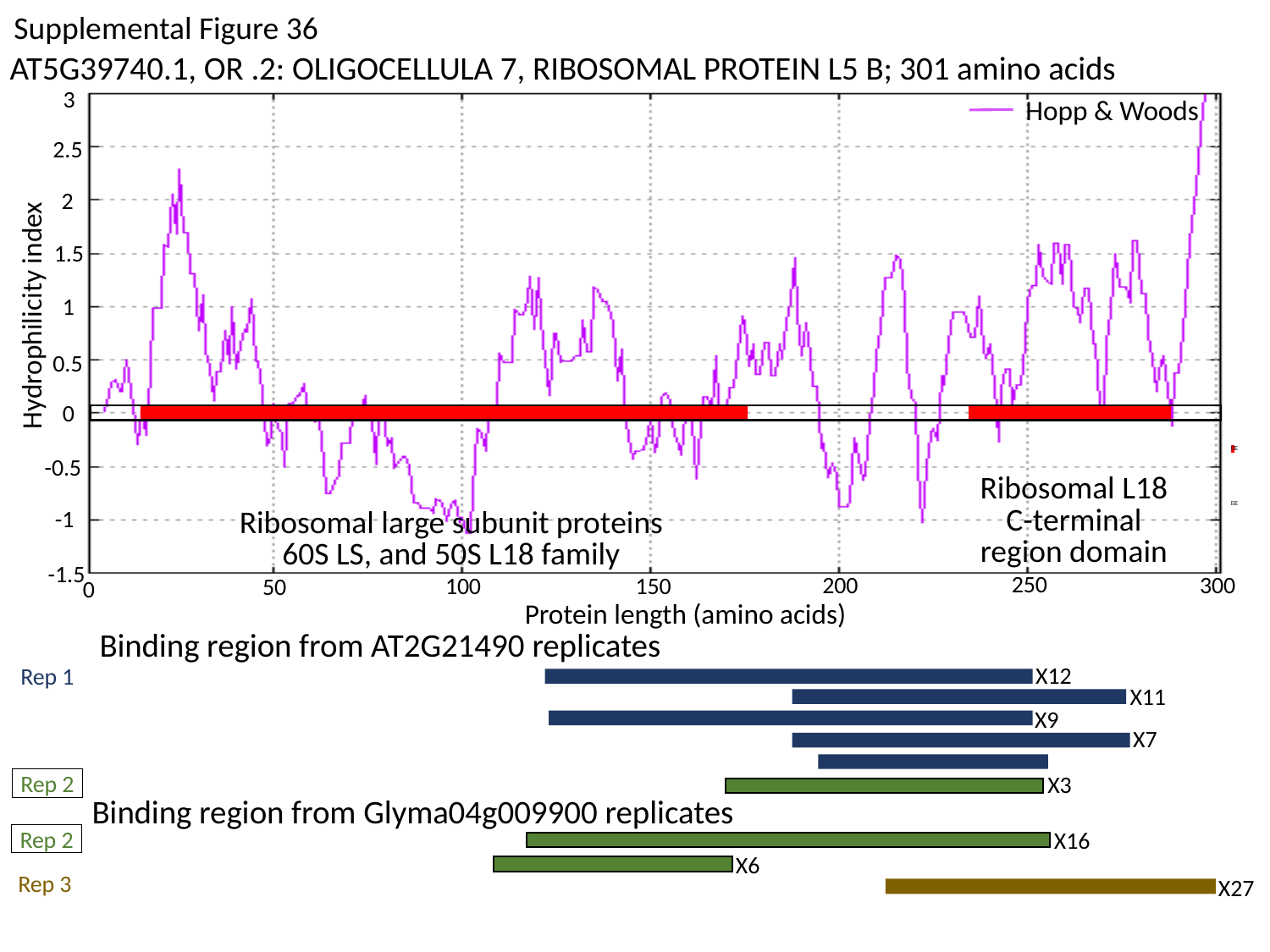

Supplemental Figure 36
AT5G39740.1, OR .2: OLIGOCELLULA 7, RIBOSOMAL PROTEIN L5 B; 301 amino acids
3
Hopp & Woods
2.5
2
1.5
1
Hydrophilicity index
0.5
0
MVFVKSSKSNAYFKRYQVKFRRRRDGKTDYRARIRLINQDKNKYNTPKYRFVVRFTNKDIVAQIVSASIAGDIVKASAYAHELPQYGLTVGLTNYAAAYCTGLLLARRVLKMLEMDDEYEGNVEATGEDFSVEPTDSRRPFRALLDVGLIRTTTGNRVFGALKGALDGGLDIPHSDKRFAGFHKENKQLDAEIHRNYIYGGHVSNYMKLLGEDEPEKLQTHFSAYIKKGVEAESIEEMYKKVHAAIRAEPNHKKTEKSAPKEHKRYNLKKLTYEERKNKLIERVKALNGAGGDDDDEDDEE
-0.5
Ribosomal L18 C-terminal region domain
MVFVKSSKSNAYFKRYQVKFRRRRDGKTDYRARIRLINQDKNKYNTPKYRFVVRFTNKDIVAQIVSASIAGDIVKASAYAHELPQYGLTVGLTNYAAAYCTGLLLARRVLKMLEMDDEYEGNVEATGEDFSVEPTDSRRPFRALLDVGLIRTTTGNRVFGALKGALDGGLDIPHSDKRFAGFHKENKQLDAEIHRNYIYGGHVSNYMKLLGEDEPEKLQTHFSAYIKKGVEAESIEEMYKKVHAAIRAEPNHKKTEKSAPKEHKRYNLKKLTYEERKNKLIERVKALNGAGGDDDDEDDEE
-1
Ribosomal large subunit proteins 60S LS, and 50S L18 family
-1.5
250
300
200
150
100
50
0
Protein length (amino acids)
Binding region from AT2G21490 replicates
X12
Rep 1
X11
X9
X7
X3
Rep 2
Binding region from Glyma04g009900 replicates
X16
Rep 2
X6
Rep 3
X27

## Slide 37
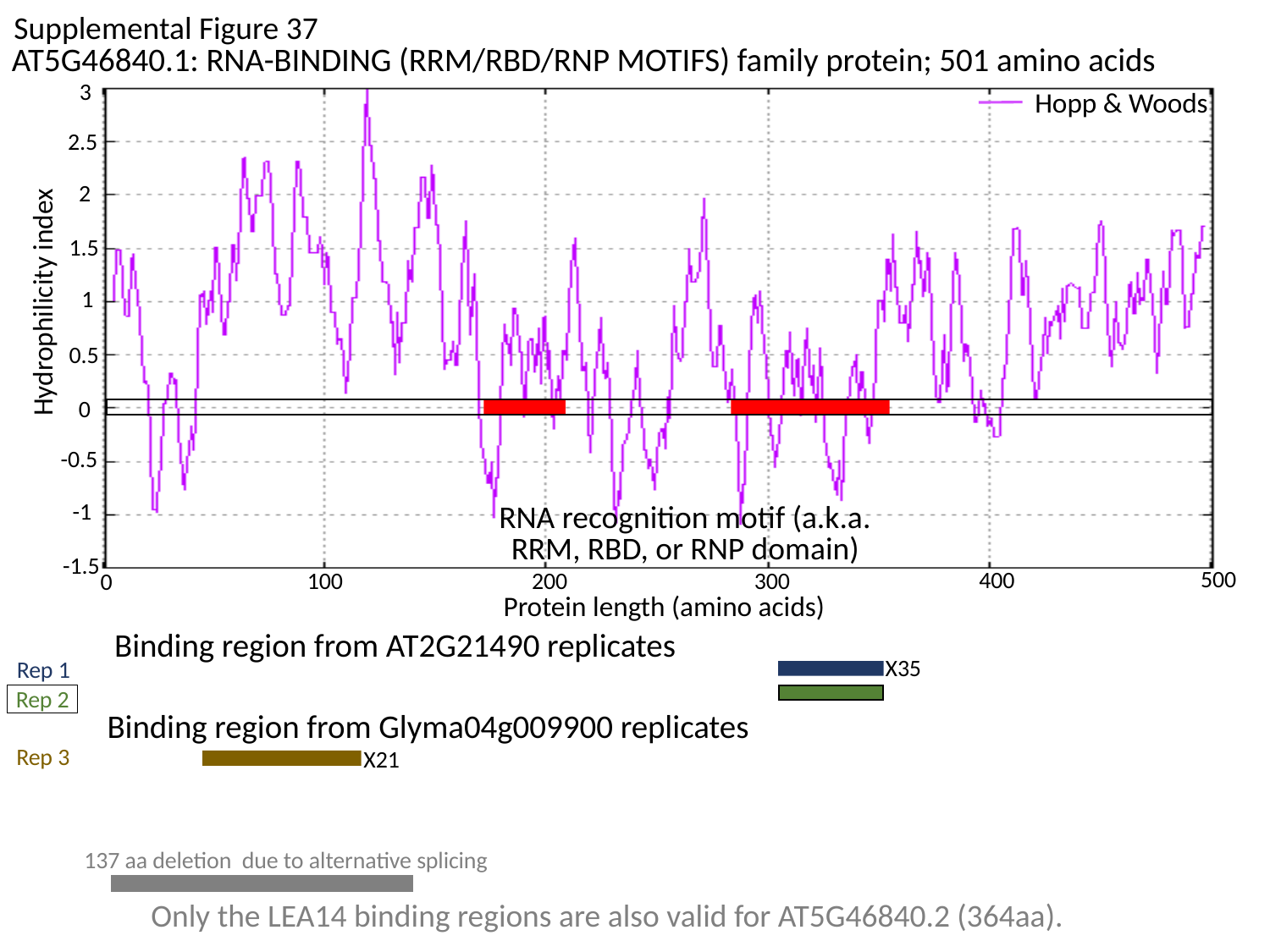

Supplemental Figure 37
AT5G46840.1: RNA-BINDING (RRM/RBD/RNP MOTIFS) family protein; 501 amino acids
3
Hopp & Woods
2.5
2
1.5
1
Hydrophilicity index
0.5
0
-0.5
MGKKKSKVTEPESQNDESQTIFSTLFSGEVESSGFASLFSADNPFRRKQPQEIKESSIPDEKKGDKRNAENEEEEEETDLPVKTKKSKKEKKLTDSGDEKETISEAVEESGLVSKRKKRKRDEIENEYETKKYGSVEMKEKKVGEKRKKADEVADTMVSKEGFDDESKLLRTVFVGNLPLKVKKKVILKEFSKFGEVESVRIRSVPIVDSKRTRKGAIMLKQINEKASSVHAYVVFETEQSAAASLAHNMSLIDGNHVRVDRACPPRKKQKGHDDTHLYDPKRTVFMGNLPFDVKDEEVYQLFTGKSNLENSIEAVRVIRDPHLNIGKGIAYVLFKTREAANLVLKKGYLKLRERELRISRVKPDTPSKRKSNPSEAYSPAQKRLQKDKVVTPTPTGKANLSYQGVRASKSGDDKKTPYQKSSAQTKMRPRGSSSNDNKKSGNNSASKERSQKRPAVAARKAKANAKGSKESGGKRFAGTKRKQENRTPESFSKKKKTKRF
MGKKKSKVTEPESQNDESQTIFSTLFSGEVESSGFASLFSADNPFRRKQPQEIKESSIPDEKKGDKRNAENEEEEEETDLPVKTKKSKKEKKLTDSGDEKETISEAVEESGLVSKRKKRKRDEIENEYETKKYGSVEMKEKKVGEKRKKADEVADTMVSKEGFDDESKLLRTVFVGNLPLKVKKKVILKEFSKFGEVESVRIRSVPIVDSKRTRKGAIMLKQINEKASSVHAYVVFETEQSAAASLAHNMSLIDGNHVRVDRACPPRKKQKGHDDTHLYDPKRTVFMGNLPFDVKDEEVYQLFTGKSNLENSIEAVRVIRDPHLNIGKGIAYVLFKTREAANLVLKKGYLKLRERELRISRVKPDTPSKRKSNPSEAYSPAQKRLQKDKVVTPTPTGKANLSYQGVRASKSGDDKKTPYQKSSAQTKMRPRGSSSNDNKKSGNNSASKERSQKRPAVAARKAKANAKGSKESGGKRFAGTKRKQENRTPESFSKKKKTKRF
-1
RNA recognition motif (a.k.a. RRM, RBD, or RNP domain)
-1.5
500
400
100
300
200
0
Protein length (amino acids)
Binding region from AT2G21490 replicates
X35
Rep 1
Rep 2
Binding region from Glyma04g009900 replicates
Rep 3
X21
137 aa deletion due to alternative splicing
Only the LEA14 binding regions are also valid for AT5G46840.2 (364aa).

## Slide 38
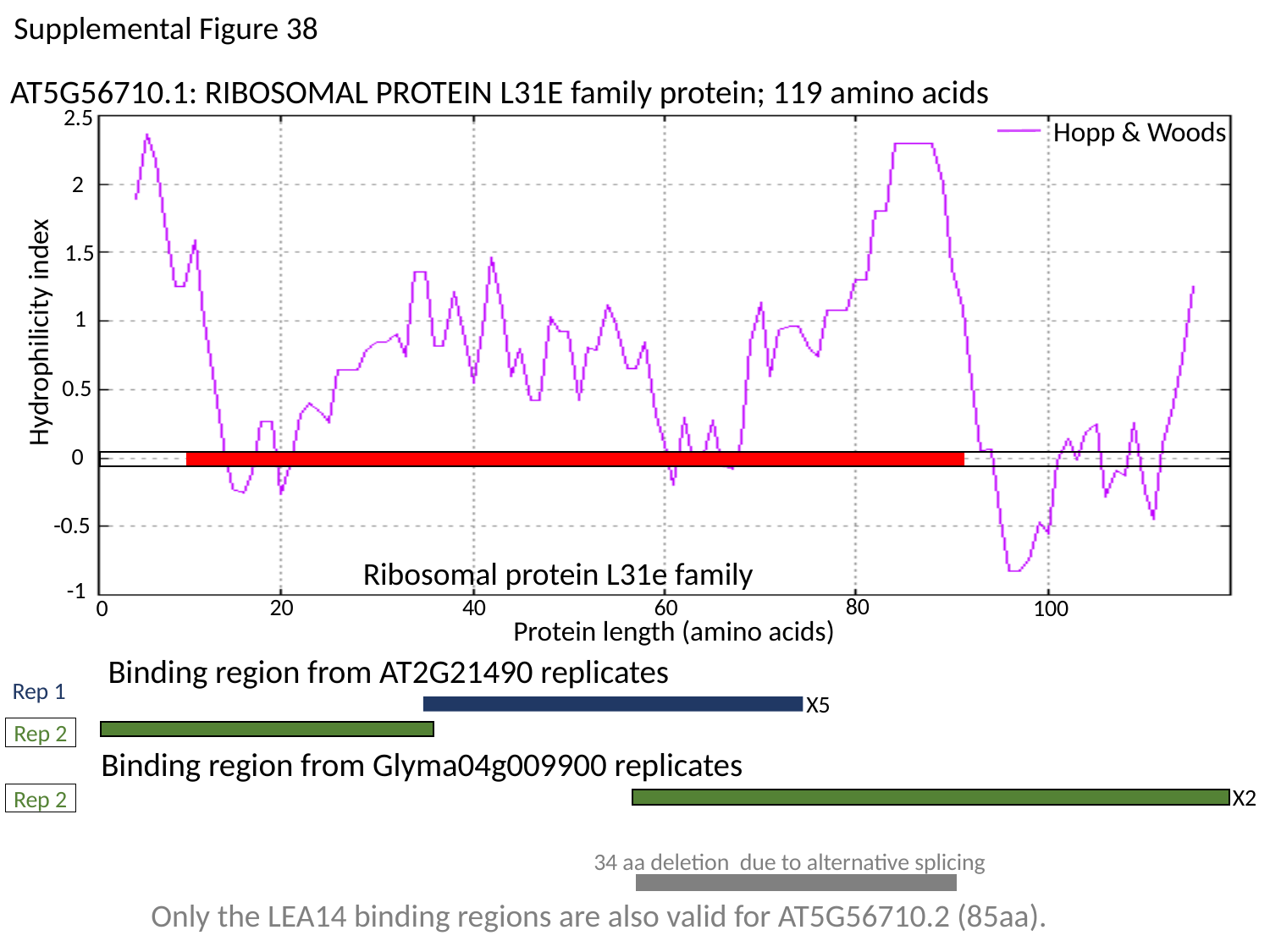

Supplemental Figure 38
AT5G56710.1: RIBOSOMAL PROTEIN L31E family protein; 119 amino acids
2.5
Hopp & Woods
2
1.5
1
Hydrophilicity index
0.5
0
MSEKKGRKEEVITREYTINLHRRLHKCTFKKKAPKAIKEIRKFAEKAMGTKDVRVDVKLNKQIWSKGIRGPPRRIRVRVARKRNDDEDAKEEFFSLVTVAEIPAEGLSGLGTKVIEEED
MSEKKGRKEEVITREYTINLHRRLHKCTFKKKAPKAIKEIRKFAEKAMGTKDVRVDVKLNKQIWSKGIRGPPRRIRVRVARKRNDDEDAKEEFFSLVTVAEIPAEGLSGLGTKVIEEED
-0.5
Ribosomal protein L31e family
-1
80
20
60
40
0
100
Protein length (amino acids)
Binding region from AT2G21490 replicates
Rep 1
X5
Rep 2
Binding region from Glyma04g009900 replicates
X2
Rep 2
MSEKKGRKEEVITREYTINLHRRLHKCTFKKKAPKAIKEIRKFAEKAMGTKDVRVDVKLNKQIWSKGIRGPPRRIRVRVARKRNDDEDAKEEFFSLVTVAEIPAEGLSGLGTKVIEEED
34 aa deletion due to alternative splicing
Only the LEA14 binding regions are also valid for AT5G56710.2 (85aa).

## Slide 39
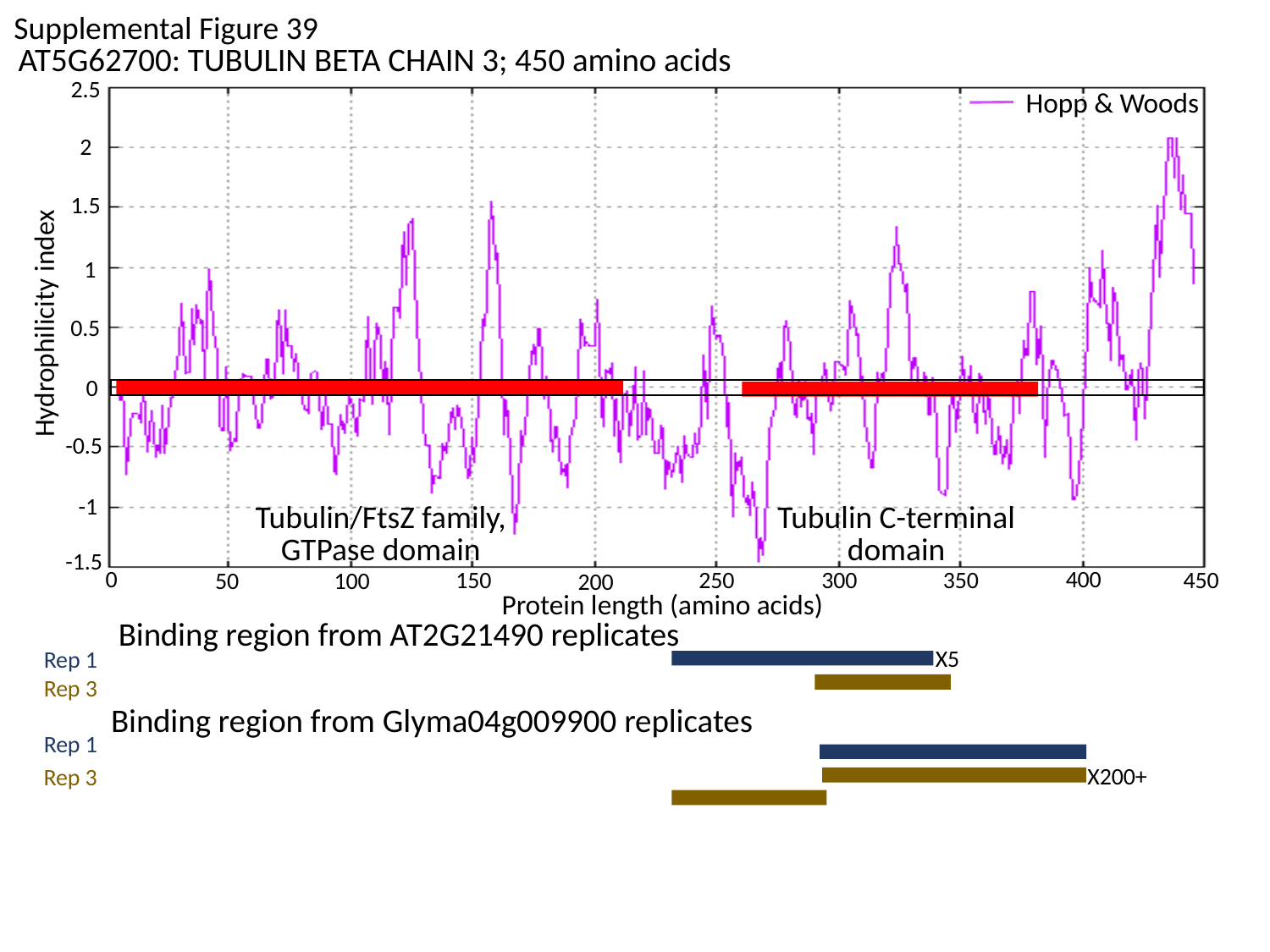

Supplemental Figure 39
AT5G62700: TUBULIN BETA CHAIN 3; 450 amino acids
2.5
Hopp & Woods
2
1.5
1
Hydrophilicity index
0.5
0
MREILHIQGGQCGNQIGAKFWEVVCAEHGIDPTGRYTGDSDLQLERINVYYNEASCGRFVPRAVLMDLEPGTMDSLRSGPYGQTFRPDNFVFGQSGAGNNWAKGHYTEGAELIDSVLDVVRKEAENCDCLQGFQVCHSLGGGTGSGMGTLLISKIREEYPDRMMLTFSVFPSPKVSDTVVEPYNATLSVHQLVENADECMVLDNEALYDICFRTLKLTTPSFGDLNHLISATMSGVTCCLRFPGQLNSDLRKLAVNLIPFPRLHFFMVGFAPLTSRGSQQYRSLTVPELTQQMWDSKNMMCAADPRHGRYLTASAMFRGKMSTKEVDEQMLNVQNKNSSYFVEWIPNNVKSTVCDIPPTGLKMASTFIGNSTSIQEMFRRVSEQFTAMFRRKAFLHWYTGEGMDEMEFTEAESNMNDLVSEYQQYQDATADEEGDYEDEEEGEYQQEEEY
-0.5
MREILHIQGGQCGNQIGAKFWEVVCAEHGIDPTGRYTGDSDLQLERINVYYNEASCGRFVPRAVLMDLEPGTMDSLRSGPYGQTFRPDNFVFGQSGAGNNWAKGHYTEGAELIDSVLDVVRKEAENCDCLQGFQVCHSLGGGTGSGMGTLLISKIREEYPDRMMLTFSVFPSPKVSDTVVEPYNATLSVHQLVENADECMVLDNEALYDICFRTLKLTTPSFGDLNHLISATMSGVTCCLRFPGQLNSDLRKLAVNLIPFPRLHFFMVGFAPLTSRGSQQYRSLTVPELTQQMWDSKNMMCAADPRHGRYLTASAMFRGKMSTKEVDEQMLNVQNKNSSYFVEWIPNNVKSTVCDIPPTGLKMASTFIGNSTSIQEMFRRVSEQFTAMFRRKAFLHWYTGEGMDEMEFTEAESNMNDLVSEYQQYQDATADEEGDYEDEEEGEYQQEEEY
-1
Tubulin/FtsZ family, GTPase domain
Tubulin C-terminal domain
-1.5
400
0
250
450
350
300
150
50
100
200
Protein length (amino acids)
Binding region from AT2G21490 replicates
X5
Rep 1
Rep 3
Binding region from Glyma04g009900 replicates
Rep 1
X200+
Rep 3
