# Supplementary material for: Dehydrin Client Proteins Identified Using Phage Display Affinity Selected Libraries Processed With Paired-End Phage Sequencing
Source: Mol Cell Proteomics. 2024 Oct 21;23(12):100867. doi: 10.1016/j.mcpro.2024.100867 (PMC11612773; doi:10.1016/j.mcpro.2024.100867)
Supplement: Supplemental Tables1_4 [file mmc3.docx]

**Supplemental Tables:**

**Supplemental Table 1: Primers used in this study.**

| Experiment | Primer name | Primer 5’→3’ |
| --- | --- | --- |
| Soybean Dehydrin GmPM12  (NM_001250385.1 Glyma04g009900 with a carboxyl terminal hexahistidyl tag | 955-NdeI | AACATATGGCTGAAGCACAACTACGAGACC |
|  | 956-XhoI (NO STOP) | TTCTCGAGGTGGGTGTGGTGGCCAGGCAATTTTTC |
| Arabidopsis Dehydrin At14  At2g21490 with a carboxyl terminal hexahistidyl tag | 953-NdeI | AACATATGGCGGATTTGAGGGACG |
|  | 954-PspXI (NO STOP) | TTACTCGAGTGGGTGGTTGTGGTTATGGTGG |
| Primers to the T7 10b coat protein gene flanking the 3’ end of the CDS | T7SelectUP primer | ggagctgtcgtattccagtcagg |
|  | T7Select  DOWN primer | aacccctcaagacccgtttagagg |
| Forward, bar-code-containing (yellow highlight) primers used for the first, limited-round PCR amplification of fourth round selected, phage sub-libraries. | F1-T7a | ACACTCTTTCCCTACACGACGCTCTTCCGATCTtcCCCggagctgtcgtattccagtcagg |
|  | F1-T7b | ACACTCTTTCCCTACACGACGCTCTTCCGATCTtcCGGggagctgtcgtattccagtcagg |
|  | F1-T7c | ACACTCTTTCCCTACACGACGCTCTTCCGATCTtcAACggagctgtcgtattccagtcagg |
|  | F1-T7d | ACACTCTTTCCCTACACGACGCTCTTCCGATCTtcAGCggagctgtcgtattccagtcagg |
|  | F1-T7e | ACACTCTTTCCCTACACGACGCTCTTCCGATCTtcAGAggagctgtcgtattccagtcagg |
|  | F1-T7f | ACACTCTTTCCCTACACGACGCTCTTCCGATCTtcACGggagctgtcgtattccagtcagg |
|  | F1-T7g | ACACTCTTTCCCTACACGACGCTCTTCCGATCTtcCCGggagctgtcgtattccagtcagg |
|  | F1-T7h | ACACTCTTTCCCTACACGACGCTCTTCCGATCTtcCAAggagctgtcgtattccagtcagg |
|  | F1-T7i | ACACTCTTTCCCTACACGACGCTCTTCCGATCTtcCAGggagctgtcgtattccagtcagg |
| Reverse primer used for the first, limited-round PCR amplification of fourth round selected, phage sub-libraries. | R2-T7 | **CGGTCTCGGCATTCCTGCTGAACCGCTCTTCCGATCT**aacccctcaagacccgtttagagg |
| Forward (PCR1) and reverse (PCR2) primers used for the second, limited-round PCR amplification (completing the Illumina adaptors) of fourth round selected, phage sub-libraries. | PE-PCR1 | AATGATACGGCGACCACCGAGATCTACACTCTTTCCCTACACGACGCTCTTCCGATCT |
|  | PE-PCR2 | CAAGCAGAAGACGGCATACGAGAT**CGGTCTCGGCATTCCTGCTGAACCGCTCTTCCGATCT** |
| Constructing GreenGate compatible CtP CDS for BiFC | LEA14 Forward BsaI | AACAGGTCTCAAACAATGGCGGATTTGAGGGACGAA |
|  | LEA14 Reverse BsaI | AACAGGTCTCAAGCCTGGGTGGTTGTGGTTATGGTG |
|  | PPR596 Forward BsaI | AACAGGTCTCAAACAATGTTCGCTCTTTCCAAGGTTTTAC |
|  | PPR596 Reverse BsaI | AACAGGTCTCAAGCCATCCAGAATATCAGAGATAGCTG |
|  | SESA4 Forward BsaI | AACAGGTCTCAAACAATGGCGAACAAGCTCTTCCTC |
|  | SESA4 Reverse BsaI | AACAGGTCTCAAGCCGTAGTAAGAAGGGATTGAAGG |
|  | RPL5A Forward BsaI | AACAGGTCTCAAACAATGGTGTTTGTGAAGTCCTCC |
|  | RPL5A Reverse BsaI | AACAGGTCTCAAGCCCTCTTCATCATCCTCATCATC |
|  | PAP12 Forward BsaI | AACAGGTCTCAAACAATGGCTTCCTTCACCTGTTCTTC |
|  | PAP12 Reverse BsaI | AACAGGTCTCAAGCCAGTGTAGTACTCCAACAAATAGC |
| Note: Lower case red sequence is homologous to the T7 10b coat protein gene 5’ of the site of foreign cDNA insertion. Lower case green sequence is homologous to the 3’ end of the T7 10b coat protein gene flanking the insertion site of the foreign cDNA. Yellow highlighted triplets constitute the bar code. The lower case “tc” preceding the bar code is a spacer to improve the quality of the bar code read close to the R1 primer. Primer F1-T7j has a portion highlighted in blue that is homologous to the 3’ end of PE-PCR1 while primer R2-T7 highlighted in pink is homologous to PE-PCR2. The non-highlighted 5’ portion of the primers PE-PCRx complete the illumina adaptors. Underlined nucleotides are restriction endonuclease sites. | | |

**Supplemental Table 2: Summary statistics for CLC Genomics Workbench read assembly.**

|  | **Arabidopsis LEAP well 1 (bar code AGC)** | | | | |
| --- | --- | --- | --- | --- | --- |
|  | Count | Percentage of reads | Average length | Number of bases | Percentage of bases |
|  |  |  |  |  |  |
| References | 70,380 | - | 1,233.27 | 86,797,462 | - |
| Mapped reads | 669,513 | 24.37% | 248.39 | 166,301,403 | 24.36% |
| Not mapped reads | 2,078,145 | 75.63% | 248.53 | 516,491,610 | 75.64% |
| Reads in pairs | 620,624 | 22.59% | 345.50 | 154,225,064 | 22.59% |
| Broken paired reads | 48,889 | 1.78% | 247.02 | 12,076,339 | 1.77% |
| Total reads | 2,747,658 | 100.00% | 248.50 | 682,793,013 | 100.00% |
|  | **Arabidopsis LEAP well 2 (bar code AGA)** | | | | |
|  | Count | Percentage of reads | Average length | Number of bases | Percentage of bases |
| References | 70,380 | - | 1,233.27 | 86,797,462 | - |
| Mapped reads | 357,032 | 15.20% | 248.38 | 88,680,407 | 15.19% |
| Not mapped reads | 1,991,608 | 84.80% | 248.52 | 494,956,633 | 84.81% |
| Reads in pairs | 324,926 | 13.83% | 336.71 | 80,744,111 | 13.83% |
| Broken paired reads | 32,106 | 1.37% | 247.19 | 7,936,296 | 1.36% |
| Total reads | 2,348,640 | 100.00% | 248.50 | 583,637,040 | 100.00% |
|  | **Arabidopsis LEAP well 3 (bar code ACG)** | | | | |
|  | Count | Percentage of reads | Average length | Number of bases | Percentage of bases |
| References | 70,380 | - | 1,233.27 | 86,797,462 | - |
| Mapped reads | 453,376 | 16.68% | 248.44 | 112,638,311 | 16.68% |
| Not mapped reads | 2,264,570 | 83.32% | 248.51 | 562,771,270 | 83.32% |
| Reads in pairs | 405,698 | 14.93% | 374.34 | 100,815,953 | 14.93% |
| Broken paired reads | 47,678 | 1.75% | 247.96 | 11,822,358 | 1.75% |
| Total reads | 2,717,946 | 100.00% | 248.50 | 675,409,581 | 100.00% |
|  | **Soybean LEAP well 1 (bar code CCC)** | | | | |
|  | Count | Percentage of reads | Average length | Number of bases | Percentage of bases |
| References | 70,380 | - | 1,233.27 | 86,797,462 | - |
| Mapped reads | 855,610 | 44.43% | 248.46 | 212,585,870 | 44.43% |
| Not mapped reads | 1,069,924 | 55.57% | 248.53 | 265,909,329 | 55.57% |
| Reads in pairs | 767,930 | 39.88% | 399.79 | 190,830,605 | 39.88% |
| Broken paired reads | 87,680 | 4.55% | 248.12 | 21,755,265 | 4.55% |
| Total reads | 1,925,534 | 100.00% | 248.50 | 478,495,199 | 100.00% |
|  | **Soybean LEAP well 2 (bar code CGG)** | | | | |
|  | Count | Percentage of reads | Average length | Number of bases | Percentage of bases |
| References | 70,380 | - | 1,233.27 | 86,797,462 | - |
| Mapped reads | 377,061 | 14.70% | 248.41 | 93,664,361 | 14.69% |
| Not mapped reads | 2,188,285 | 85.30% | 248.52 | 543,824,120 | 85.31% |
| Reads in pairs | 262,166 | 10.22% | 379.46 | 65,148,251 | 10.22% |
| Broken paired reads | 114,895 | 4.48% | 248.19 | 28,516,110 | 4.47% |
| Total reads | 2,565,346 | 100.00% | 248.50 | 637,488,481 | 100.00% |
|  | **Soybean LEAP well 3 (barcode AAC)** | | | | |
|  | Count | Percentage of reads | Average length | Number of bases | Percentage of bases |
| References | 70,380 | - | 1,233.27 | 86,797,462 | - |
| Mapped reads | 457,784 | 17.32% | 248.38 | 113,702,749 | 17.31% |
| Not mapped reads | 2,185,206 | 82.68% | 248.53 | 543,080,266 | 82.69% |
| Reads in pairs | 415,664 | 15.73% | 343.65 | 103,292,504 | 15.73% |
| Broken paired reads | 42,120 | 1.59% | 247.16 | 10,410,245 | 1.59% |
| Total reads | 2,642,990 | 100.00% | 248.50 | 656,783,015 | 100.00% |
|  | **BSA well 1 (bar code CCG)** | | | | |
|  | Count | Percentage of reads | Average length | Number of bases | Percentage of bases |
| References | 70,380 | - | 1,233.27 | 86,797,462 | - |
| Mapped reads | 601,712 | 27.16% | 248.28 | 149,394,922 | 27.13% |
| Not mapped reads | 1,613,832 | 72.84% | 248.58 | 401,167,762 | 72.87% |
| Reads in pairs | 538,298 | 24.30% | 502.88 | 133,767,053 | 24.30% |
| Broken paired reads | 63,414 | 2.86% | 246.44 | 15,627,869 | 2.84% |
| Total reads | 2,215,544 | 100.00% | 248.50 | 550,562,684 | 100.00% |
|  | **BSA well 2 (bar code CAA)** | | | | |
|  | Count | Percentage of reads | Average length | Number of bases | Percentage of bases |
| References | 70,380 | - | 1,233.27 | 86,797,462 | - |
| Mapped reads | 644,840 | 29.67% | 247.21 | 159,408,595 | 29.52% |
| Not mapped reads | 1,528,414 | 70.33% | 249.05 | 380,645,024 | 70.48% |
| Reads in pairs | 286,594 | 13.19% | 364.42 | 71,218,609 | 13.19% |
| Broken paired reads | 358,246 | 16.48% | 246.17 | 88,189,986 | 16.33% |
| Total reads | 2,173,254 | 100.00% | 248.50 | 540,053,619 | 100.00% |
|  | **BSA well 3 (bar code CAG)** | | | | |
|  | Count | Percentage of reads | Average length | Number of bases | Percentage of bases |
| References | 70,380 | - | 1,233.27 | 86,797,462 | - |
| Mapped reads | 1,684,314 | 36.04% | 248.15 | 417,960,644 | 35.99% |
| Not mapped reads | 2,989,020 | 63.96% | 248.70 | 743,362,855 | 64.01% |
| Reads in pairs | 1,311,938 | 28.07% | 389.92 | 326,016,593 | 28.07% |
| Broken paired reads | 372,376 | 7.97% | 246.91 | 91,944,051 | 7.92% |
| Total reads | 4,673,334 | 100.00% | 248.50 | 1,161,323,499 | 100.00% |

References: Total number of proteins annotated in the *Arabidopsis thaliana* proteome (Phytozome 12.1). Mapped reads: those reads, in-frame with the T7 coat protein, that encoded a legitimate *A. thaliana* protein. Unmapped reads: Reads in-frame with the virus coat protein for which a legitimate *A. thaliana* protein match could not be made (e.g. cloned into the virus T710B coat protein out-of-frame with the *A. thaliana* proteome.

**Supplemental Table 3: Molecular function of the dehydrin client proteins (CtPs) based on Gene Ontology.**

| \| Arabidopsis thaliana (REFERENCE) \|  \|  \| \| \| \| \| \| \| \| \| \| --- \| --- \| --- \| --- \| --- \| --- \| --- \| --- \| --- \| --- \| --- \| \| GO molecular function complete \| **Number of Arabidopsis constituents** \| **Number of CtPs out of 35** \| **Exp’d** \| \| **Fold enriched or depleted** \| \| **+/-** \| **raw P value** \| **FDR** \| \| \| mRNA binding \| 1129 \| 10 \| \| 1.44 \| \| 6.94 \| + \| 1.03E-06 \| \| 8.37E-04 \| \| 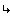RNA binding \| 1791 \| 14 \| \| 2.28 \| \| 6.13 \| + \| 1.66E-08 \| \| 5.41E-05 \| \| 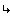nucleic acid binding \| 3781 \| 17 \| \| 4.82 \| \| 3.52 \| + \| 8.92E-07 \| \| 9.69E-04 \| \| 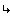organic cyclic compound binding \| 5272 \| 20 \| \| 6.73 \| \| 2.97 \| + \| 7.64E-07 \| \| 1.25E-03 \| \| 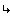**binding** \| 10487 \| 27 \| \| 13.38 \| \| 2.02 \| + \| 4.27E-06 \| \| 2.79E-03 \| |
| --- | --- | --- | --- | --- | --- | --- | --- | --- | --- | --- | --- | --- | --- | --- | --- | --- | --- | --- | --- | --- | --- | --- | --- | --- | --- | --- | --- | --- | --- | --- | --- | --- | --- | --- | --- | --- | --- | --- | --- | --- | --- | --- | --- | --- | --- | --- | --- | --- | --- | --- | --- | --- | --- | --- | --- | --- | --- | --- | --- | --- | --- | --- | --- | --- | --- | --- | --- | --- | --- | --- | --- | --- | --- | --- | --- | --- | --- |

**Exp’d: number of CtPs out of 35 expected in this category. +/-: Whether the CtPs in this category of Molecular Function exceed (+) or are fewer than (-), the number expected. FDR: False Discovery Rate.**

**Supplemental Table 4: Cellular components of the dehydrin CtPs based on Gene Ontology.**

| Arabidopsis thaliana  (REFERENCE) |  |  | | | | | |
| --- | --- | --- | --- | --- | --- | --- | --- |
| GO cellular component complete | **Number of Arabidopsis constituents** | **Number of CtPs out of 35** | **Exp’d** | **Fold enriched or**  **depleted** | **+/-** | **Raw P value** | **(FDR)** |
| 90S preribosome | 27 | 2 | 0.03 | 58.07 | + | 6.27E-04 | 3.63E-02 |
| 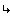preribosome | 85 | 3 | 0.11 | 27.67 | + | 1.93E-04 | 1.19E-02 |
| 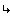ribonucleoprotein complex | 655 | 9 | 0.84 | 10.77 | + | 1.07E-07 | 3.53E-05 |
| 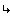**protein-containing complex** | 3070 | 14 | 3.92 | 3.57 | + | 1.14E-05 | 1.25E-03 |
| cytosolic large ribosomal subunit | 143 | 5 | 0.18 | 27.41 | + | 1.21E-06 | 2.98E-04 |
| 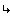large ribosomal subunit | 193 | 5 | 0.25 | 20.31 | + | 5.04E-06 | 7.09E-04 |
| 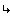ribosomal subunit | 317 | 5 | 0.4 | 12.36 | + | 5.22E-05 | 4.29E-03 |
| 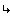ribosome | 400 | 6 | 0.51 | 11.76 | + | 1.13E-05 | 1.40E-03 |
| 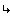intracellular non-membrane-bounded organelle | 2360 | 16 | 3.01 | 5.31 | + | 7.69E-09 | 7.58E-06 |
| 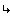**non-membrane-bounded organelle** | 2361 | 16 | 3.01 | 5.31 | + | 7.73E-09 | 3.81E-06 |
| 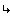cytosolic ribosome | 300 | 6 | 0.38 | 15.68 | + | 2.25E-06 | 4.44E-04 |
| 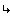**cytosol** | 2659 | 13 | 3.39 | 3.83 | + | 1.28E-05 | 1.26E-03 |
| nucleolus | 501 | 7 | 0.64 | 10.95 | + | 3.05E-06 | 5.01E-04 |
| 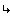nuclear lumen | 937 | 8 | 1.20 | 6.69 | + | 1.96E-05 | 1.76E-03 |
| 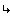intracellular organelle lumen | 1106 | 8 | 1.41 | 5.67 | + | 6.32E-05 | 4.49E-03 |
| 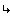organelle lumen | 1106 | 8 | 1.41 | 5.67 | + | 6.32E-05 | 4.15E-03 |
| 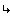**membrane-enclosed lumen** | 1106 | 8 | 1.41 | 5.67 | + | 6.32E-05 | 4.45E-03 |

**Exp’d: number of CtPs out of 35 expected in this category. +/-: Whether the CtPs in this category of Cellular components exceed (+) or are fewer than (-), the number expected. FDR: False Discovery Rate.**
