# Supplementary material for: Dehydrin Client Proteins Identified Using Phage Display Affinity Selected Libraries Processed With Paired-End Phage Sequencing
Source: Mol Cell Proteomics. 2024 Oct 21;23(12):100867. doi: 10.1016/j.mcpro.2024.100867 (PMC11612773; doi:10.1016/j.mcpro.2024.100867)
Supplement: Supplemental_TEXT_MASTER [file mmc5.docx]

**Supplemental Experimental Procedures**

**Defining soybean and *Arabidopsis* orthologous LEAPs**

We elected to use phage display to identify LEAP client proteins that interact with orthologous soybean and *Arabidopsis* LEAPs. To demarcate LEAP orthologs, LEAPs identified in soybean (Guo, Zhang et al. 2023) and retrieved from SoyBase (Version Williams 82, Assembly 4, Annotation 1 (Wm82.a4.v1) protein sequences) were compared to those identified in *Arabidopsis* (Hundertmark and Hincha 2008) and retrieved from Araport11 (Cheng, Krishnakumar et al. 2017) using Clustal Omega (Sievers, Wilm et al. 2011) and MEGA11 (Tamura, Stecher et al. 2021). The *Arabidopsis* list was augmented by one LEA 3 protein (AT3G12960), the ortholog of Glyma.08G172800 (Shih, Hsieh et al. 2010) totaling 52 proteins. The soybean list (Guo, Zhang et al. 2023) was curtailed by removing the Abscisic Acid-, Stress-, Ripening-Induced (ASR)-Like proteins (Glyma.20G167500, Glyma.10G224300, and Glyma.16G166600) based on community consensus (Jaspard, Macherel et al. 2012, Yacoubi, Hamdi et al. 2021). The soybean list was further reduced by removing Glyma.08G239400 which possesses no LEAP signature (InterProScan; (Jones, Binns et al. 2014)) and whose closest *Arabidopsis* ortholog (AT1G22600) was clearly classified as a non-LEAP (Hundertmark and Hincha 2008). Glyma.12G001600 was also removed. Both aforementioned proteins are classified as mitochondrial import inner membrane translocase proteins (InterProScan). Finally, the two *Arabidopsis* AtM proteins (AT2G41280, AT2G41260; (Raynal, Guilleminot et al. 1999)), for which there are no homologs outside of the Brassicaceae (Hundertmark and Hincha 2008), were included in the phylogenetic tree despite lacking soybean orthologs with which to match them. Their placement in a distinct clade in the tree, when compared to their placement in previously published trees (Hundertmark and Hincha 2008), served as a determination of the validity of the current phylogenetic model and was inclusive of all members of all valid LEAP families in both species.

The soybean list was augmented by the inclusion of Glyma.08G210100 whose closest *Arabidopsis* ortholog is AT1G01470.1 (Hundertmark and Hincha 2008), a LEA2 family protein. Proteins Glyma.19G114700, Glyma.17G187600, Glyma.16G037900, and Glyma.16G038000 (InterProScan Dehydrin (DHN) family LEAPs; IPR039285), were also added. Proteins Glyma.03G000800 and Glyma.16G221900, assessed as belonging to the LEA14-like superfamily (hidden Markov model library and genome assignments server; SSF117070 (Wilson, Pethica et al. 2009)) and further characterized as LEA2 family proteins (InterProScan; IPR004864), were also included totaling 79 proteins.

In cases where differential splicing events or alternative start- or termination-sites result in a multiplicity of different protein isoforms, the protein encoded by the first coding sequence was used. The 131 aligned LEAP orthologs were used in a neighbor-joining tree using maximum likelihood (see Statistical Rationale).
